# Embodying Time in the Brain: A Multi-Dimensional Neuroimaging Meta-Analysis of 95 Duration Processing Studies

**DOI:** 10.1007/s11065-023-09588-1

**Published:** 2023-03-01

**Authors:** Narges Naghibi, Nadia Jahangiri, Reza Khosrowabadi, Claudia R. Eickhoff, Simon B. Eickhoff, Jennifer T. Coull, Masoud Tahmasian

**Affiliations:** 1 Institute for Cognitive and Brain Sciences, Shahid Beheshti University, Tehran, Iran; 2 Faculty of Psychology & Education, Allameh Tabataba’i University, Tehran, Iran; 3 Institute of Neuroscience and Medicine Research, Structural and functional organisation of the brain (INM-1), Jülich Research Center, Jülich, Germany; 4 Institute of Clinical Neuroscience and Medical Psychology, Medical Faculty, Heinrich Heine University, Düsseldorf, Germany; 5 Institute of Neuroscience and Medicine Research, Brain and Behaviour (INM-7), Jülich Research Center, Wilhelm-Johnen-Straße, Jülich, Germany; 6 Institute for Systems Neuroscience, Medical Faculty, Heinrich-Heine University, Düsseldorf, Germany; 7 Laboratoire de Neurosciences Cognitives (UMR 7291), Aix-Marseille Université & CNRS, Marseille, France

**Keywords:** Time perception, Duration Processing, Timing, Embodiment, Activation likelihood estimation, Coordinate-based meta-analysis

## Abstract

Time is an omnipresent aspect of almost everything we experience internally or in the external world. The experience of time occurs through such an extensive set of contextual factors that, after decades of research, a unified understanding of its neural substrates is still elusive. In this study, following the recent best-practice guidelines, we conducted a coordinate-based meta-analysis of 95 carefully-selected neuroimaging papers of duration processing. We categorized the included papers into 14 classes of temporal features according to six categorical dimensions. Then, using the activation likelihood estimation (ALE) technique we investigated the convergent activation patterns of each class with a cluster-level family-wise error correction at p < 0.05. The regions most consistently activated across the various timing contexts were the pre-SMA and bilateral insula, consistent with an embodied theory of timing in which abstract representations of duration are rooted in sensorimotor and interoceptive experience, respectively. Moreover, class-specific patterns of activation could be roughly divided according to whether participants were timing auditory sequential stimuli, which additionally activated the dorsal striatum and SMA-proper, or visual single interval stimuli, which additionally activated the right middle frontal and inferior parietal cortices. We conclude that temporal cognition is so entangled with our everyday experience that timing stereotypically common combinations of stimulus characteristics reactivates the sensorimotor systems with which they were first experienced.

**Supplementary Information:**

The online version contains supplementary material available at 10.1007/s11065-023-09588-1.

## Introduction

The ubiquitous presence of time in diverse aspects of an organism’s life is a clear sign of the intertwined relationship between time and everyday action and perception. Time is a container for all of our experience and has close connections to most cognitive processes. Motion perception (Ayhan & Ozbagci, 2020; Brown, 1995; Li et al., 2021; Yamamoto & Miura, 2016), motor control (Gavazzi et al., 2013; Wiener, Zhou, Bader, & Joiner, 2019), language (Gordon et al., 2015; Goswami, 2019; Kotz & Schwartze, 2010; Schirmer, 2004), memory (Cumming et al., 2015; Matthews & Meck, 2016; Polti, Martin, & van Wassenhove, 2018), causal inference (Woods et al., 2014), decision-making (Wittmann & Paulus, 2008), and even self-awareness (Droit-Volet & Dambrun, 2019; Wittmann, Jokic, & Pfeifer, 2019), all have multimodal interactions with our sense of time. Moreover, time mediates these cognitive processes on a wide range of timescales and sensory modalities. There is abundant evidence for partial dissociation of timing mechanisms as a function of context (for a review, please see Buhusi & Meck, 2005; Paton & Buonomano, 2018). Therefore, the influence of such a diverse array of contextual factors on duration processing impedes a unified understanding of this multifaceted phenomenon.

Despite extensive neuroimaging investigations into the neural correlates of temporal processing, major inconsistencies remain to be addressed. Several integrational studies have tried to eliminate the neuroanatomical inconsistencies resulting from spurious findings of individual experiments that employed disparate experimental designs, various analytical procedures, and small sample sizes. Specifically, these studies adopted a conceptual approach by defining pivotal temporal categories that might disentangle the variability arising from diverse experimental paradigms and task parameters. In this regard, the categorical divisions of sub- vs. supra-second durations, perceptual vs. motor tasks, discrete vs. continuous (or sequential) stimulus presentation, internally-based vs. externally-cued timing, and implicit vs. explicit measures have been qualitatively and quantitatively demonstrated to be beneficial in reducing the extensive variance observed across the literature (Coull & Nobre, 2008; Lewis & Miall, 2003b; Nani et al., 2019; Schwartze, Rothermich, & Kotz, 2012; Teghil et al., 2019; M. Wiener, P. Turkeltaub, & H. B. Coslett, 2010a; M. Wiener, P. E. Turkeltaub, & H. B. Coslett, 2010b). Each integrational study has shed light on a part of the problem from one or two angles, but, given its ubiquity, timing should be treated as a multi-dimensional process that depends on a more complex set of factors. As a result, despite providing valuable insights into the differential involvement of certain structures in distinct components or “classes” of a proposed category, no previous study has been able to unambiguously identify a unified timing system. For instance, previous reviews and meta-analyses (Ivry, 1996; Nani et al., 2019; Schwartze et al., 2012; Wiener et al., 2010a) have consistently related the cerebellum to the processing of sub-second durations. However, there is evidence that the cerebellum is also involved in certain timing tasks in the supra-second range (Beudel et al., 2008; Kawashima et al., 2000; Ohmae, Kunimatsu, & Tanaka, 2017; Petter, Lusk, Hesslow, & Meck, 2016), which cannot be accounted for by categorizations based on only one or two dimensions. Accordingly, we propose a multi-factor classification system, incorporating an extensive set of categorical dimensions, which provides a deeper functional understanding of each brain region. Furthermore, the resulting mesh of complementary factors facilitates subsequent inference about the role of each brain area in timing.

The current analysis aims to address the above limitation by investigating robust findings across the timing literature as a function of six separate dimensions. This multi-dimensional investigation provides greater insight into the main taxonomies of time that have been proposed and, subsequently, identifies the temporal contexts most probably activating each brain region. Specifically, our categorical dimensions comprise factors related to the stimuli: stimulus duration, stimulus modality, and stimulus contiguity; as well as factors related to the task: sensorimotor processing, task goal, and stringency of the control task. Based on these six dimensions, we coded studies in the available literature into a total of 16 groups, including short, medium, and long durations; auditory, visual, and tactile sensory modalities; trajectory, single-interval, and sequence stimuli; perceptual and motor tasks; quantification and prediction task goals; and studies that controlled for task structure, stimulus dynamics or task difficulty, or for all three.. Unfortunately, the number of experiments in the tactile and trajectory classes was not adequate to be analyzed. It should be noted that the groups within a single dimension are mutually exclusive. For instance, the stimulus duration dimension comprises three distinct groups or “classes”: <500ms, 500-1500ms, > 1500ms. We employed Activation Likelihood Estimation (ALE), a well-established coordinate-based meta-analysis (CBMA) technique, to quantitatively consolidate neuroimaging findings within each group. Although the categorical dimensions of stimulus duration, sensorimotor processing, and stimulus contiguity have already been incorporated into several prior meta-analyses of the field (Lewis & Miall, 2003b; Nani et al., 2019; Schwartze et al., 2012; Wiener et al., 2010a), we include these categories again, in order to provide a point of comparison with the existing literature. In addition, rather than the sub- versus supra-second division used previously, which may be rather artificial, we aimed to highlight the brain regions more specifically contributing to the processing of short, medium, and long durations, along a functional spectrum of highly automatic to highly controlled timing mechanisms. In order to do so, we chose the duration boundaries so that only a minimum number of experiments were classified as short or long. This choice of boundaries also parallels the proposed boundaries at which distinct timing mechanisms, namely automatic, cognitively controlled, and verbal counting strategies (Buonomano et al., 2009; Grondin, Ouellet, & Roussel, 2004; R. M. C. Spencer, Karmarkar, & Ivry, 2009), come into play. Duration processing studies have also already been categorized according to task goal (Schwartze et al., 2012; Wiener et al., 2010a, b). Here, instead of the implicit/ explicit terminology (Coull & Nobre, 2008), we use a less ambiguous definition to classify experiments more objectively according to whether participants had to provide a quantitative measure of duration or whether they had to use temporal information to predict when an event would appear. Although some individual experiments (e.g., Araneda, Renier, Ebner-Karestinos, Dricot, & De Volder, 2017; Shih, Kuo, Yeh, Tzeng, & Hsieh, 2009) have examined the effect of stimulus modality on the neural correlates of duration processing, this categorical dimension is absent from integrational studies. This might be related to the implicit assumption that irrespective of the stimulus modality, the processing of duration information is subserved by dedicated modality-independent central structures. Finally, we incorporated a further dimension related to the control task to identify brain regions that were more reliably activated by timing processes rather than non-temporal cognitive task demands. A detailed description of each dimension is available in Sect. 2.2 and in appendix 1 of the supplementary materials.

Our overall aim in the current study was to provide a more comprehensive functional dissection of timing experiments so as to better disentangle the pattern of neural activations associated with different contextual properties of duration processing. In this regard, following the methodological recommendations of the Preferred Reporting Items for Systematic reviews and Meta-Analyses (PRISMA; Page et al., 2021) and best-practice recommendations for neuroimaging meta-analyses (Müller et al., 2018; Tahmasian et al., 2019) in obtaining papers and extracting the required information, we organized the neuroimaging findings of 95 duration processing studies according to six categorical dimensions and conducted 14 ALE analyses to identify robust neural activations. This extensive set of analyses provided a multi-dimensional perspective of the neural substrates of duration processing. By identifying anatomical commonalities and specificities across categories, we hoped this multi-dimensional perspective would shed some light on the functional contribution of each region. The multi-dimensional approach also provides an opportunity to compare how well each categorical dimension identifies neural structures that are specifically activated by its temporal characteristics and thus how effective it is as a taxonomic classification (Meck & Ivry, 2016; Paton & Buonomano, 2018). The focus of this article is on duration processing, and we do not cover any other forms of temporal processing, such as temporal order or simultaneity judgments.

## Methods

### Study Selection and Data Management

**Fig. 1:**
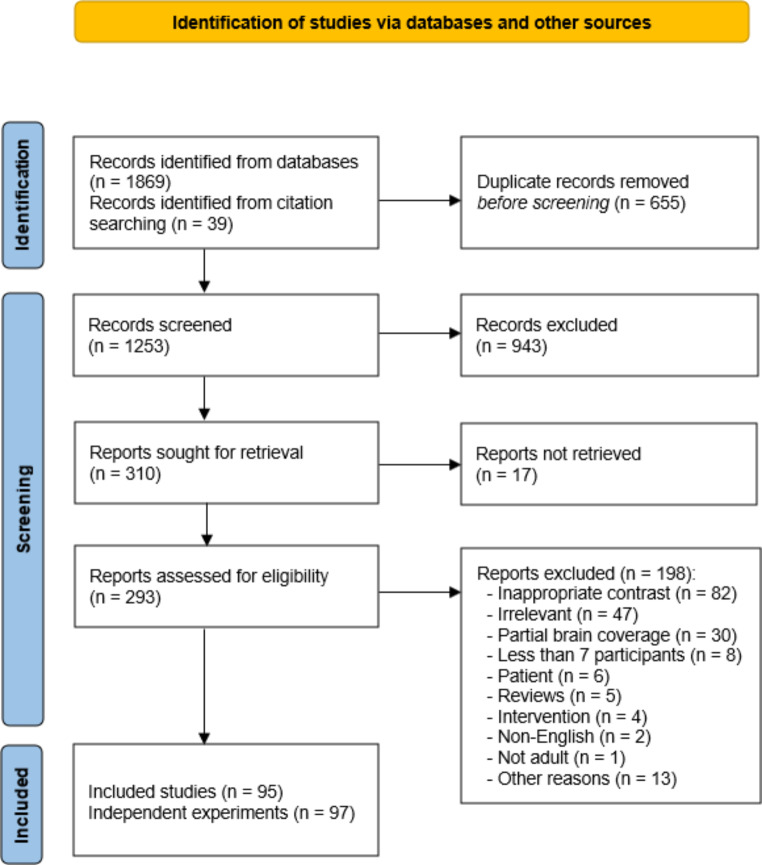
Flow diagram of study selection

Following the recent best-practice guidelines for neuroimaging meta-analyses (Müller et al., 2018; Tahmasian et al., 2019), we performed multiple ALE meta-analyses on neuroimaging findings of the duration processing literature. The search strategy and study selection were arranged according to the PRISMA guidelines (Page et al., 2021). More detailed information on the implementation of PRISMA items is available in the appendix 1 and Tables 17 and 18 of the supplementary materials. In order to collect neuroimaging studies of temporal processing, a search of PubMed, Scopus, and Web of Science, with no restrictions on the date of publication, was performed in April 2020 and enhanced with reference tracing of the retrieved articles. Figure 1 provides a flow diagram of the records in the study selection procedure. The detailed keyword string used to search the above databases can be found in Appendix 1 of the supplementary materials.

After removing the duplicates, we obtained 1214 records from the database search and 39 additional papers from other sources (Fig. 1, supplementary Table 1). We included English peer-reviewed articles on healthy human adults that used functional neuroimaging techniques to investigate the neural correlates of timing. The following exclusion criteria were used to exclude inappropriate studies:

- Case reports, letters to editors, and studies reporting no original data.
- Comparison to rest or any other passive conditions.
- Experiments with partial brain coverage, e.g., using region of interest (ROI), or small volume correction (SVC), as recommended previously (Müller et al., 2018; Tahmasian et al., 2019).
- Experiments comparing the post- and pre-intervention conditions (e.g., drug administration, temporal illusion).
- Experiments with less than seven participants.
- Studies that did not report coordinates in the standard space.

Based on the selection criteria, two independent investigators (N.N. and N.J.) assessed the obtained papers in two steps: (1) screening all 1253 abstracts; (2) full-text assessment of the 293 potential documents for eligibility. In this stage, all identified discrepancies were resolved according to the inclusion/exclusion criteria.

Three independent investigators (N. N., N. J., and J. C.) extracted and checked the required data, including the number of participants, reported peak coordinates in the standard spaces of Talairach (Talairach & Tournoux, 1988) or MNI (Evans et al., 1993), as well as categorical dimensions of stimulus duration, stimulus modality, stimulus contiguity, sensorimotor processing, task goal, and control task stringency. Any discrepancy that arose in this stage was then referred to J. C. to be judged.

The coordinates obtained in Talairach space were subsequently transformed into MNI space for analysis (Lancaster et al., 2007). As a technical distinction, it should be noted that throughout this paper, the word “study” refers to a scientific publication, and the word “experiment” represents an individual contrast (e.g., timing task > control task, or difficult > easy timing task). Of note, two of the studies (Chen et al., 2008; Coull & Nobre, 1998) reported independent experiments from two sets of distinct participants. Therefore, the 95 eligible papers included 97 independent experiments. The importance of this distinction between the terms, “experiment” and “study”, is that ALE algorithm implicitly assumes the included studies as independent sources of activation foci (Turkeltaub et al., 2012). However, when a paper recruits a single group of subjects and reports either multiple task contrasts or multiple analyses of a single task, this assumption can no longer be considered valid. In such cases, since we preferred not to exclude any information, we followed the organizational approach proposed by Turkeltaub et al. (2012). Accordingly, in order to make sure each group of participants contributed only once per analysis, in each analysis of a single characteristic, we merged experiments that varied in any of the other characteristics. As an example, a study may have contained four experiments that varied in terms of stimulus duration (short/long) and sensory modality (visual/auditory) dimensions, all of which were measured in a single group of participants. For the ALE analysis of experiments with short duration, sensory modality is a non-relevant dimension, and so its two classes (visual and auditory) should be merged so that both are unified in a group of short duration experiments.

In order to assess the quality of the included studies, we used a modified version of the 10-point checklist incorporated by previous neuroimaging meta-analyses (Z.-Q. Chen et al., 2015; Kamalian et al., 2022; Strakowski, DelBello, Adler, Cecil, & Sax, 2000; Su et al., 2021). Since, in addition to the demographic properties and imaging methodology, the original checklist also concerns with the assignment of participants into clinical and control groups, we replaced two of the clinically-specific items with another one related to the minimum sample size so that the overall score would be limited to a maximum of 9. The quality assessment criteria and score for each included study can be found in supplementary Tables 19 and 1, respectively.

### Data Classification Strategy

In the next step, for each of the six dimensions, we coded the selected experiments according to the following strategy. We categorized stimulus duration into short, medium, and long duration classes, which comprised experiments in the range of < 500 ms, 500–1500 ms, and > 1500 ms, respectively. According to the stimulus modality dimension, the included experiments used visual, auditory, or tactile stimulation. Since there were only five experiments with tactile stimulation, which was far below the minimum threshold for the ALE analysis (Eickhoff et al., 2016), this class was eliminated from the analyses. The stimulus contiguity dimension segregated the experiments according to whether stimuli were presented singly, in a sequence, or as a dynamic trajectory. The single-interval stimuli were separated from adjacent stimuli by a variable inter-stimulus interval and usually required a response before the next duration to-be-estimated was presented. Duration discrimination, temporal reproduction, and cued reaction time tasks are instances of tasks with single-interval stimuli. On the other hand, sequenced stimuli were presented consecutively in sets of three or more, separated by fixed or temporally structured intervals. Rhythm reproduction and rhythmic monitoring are examples of tasks with sequenced stimuli. The trajectory stimuli, consisting of moving visual objects such as those presented in collision and time to contact judgment tasks, were discarded from the analysis due to an insufficient number of experiments (13 experiments) and, therefore, low reliability. Motor and perceptual timing tasks were utilized as segregating factors for the sensorimotor categorical dimension. We labeled experiments as using motor timing tasks if the duration or onset of the motor response itself had to be accurately timed. On the other hand, if tasks required a choice response (e.g., yes/no or shorter/longer) that served merely to index the participant’s judgment of a timed stimulus, we labeled experiments as using perceptual timing tasks. We also categorized temporal tasks according to one of two goals: (1) quantification or (2) prediction. The quantification tasks required that duration was *overtly estimated*, usually as compared to other externally cued or internally represented durations: in these experiments, the task goal was primarily temporal in nature. By contrast, prediction tasks allowed the participant to *make use of temporal information* or patterns to predict the onset of an upcoming event so as to respond to it more quickly or accurately: the task goal in these experiments was sensorimotor. Although we excluded experiments that compared timing tasks to a rest condition, there were still many studies that controlled processes of non-interest rather poorly. We therefore applied further constraints to distinguish between these studies and those with more elegant designs that controlled for most of the non-timing processes. As a rule of thumb, we classified experiments according to the following criteria. The minimum level of control required studies to incorporate identical task structure and stimulus presentation across the compared conditions (in order to control for sensorimotor, mnemonic, attentional, and decisional aspects of the task). A higher level of control was achieved if, in addition to the similitude of the task structure and stimulus presentation, the compared conditions were matched in terms of either stimulus dynamics (to control for the working memory and sustained attention demands of processing duration) or task performance (to control for task difficulty). We define the highest level of control as simultaneously meeting all three conditions described above. With these definitions, we labeled studies conforming to the above-mentioned criteria as having a task control, a cognitive load control, or a stringent control, respectively. Further explanation about the rationale behind the above classifications and their similarities with previous categorizations is available in appendix 1 in the supplementary materials.

Using the ALE technique, each of the above classes was then assessed for neuroanatomical convergence across the included experiments. To investigate any possible convergence of findings across the entire timing field, we also conducted an ALE analysis on all eligible studies, which were grouped together in an all-effects category. For studies with multiple experiments obtained from a single group of participants, we followed the pooling approach suggested by Turkeltaub et al. (2012). A detailed explanation of the merging procedure is also available in the data extraction and management section of appendix 1.

### Activation Likelihood Estimation (ALE)

In order to test for significant convergence across studies, we used the revised version of ALE, implemented in MATLAB (Eickhoff et al., 2012). ALE is a standard statistical method for coordinate-based meta-analysis (CBMA) of neuroimaging data, identifying brain areas for which convergence across the included imaging experiments is higher than would be expected if results were randomly distributed. For further information about the ALE analysis, please see appendix 1 in the supplementary materials.

In the current study, in order to correct for multiple comparisons of statistical tests and avoid false-positive findings, we set the statistical significance threshold to p < 0.05 family-wise error at the cluster-level (cFWE), with a cluster forming threshold of p < 0.001, as suggested previously (Eickhoff et al., 2012). ALE analysis has been empirically proven to be able to control the influence of any individual experiment as long as a minimum of 17 experiments are included in the analysis (Eickhoff et al., 2016). Further information on sensitivity and power estimates of ALE analysis can be found in S. B. Eickhoff et al. (2016).

## Results

The 95 included publications comprised a total of 121 experiments and 1505 participants (supplementary Table 1). The anatomical labels of identified clusters were assigned based on the third version of the SPM Anatomy toolbox (Eickhoff et al., 2005). A more detailed description of the number of experiments included in each analysis and results of the conjunction analyses between classes of single dimensions can be found in appendix 2 of the supplementary materials. Further details on the center coordinates of each cluster, cluster size, contributing articles, and the proportional contribution from classes of each categorical dimension to the cluster are available in supplementary Tables 2–16.

### Duration Processing Literature Convergences (All-Effects Analysis)

**Fig. 2:**
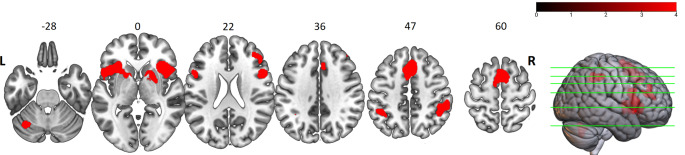
**Regions of convergent activation across the entire timing literature (All-effects analysis) (p < 0.05, family-wise error correction at cluster level).** L stands for left; and R for right

The all-effect analysis, merging all findings from 97 independent experiments, resulted in eight separate clusters. These were located in the supplementary motor area (SMA; including the pre-SMA and SMA-proper) extending towards the paracingulate gyrus (PCG), bilateral insula extending to the opercular region of the inferior frontal gyrus (IFG), ventrolateral part of the premotor cortex (PMCv), and left putamen, right middle frontal gyrus (MFG, BA 45, 46), right dorsal striatum (DST; including the putamen and parts of the caudate and globus pallidus), right inferior parietal lobule (IPL), left inferior parietal sulcus (IPS), and left cerebellum crus I (max p-value for cFWE < 0.0001; Fig. 2). More detailed information is available in appendix 2 and Table 2 of the supplementary material.

### Stimulus Duration Convergences

**Fig. 3:**
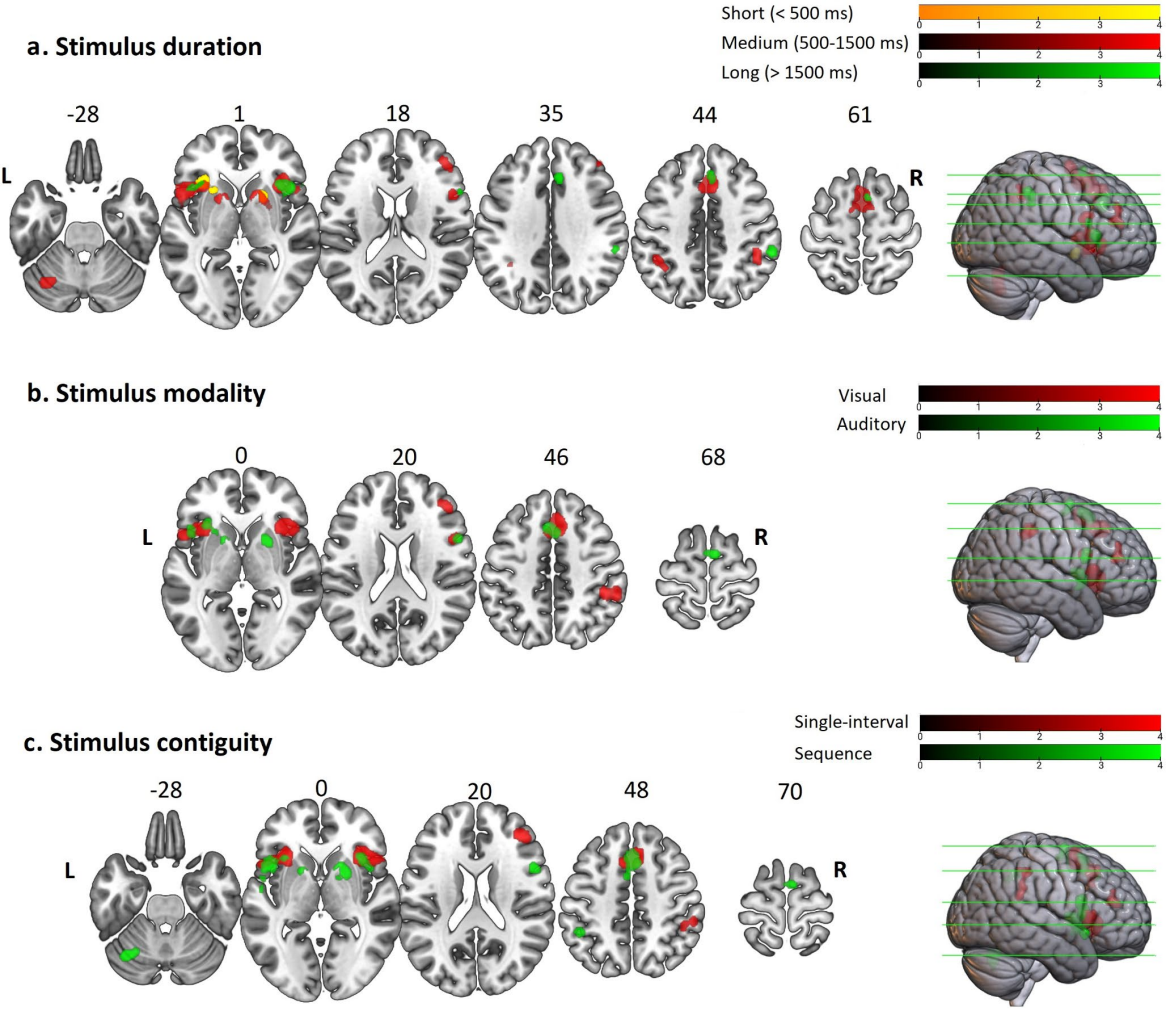
**Regions of convergent activation across groups of experiments classified according to the stimulus characteristics (p < 0.05, family-wise error correction at cluster level).** Results of the sub-analyses categorized according to (a) the stimulus duration dimension into the short (orange), medium (red), and long (green) range classes; (b) stimulus modality dimension into the visual (red) and auditory (green) modality classes; and (c) stimulus contiguity dimension into the single interval (red) and sequence (blue) classes. L stands for left; and R for right

The left insular cortex was the only region conjointly activated by the three duration ranges. The pre-SMA, PCG, right insula, IFG, and PMCv were activated by both medium- and long-range durations. The bilateral putamen were found to be activated by both the short- and medium-range durations (although there was no overlap in the left-lateralized clusters). Furthermore, the SMA-proper, right MFG, left IFG extending toward the PMCv, bilateral IPS, and left cerebellar activations were found to be specific to the medium-range durations and the right IPL cluster to the long-range durations. In all the duration-based analyses, the maximum p-value for family-wise error correction at cluster level was less than 0.0001. Figure 3a illustrates the axial view of the significant clusters obtained from the duration range analyses on a single brain template. More detailed information on the peak coordinates of these clusters, their cluster sizes, anatomical labels, and the experiments and parameters contributing to each cluster are available in appendix 2 and supplementary Tables 3, 4, and 5.

### Stimulus Modality Convergences

The visual and auditory stimuli were found to conjointly activate the pre-SMA, PCG, left insula, and right opercular IFG extending toward the PMCv. Additional right-lateralized regions of activation were found in the MFG (BA 45, 46), insula, and IPS for visual stimuli, and in the SMA-proper and bilateral dorsal striatum for auditory stimuli (Fig. 3b; appendix 2; supplementary Tables 6 and 7). In both visual and auditory analyses, the maximum p-value for family-wise error at cluster level was less than 0.0001.

### Stimulus Contiguity Convergences

Activations in the pre-SMA extending toward the PCG, bilateral insula, and IFG were common to both single-interval and sequential stimuli. The single-interval analysis yielded additional clusters in the right MFG (BA 45, 46) and IPL. On the other hand, activations specific to sequential stimuli were located in the SMA-proper, right IFG extending towards the PMCv, left IPS, bilateral dorsal striatum, the anterior portion of superior temporal gyrus (STG), left PMCv, and left cerebellum. Maximum p-value for family-wise error at cluster level was less than 0.0001 in both analyses. Figure 3c illustrates common and differential activations of single-interval and sequential stimuli. More detailed information is available in the supplementary materials (appendix 2; Tables 8 and 9).

### Sensorimotor Convergences

**Fig. 4:**
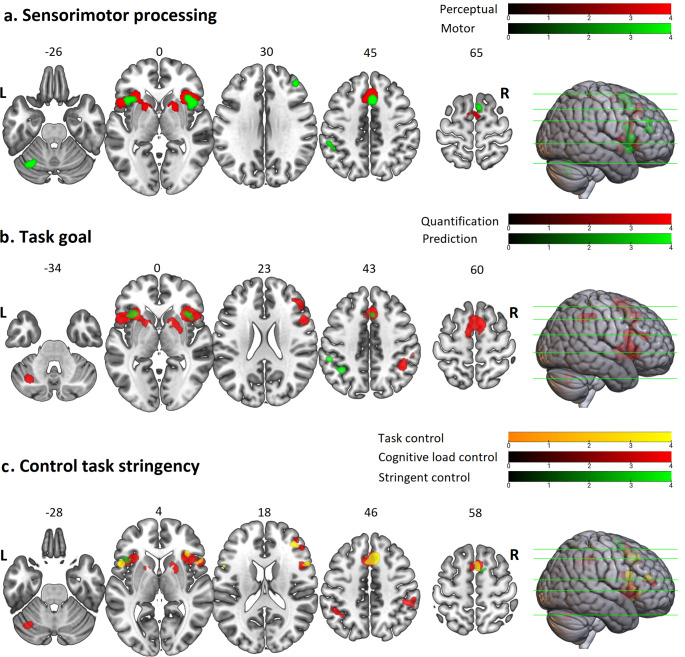
**Regions of convergent activation across groups of experiments classified according to the task characteristics (p < 0.05, family-wise error correction at cluster level).** Results of the sub-analyses categorized according to (a) the sensorimotor dimension into perceptual (red) and motor (green) classes; (b) task goal dimension into quantification (red) and prediction (green) classes; and (c) control task stringency dimension into task control (orange), cognitive load control (red); and stringent control (green) classes. L stands for left; and R for right

Both perceptual and motor timing studies commonly activated the pre-SMA extending toward the PCG and bilateral insula extending toward the IFG and right PMCv. Perceptual timing experiments additionally activated the SMA-proper, left PMCv, and bilateral dorsal striatum, while motor timing experiments additionally activated the right MFG, left IPS, anterior portion of STG, and left cerebellum (Fig. 4a; appendix 2; supplementary Tables 10 and 11). Maximum p-value for family-wise error at cluster level was less than 0.0001 in both analyses.

### Task goal Convergences

Analyses of experiments requiring quantification or prediction of duration mutually converged in a pre-SMA cluster extending to the PCG and in the bilateral insula. Quantification of duration additionally activated the SMA-proper, right-lateralized MFG and IPS, bilateral IFG extending toward PMCv, and bilateral DST, and left cerebellum (Fig. 4b). The only cluster of prediction-specific convergence was in the left IPS (Fig. 4b; appendix 2; supplementary Tables 12 and 13). In both quantification and prediction analyses, maximum p-value for family-wise error at cluster level was less than 0.0001.

### Control task Stringency Convergences

The three levels of control for non-timing processes were found to commonly converge in a very tiny area of the left opercular IFG. If control and timing tasks were matched simply in terms of basic sensorimotor characteristics, we found additional areas of convergent activation in the pre-SMA extending toward the PCG, and in right-lateralized MFG, IFG, PMCv, and insula (Fig. 4c). If control and timing tasks were matched on either task performance or the use of dynamic/continuous stimuli, additional areas of activation were found in the pre-SMA/PCG, right MFG, IFG, PMCv, bilateral insula, DST, IPS, and left cerebellum (Fig. 4c). However, if control tasks were matched on all three criteria, we found additional areas of activation in the pre-SMA and left insula only (Fig. 4c). Maximum p-value for family-wise error at cluster level was less than 0.0001 in all the analyses. Further information is available in the supplementary materials (appendix 2 and Tables 14 and 15, and 16).

## Discussion

We aimed to comprehensively explore the effect of experimental constraints on the neural underpinnings of duration processing by conducting 14 separate subanalyses, organized according to six orthogonal categories. To date, this is the largest meta-analysis of the field, with 95 papers included. From these analyses, a set of brain structures including the pre-supplementary motor area (pre-SMA), insula, inferior frontal gyrus (IFG), inferior parietal lobule (IPL), dorsal striatum (DST), and cerebellum were identified to be activated in duration processing. Importantly, by identifying anatomical commonalities and specificities across categories, we found three major activation patterns as following: (1) consistent with many previous reviews and meta-analyses (Nani et al., 2019; Teghil et al., 2019; Wiener et al., 2010a) the neural structures most often activated across various timing contexts were the pre-SMA and bilateral insula extending into IFG, which thus constitute a core timing system; (2) the DST and SMA-proper were selectively activated by timing of auditory and sequential stimuli; and (3) the right frontoparietal network, including the MFG and IPL, were selectively activated by timing of visual and single-interval stimuli.

### Timing in the Framework of Embodied Cognition

The overall results, summarized in Table 1, demonstrate that there is no central timing system for duration processing that is consistently engaged whatever the experimental context. Nevertheless, consistent with previous works (Merchant et al., 2013; Nani et al., 2019; Teghil et al., 2019; Wiener et al., 2010a), we identified the widespread presence of a set of structures traditionally implicated in motor processing, including the pre-SMA, SMA proper, premotor cortex, DST, and cerebellum, across various timing conditions. Strikingly, these regions were activated during both motor *and* perceptual timing tasks. Accordingly, irrespective of the sensorimotor dimension of the temporal task, the representation of duration during motor or perceptual timing relies on motor structures. Several lines of behavioral evidence (De Kock et al., 2021; Tomassini & Morrone, 2016; Wiener et al., 2019; Yokosaka, Kuroki, Nishida, & Watanabe, 2015) further support the essential contribution of motor processes to perceptual timing.

**Table 1:** A summary of the analyses conducted on classes of the categorical dimensions

Class	Number of experiments	Maximum $\mathbf{p}-\mathbf{v}\mathbf{a}\mathbf{l}\mathbf{u}\mathbf{e}$ (cFWE)	SMA proper	preSMA	R MFG	R IFG	L IFG	L INS	R INS	R IPC	L IPC	L DST	R DST	L CBL
ALE analysis of **all temporal processing experiments included**														
All	97	0.000	x	x	x	x	x	x	x	x	x	x	x	x
ALE analyses based on the **stimulus duration**														
Short (< 500 ms)	19	0.001						x				x	x	
Medium (500–1500 ms)	58	0.000	x	x	x	x	x	x	x	x	x	x	x	x
Long (> 1500 ms)	19	0.000		x		x		x	x	x				
ALE analyses based on the **sensory modality** of the stimuli														
Visual	57	0.000		x	x	x	x	x	x	x				
Auditory	32	0.000	x	x		x		x				x	x	
ALE analyses based on the **stimulus contiguity**														
Single interval	46	0.000		x	x	x	x	x	x	x				
Sequence	39	0.000	x	x		x	x	x	x		x	x	x	x
ALE analyses based on the **sensorimotor processing**														
Perceptual	55	0.000	x	x		x	x	x	x			x	x	
Motor	48	0.000		x	x	x	x	x	x		x			x
ALE analyses based on the **task goal**														
Quantification	65	0.000	x	x	x	x	X	x	x	x		x	x	x
Prediction	32	0.000		x				x	x		x			
ALE analyses based on the **stringency of control task**														
Task control	22	0.000		x	x	x	x		x					
Cognitive load control	48	0.000		x	x	x	x	x	x	x	x	x	x	x
Stringent control	25	0.000		x			x	x						

Abbreviations: R: right; L: left; SMA: supplementary motor area; MFG: middle frontal gyrus; IFG: inferior frontal gyrus; INS: insula; IPC: inferior parietal cortex; DST: Dorsal striatum; CBL: cerebellum; cFWE: family-wise error correction at cluster level

Within the broad timing network, the pre-SMA and left anterior insula were the two regions with the most robust pattern of significant convergence across most, though not all, circumstances. In addition, both of these regions were resistant to the most stringent experimental controls, providing additional support for their fundamental role in duration processing. The (pre-)SMA and insula represent key structures in motor control and interoception, respectively. Activation of these two regions can therefore be interpreted in terms of embodied models of cognition that highlight the significance of both bodily states and simulation-like processes in the neural representation of cognitive processes (Barsalou, 2007). Indeed, several lines of research have proposed that the representation of duration might be embodied either via the sensorimotor (Addyman et al., 2017; J. Coull, Vidal, & Burle, 2016; Fernandes & Garcia-Marques, 2019; Hugo Merchant & Yarrow, 2016; Schubotz, 2007) or interoceptive (Craig, 2009; Fernandes & Garcia-Marques, 2019; Wittmann, 2009) systems.

In line with the central postulation of embodied theories of cognition regarding reenactment of sensory and motor representations by higher-order cognitive processes, the sensorimotor theory of timing posits that an abstract representation of duration is rooted in the motor system’s capacity for executing precisely timed coordinated motor programs (Balasubramaniam et al., 2021; Coull & Droit-Volet, 2018; Fernandes & Garcia-Marques, 2019; Merchant & Yarrow, 2016; Patel & Iversen, 2014). It has also been suggested that the pre-SMA, which is involved in action inhibition, might have become co-opted to represent time because it is recruited whenever a voluntary action has to be delayed, which is essentially the inhibition of a response in the temporal dimension (Coull et al., 2016; Kononowicz & van Rijn, 2015; Merchant & Yarrow, 2016). Furthermore, the motor system’s capacity for internal forward modeling, which relies on the sensorimotor representation of action established through unsupervised learning processes, might provide the support for our ability to make temporal predictions (Balasubramaniam et al., 2021; Schubotz, 2007). The (pre-)SMA, which plays a key role in the coordination and integration of action, may serve as the starting point of a simulation process by providing the input (the so-called corollary discharge or efference copy) to the forward model (Schubotz, 2007) or, alternatively, activity of its modality-independent neuronal subpopulations might provide top-down predictive signals to sensory and association areas (Merchant & Yarrow, 2016).

According to the interoceptive notion of temporal processing, our experience of time is intimately connected with the signaling of bodily states and related emotions (Wittmann, 2009). Our results reveal an almost ubiquitous contribution of the insula in duration processing (Table 1), and so are consistent with Craig’s proposed model of timing (2009) that posits the insula as a neural substrate for awareness across time. According to this model, the integration of primary interoceptive signals with the salient features of the sensory environment, and then with the motivational, hedonic, social, and cognitive inputs in the insula, along a posterior-to-mid-to-anterior axis, forms the basis for a unified meta-representation of the sentient self at the immediate moment of time. The serial accumulation of these endogenous time units is hypothesized to provide a potential basis for the subjective experience of time. In a recent study, Fernandes and Garcia-Marques (2019) recorded electromyographic (EMG) signals from two facial muscles during a temporal judgment task and found correlations between subjective duration and EMG gradients of the corrugator-supercilii muscles. The authors speculated that the accumulative proprioceptive-kinesthetic signals elicited from this muscle served as a source of internal bodily feeling that led to a sense of self-awareness over time. In addition, they proposed that this spontaneous motor output reflected the contribution of motor control structures in perceptual temporal processing. In addition, neuroanatomical evidence representing insular cortex as a converging point, having functional and structural connections with the (pre-)SMA (Deen et al., 2011) and IFG (Cai et al., 2014; Cerliani et al., 2012; Deen et al., 2011), and receiving proprioceptive-kinesthetic afferents from the peripheral nervous system, further illustrates that the classic interoceptive notion of temporal cognition could be expanded to include motor feelings associated with sensorimotor experiences more explicitly.

Also in accordance with the framework of embodied cognition, it has been suggested that temporal information in different timescales is processed by systems consistently implicated in behavioral functions operating within that time range (Buhusi & Meck, 2005; Murai & Yotsumoto, 2016). Our meta-analysis revealed that processing of short durations (< 500ms), commonly needed for motor coordination and speech generation, is primarily mediated by the DST, a key component of the motor system (Lewis & Miall, 2003a). By contrast, we found that processing of longer durations of up to a few seconds, integral for behaviors like foraging and decision-making, was mediated by higher-level cognitive systems, including the right-lateralized prefrontal and parietal cortical structures that subserve attention and working memory. It should be noted that in spite of the substantial dissociation between the neural substrates of short and long durations, imaging (Murai & Yotsumoto, 2016) and psychophysical (Lewis & Miall, 2009) findings support the idea of a continuum around the peri-second range, where both sets of structures would be recruited. Our results from the medium-range duration stimuli confirm this hypothesis.

### Embodied Timing as a Function of Sensory Embedding

Time as an entity, perceived through the dynamics of the internal and external world, does not rely on one particular sensory modality. A common view is that the duration of visual events will be coded in the visual cortex, or auditory events in the auditory cortex, before this information is then transferred to higher-order processing regions as an abstract amodal representation (Coull & Droit-Volet, 2018; Kononowicz & van Rijn, 2014; Merchant et al., 2013; Protopapa et al., 2019). Similarly, an embodied notion of time implies that intrinsic representation relies on simulations of temporal processing from everyday experience, which would entail representations being embedded within the specific sensory modality within which they were experienced. Indeed, a central principle of embodiment theories pertains to the reenactment of primitive perceptual, motor, or interoceptive states as a brain mechanism for representation and modulation of abstract information (Barsalou, 2007; Niedenthal, Barsalou, Winkielman, Krauth-Gruber, & Ric, 2005). In this respect, the processing of temporal information in different modalities would exploit modality-specific systems recruited by simulations of consolidated experiences of time in those modalities. Although we did not find modality-specific activity in low-level sensory regions due to the subtractive approach of most experiments (which would subtract out sensory activations common to both the timing and control tasks), a modality-specific system is not necessarily confined to sensory structures. The modality-specific nature of timing-related activations might instead manifest itself in distinct networks of higher-order regions (parietal, motor, or frontal cortices), depending on the different ways in which visual or auditory information is usually experienced. In the current paper, one of our novel tenets is that depending on the sensory system within which the temporal task is embedded, the internalized model of the events’ temporal characteristics will be underpinned by circuits specialized for either locomotive or gestural motor control (Todd, 1999). The gestural form can be described as a single continuous movement, while the locomotive form is associated with sequences of discrete movements arranged in a metrical structure.

Adhering to an embodied framework, we discuss our results according to stereotypical combinations of stimulus characteristics in everyday life, where auditory temporal information is typically sequential, and visual temporal information is often continuous. Indeed, it is easier to synchronize to an auditory input if it comprises a sequence of discrete sounds, but to visual stimuli if they are unitary moving trajectories (Hove et al., 2013; Silva & Castro, 2016). In the auditory–sequential domain, speech and music represent two natural sources of stereotypical temporal information that, due to their rich temporal dynamics and ecological significance, play a pivotal role in shaping cognition. These sequential auditory events probably rely on locomotor-like internal models, specialized for language processing. Similarly, there is evidence that the temporal processing of continuous visual events is supported by the mechanisms governing visuomotor processing (Ayhan & Ozbagci, 2020; Gavazzi et al., 2013; Orgs, Kirsch, & Haggard, 2013). This dichotomy is further illustrated by common combinations of stimulus structure and modality in the papers included in our meta-analysis, which resulted in a substantial overlap between the experiments included in the stimulus modality and contiguity classes and the resultant findings of these analyses (Fig. 3b and c). In experimental terms, while the visual modality is more often utilized to investigate the temporal processing of single interval stimuli (e.g., in duration discrimination), the study of beat-based timing is usually conducted on auditory stimuli (e.g., in rhythm monitoring tasks). We, therefore, discuss our results in terms of two broader groups of auditory beat-based and visual interval-based timing.

#### Timing of Auditory and Sequential Stimuli

The auditory and sequential analyses were found to yield similar patterns of convergent activation in the insula, IFG and adjacent ventrolateral PMC, DST, and SMA (including both the pre-SMA and SMA-proper). Humans’ superior timing capacity in the auditory modality is thought to originate from the privileged link between the auditory system and motor control regions, namely the (pre-)SMA, PMCv, posterior IFG, and DST (Comstock et al., 2018; Jancke, Loose, Lutz, Specht, & Shah, 2000; H. Merchant & Honing, 2013; Patel, Iversen, Chen, & Repp, 2005). Strong auditory-motor coupling results in enhanced entrainment of motor neurons to the dynamics of sensory activation and, subsequently, more stable internal models of temporal information (Balasubramaniam et al., 2021; Large & Snyder, 2009; Morillon & Schroeder, 2015; Proksch, Comstock, Médé, Pabst, & Balasubramaniam, 2020; Ross, Iversen, & Balasubramaniam, 2016). A stronger internal model produces a more accurate simulation of the temporal dynamics of the environment and subsequently more accurate top-down predictions and higher temporal performance. Following an embodied approach to time, the motor system establishes and tunes internal models through its active interaction with the environment. Although motor training of isolated actions can define task-relevant neural manifolds and enhance related sensorimotor representations, repetitive training on a sequence of recurring actions merges individual actions into a rhythmic pattern with greater dynamic stability (Balasubramaniam et al., 2021; Sakai, Hikosaka, & Nakamura, 2004; Zhang & Sternad, 2019). The most stable actions in the human motor repertoire, such as walking, dancing, and articulation, occur in a structured rhythmic sequence. Therefore, it is reasonable to conceive that internal models of the rhythmic structure of events are represented within motor substrates specialized for locomotive behaviors. Our findings concerning the selective activation of the DST and SMA-proper for auditory–sequential timing tasks provide support for this theoretical framework. Further support comes from the rehabilitation literature, where rhythmic auditory stimulation has proven to be an effective intervention program for behaviors of sequential nature, such as gait (for a review, see Pereira et al., 2019) and language (Fujii & Wan, 2014; Habib, 2021; Kotz & Gunter, 2015).

Language and music are two major sources of rhythmic auditory temporal information in our everyday life. It has been proposed that sensorimotor models of temporal structures “would call upon the vocal and articulatory system because rhythmic information is at the heart of vocal and articulatory production” (Schubotz, 2007). Behavioral evidence for the proposed relationship between rhythm processing, articulation and locomotion comes from the timescale that is mutually shared by the most accurately perceived and synchronized beats, the syllable rate of speech, and the comfortable walking pace (Daikoku et al., 2020; MacDougall & Moore, 2005; Rajendran, Teki, & Schnupp, 2018; Todd, Cousins, & Lee, 2007; Zalta, Petkoski, & Morillon, 2020). The abundance of clinical findings demonstrating temporal deficits in language-related disorders (for a review, see Ladányi, Persici, Fiveash, Tillmann, & Gordon, 2020; Habib, 2021), such as dyslexia (e.g., Boll-Avetisyan, Bhatara, & Höhle, 2020), specific language impairment (e.g., Cumming et al., 2015), and aphasia (e.g., Stefaniak, Lambon Ralph, De Dios Perez, Griffiths, & Grube, 2021) also support the underlying significance of language for timing. The ventrolateral PMC (PMCv), along with its anterior neighbor, the opercular region of IFG, is a central component of the articulatory system, controlling the “highly overlearned, frequently used, and flexibly recombinable articulatory and manual sequences” (Fiebach & Schubotz, 2006). Therefore, it is conceivable that ventrolateral PMC, with its role in audio-vocal transformation, is involved in the motor representation of the temporal structure of events (Chen et al., 2009; Fiebach & Schubotz, 2006; Zatorre, Chen, & Penhune, 2007). While our findings concerning the right-lateralized activation of the PMCv stands at odds with the left-lateralization of audio-vocal transformations in the language literature, it is consistent with the frequently documented activation of this region in the music literature (Brown et al., 2006; Cheung, Meyer, Friederici, & Koelsch, 2018; Musso et al., 2015).

The posterior IFG (Broca’s area) is associated with the construction of more complex linguistic and musical structures from their subordinate building blocks (Brown et al., 2006; Cheung et al., 2018; Flinker et al., 2015; Friederici, 2006). The contribution of posterior IFG to the construction of higher-order temporal sequences is demonstrated to occur beyond the linguistic and musical domains (Asano & Boeckx, 2015; Fadiga, Craighero, & D’Ausilio, 2009; Fiebach & Schubotz, 2006; Fitch & Martins, 2014; Jeon, 2014; Musso et al., 2015). Various sources of evidence (Clerget et al., 2012; Koechlin & Jubault, 2006; Stout, Toth, Schick, & Chaminade, 2008; Uddén, Ingvar, Hagoort, & Petersson, 2017; Wang et al., 2019) suggest the IFG is a crucial component of the shared substrates (Patel, 2011) for the processing of hierarchical structures in general. Therefore, we conclude that the superior portion of the right IFG cluster, specifically associated with the sequence stimuli, may be related to the construction of temporal sequences from individual intervals. According to a recent proposal by Asano (2021), the ability to build a domain-general hierarchical structure depends upon the hierarchical control of goal-directed action (Badre & Nee, 2018) and hierarchical internal models (Fiebach & Schubotz, 2006; Wolpert, Doya, & Kawato, 2003) that rely, respectively, on the cortico-basal ganglia-thalamocortical systems (CBGT) and the dorsal stream. Interestingly, the proposed circuitry for the hierarchical control of goal-directed action and of hierarchical internal models corresponds to the neural substrates of humans’ advanced beat processing capacity, advocated by the Gradual Audiomotor Evolution (GAE; H. Merchant & Honing, 2013) and Action Simulation for Auditory Prediction (Patel & Iversen, 2014) hypotheses, respectively.

The nuclei in the dorsal striatum form another key component of the motor system. They have been implicated in the processing of concatenated sequences in both beat-based timing (Grahn, 2009; Grahn & Rowe, 2009; H. Merchant, Grahn, Trainor, Rohrmeier, & Fitch, 2015; Teki, Grube, Kumar, & Griffiths, 2011) and chunking (Dahms et al., 2020; Graybiel, 1998; Wymbs, Bassett, Mucha, Porter, & Grafton, 2012). Processing of linguistic, musical, and motor sequences (Gobet et al., 2001) is made more efficient by automatic chunking, which has been demonstrated to be a powerful perceptual mechanism for overcoming resource limitations. Importantly, there is evidence that temporal structure contributes to the definition of chunk boundaries (Dowling, 1973; Gilbert, Boucher, & Jemel, 2015; Snyder, 2008). In addition, the internal coherence of locomotive behaviors, provided by either chunked (Li et al., 2013; Miller, 1956) or metrical (Essens & Povel, 1985; Geiser, Notter, & Gabrieli, 2012; Teki et al., 2011) structure, is found to benefit representational stability of dependent items. It is therefore conceivable that comparable mechanisms may be implicated in binding individual items together in both chunked and metrical structures. This notion of DST’s function is consistent with the GAE (Merchant & Honing, 2013) and the most recent account of the ASAP (Cannon & Patel, 2021) hypotheses. The interplay between the SMA-proper and dorsal striatum is proposed to provide the neural basis for the representation of beat-based rhythms, such that firing rate dynamics of SMA neuronal populations encode the beat interval, and the DST selects the subpopulation appropriate for encoding the next interval (Cannon & Patel, 2021). In other words, this model hypothesizes the DST as a sequencing component that chunks the learned succession of intervals into a rhythmic structure. In support of this proposal, our findings, presented in supplementary Tables 7, 10, 12, and 15, identified sequential stimuli as the major contributing factor to the convergent activation of the DST across analyses of auditory stimuli, and of perceptual, quantification, and stringently controlled tasks. Furthermore, the functional collaboration of the SMA-proper and DST is supported by significant convergence in SMA-proper voxels in precisely the same analyses that yielded convergent DST activation. The SMA-proper involvement in auditory beat-based timing supports the functional dissociation of the pre-SMA and SMA-proper (Coull et al., 2016; Schwartze et al., 2012) along a rostrocaudal axis of automaticity, with higher degrees of automaticity being localized more caudally. Taken together with the privileged access of the auditory system to the motor cortico-basal ganglia-thalamocortical (mCBGT) circuit (Merchant & Honing, 2013), our results underscore the facilitatory significance of the DST for beat-based timing. With regard to the considerable contribution of this region to the beat-based processing of time, we come to the conclusion that the DST, as a key substrate for associative learning (Liljeholm & O’Doherty, 2012; Penhune & Steele, 2012), probably provides a subcortical bypass for more straightforward representation of chains and hierarchies of learned interval sequences in closed-loop circuits.

By contrast to the DST that mediates relative or beat-based timing of sequences of learned intervals, the cerebellum is found to be more activated to undiscovered or weakly regular rhythmic structures (Lutz et al., 2000; Sakai et al., 1999; Teki et al., 2011), or in the early stages of learning when absolute intervals play a more principal role (Jouen et al., 2013; R. M. Spencer, Zelaznik, Diedrichsen, & Ivry, 2003; Teki et al., 2011). Indeed, the cerebellum is known for its involvement in the feedback-based formation and fine-tuning of the internal model of events (Dahms et al., 2020; Ishikawa, Tomatsu, Izawa, & Kakei, 2016; Penhune & Steele, 2012; Shadmehr & Krakauer, 2008). Its activation by sequential stimulus presentation, particularly in motor timing tasks, is consistent with its role in forward modelling and indicates a role in the adaptive adjustment of behavior based on the temporal correspondence between sensory input and motor output.

#### Timing of Visual and Single Interval Stimuli

The traditional notion of a general auditory advantage in temporal processing has been challenged by findings demonstrating that tapping to optimized moving visual stimuli, such as bouncing balls with realistic kinematics, is almost as good as tapping to auditory metronomes (Gan et al., 2015; Gu, Huang, & Wu, 2020; Hove et al., 2013; Silva & Castro, 2016). Temporal performance is facilitated not only by the visuospatial characteristics of the stimulus but also by its sensorimotor compatibility with the human motor repertoire (Allingham et al., 2021; Gavazzi et al., 2013). Visual interval timing might therefore be rooted in visuomotor gestural behaviors, just as auditory beat-based timing relies on motor control structures implicated in audiomotor locomotive behaviors, such as speaking. This conjecture complies with findings demonstrating that internal models of gestural movements, such as manual reach, help mediate the perception of visual intervals (Addyman et al., 2017), and that greater congruence between an individual’s internal model of action and visual kinematics leads to better timing performance (Gavazzi et al., 2013). Therefore, just as in the auditory modality, temporal processing in the visual modality relies on sensorimotor transformation of real-world experience. Accordingly, our analysis yielded clusters of convergent activation in ventrolateral PMC, adjacent IFC, and pre-SMA for the timing of visual stimuli, which substantially overlapped with the results of the auditory analysis.

By contrast, we found selective activation of right-lateralized IPL and middle frontal gyrus (MFG) for visual, but not auditory, stimuli. Moreover, this activation pattern was found for both visual and single interval timing tasks. Cytoarchitectonically, the identified MFG cluster primarily corresponds to the anterior superior portion of area 45b and an inferior posterior portion of area 46. Studies in non-human primates have demonstrated that area 45b receives projections from the visual association cortex (Frey et al., 2014). Area 45b is also connected to both the frontal eye field (FEF) and supplementary eye field (SEF), and is therefore affiliated with the frontal oculomotor system (Gerbella et al., 2007, 2010). In tandem with the caudal portion of area 46vc (possibly corresponding to the identified portion of the BA 46 in our study), this frontal network cooperates with the inferior parietal cortex to orient feature-based spatial attention and take part in oculomotor control (Borra & Luppino, 2019; Gerbella et al., 2010; Gerbella, Borra, Tonelli, Rozzi, & Luppino, 2013; Premereur, Janssen, & Vanduffel, 2015). The differential involvement of the right MFG-IPL network for visual stimuli and the DST for auditory stimuli reveals distinct control mechanisms for modality-specific timing. While auditory beat-based timing relies on subcortical structures that subserve automatic repetitive motor processes like walking (Hausdorff et al., 1998), visual interval-based timing engages the frontoparietal attention network (Lewis & Miall, 2003b) that is involved in gaze and manual control (Battaglia-Mayer et al., 2001; Hadjidimitrakis, Bakola, Wong, & Hagan, 2019). The greater reliance of visual timing on cognitive resources is compatible with the nature of gestural visuomotor processes. In contrast to the internal models of locomotive movements that, once established, remain relatively constant, internal models of visually guided movements, such as manual reach, depend on sustained attention to visual feedback (Buneo & Andersen, 2006; Zhao & Warren, 2015). Furthermore, the recruitment of the right MFG–IPL network in the relatively demanding processing of visual timing is consistent with previous work (Lewis & Miall, 2006; Joaquim Radua, Pozo, Gómez, Guillen-Grima, & Ortuño, 2014; Rubia & Smith, 2004), indicating shared neural structures between time perception and executive functions. Hence, by contrast to the auditory modality, the lower reliance of visual timing on motor substrates could be taken to indicate lower degrees of embodiment in visual temporal processing and a greater need for cognitive control.

### A Quantification vs. Prediction Dissociation of Timing

Our results from the quantification and prediction analyses indicate that the striatal or right-lateralized fronto-parietal activations often reported in studies of duration estimation are observed only when the duration has to be explicitly evaluated and quantified (e.g., pressing a button for a prespecified time or making a shorter/longer judgment). In addition, a comparison between the analyses of the two task goals yields a lateralization pattern with respect to IPL, where the left- and right-sided clusters correspond respectively to the prediction and quantification of time. The observed dissociation between the left and right IPL clusters is particularly consistent with brain stimulation findings demonstrating the causal significance of left IPL in temporal prediction of rhythms (Ross et al., 2018) and of right IPL in the temporal quantification of duration in discrimination tasks (Bueti et al., 2008).

This IPL lateralization pattern is also consistent with what we obtained from the stimulus contiguity analyses. Similar to the prediction and quantification analyses, the sequence and single interval analyses yielded left- and right-lateralized parietal activation, respectively. Learned rhythmic sequences induce predictions about the onset of an upcoming stimulus. Forming a temporal prediction about a forthcoming event in an ongoing sequence relies on the internal model of the sequence (Schubotz, 2007). An internal model is a sensorimotor representation of the events’ structure. The left IPL, as an interface receiving both feedforward motor and feedback sensory signals, is known to be recruited for linking sensory and motor systems through the dorsal stream (Rauschecker, 2011, 2018; Warren, Wise, & Warren, 2005). The dorsal stream is implicated in time-efficient sensory-motor integration, a phenomenon underlying the capacity for real-time internal representation of events’ structure (Bornkessel-Schlesewsky & Schlesewsky, 2013; Rauschecker, 2011, 2018) and predictive coding (Friston & Kiebel, 2009; Proksch et al., 2020; Zatorre et al., 2007). Its role in the transmission of top-down predictive signals to the sensory system, possibly through the optimized allocation of attentional resources to the anticipated moment in time (Bolger et al., 2014; Coull & Nobre, 1998; Morillon & Baillet, 2017), results in ‘Active Sensing’ and facilitates the processing of upcoming stimulus (Morillon & Schroeder, 2015; Schroeder, Wilson, Radman, Scharfman, & Lakatos, 2010). By contrast, the quantification of novel temporal information in single interval experiments would be substantially stimulus-driven. Accordingly, as discussed in Sect. 4.2.2, the right MFG–IPL network, obtained from the quantification analysis, could be representative of such stimulus-driven attention to duration. Hence, the observed dissimilarity in IPL lateralization for prediction versus quantification can be attributed to the dissociation between model-based and stimulus-driven processing of duration, respectively.

### Limitations and Future Directions

Although the ALE coordinate-based meta-analysis was originally developed to disentangle structural rather than functional ambiguities in the neuroimaging literature, the comparative application of this technique to groups of experiments organized on the basis of functional attributes narrows down the potential role of the identified regions. Accordingly, we postulate that brain structures identified to be exclusively connected with a categorical property should be functionally involved in the processing of that specific attribute. For instance, the exclusive contribution of sequential stimuli to the convergent activity in the dorsal striatum is indicative of its functional significance for beat-based timing. In contrast, the ALE analysis of functionally-separated studies do not allow to speculate on the possible functional roles of brain structures that respond quasi-ubiquitously across various contexts, such as the pre-SMA or insula.

Since the incorporated quality assessment tool was originally developed for clinical studies, its items do not perfectly match to our neuropsychological study. Therefore, the obtained score for each included study might not be the most relevant yardstick for assessing its quality. In addition to the quality of the included studies, their methodological heterogeneity due to differences in imaging techniques, scanner types, and statistical analyses may have influenced the results we obtained. However, we could not investigate those methodological impacts because the number of studies in some subcategories (e.g. PET studies) fell below the minimum number required to control the excessive contribution of individual experiments (Eickhoff et al., 2016). In addition, despite the conservative approach of the ALE technique and our strict compliance with CBMA meta-analysis guidelines (Müller et al., 2018; Tahmasian et al., 2019), the question of whether the obtained results depend on the meta-analysis technique we used requires future studies to compare our results with that of other available CBMA tecniques, such as (ES-)SDM (Radua et al., 2012), KDA (Wager et al., 2007), and GPR (Salimi-Khorshidi et al., 2009).

The uneven distribution of experiments with other stimulus or task characteristics among classes of a single categorical dimension is a major issue for comparison between the analyses and any consequent functional inferences. For instance, a direct comparison between the perceptual and motor analyses yielded convergent activation in the DST that could have been interpreted as a significant role for the DST in perceptual, but not motor, timing processes. However, a closer look at the contribution information in supplementary Table 10 reveals a predominant contribution of sequential stimuli from rhythm perception tasks to the right DST cluster. On the other hand, a much lower (possibly inadequate) number of sequential stimuli from motor experiments were available for inclusion in the analysis, possibly explaining the preferential activation of DST by perceptual tasks. Another instance of disproportionate distribution of experiments comes from the duration range analyses. Since experiments with a medium-range (500–1500 ms) stimulus duration comprise the majority of timing experiments, it has considerable neuroanatomical overlap with the results of the all-effects analysis. By contrast, the relative sparsity of results obtained from the analyses of short (< 500 ms) and long (> 500 ms) duration range stimuli cannot conclusively be conceived to represent more focal neuroanatomical substrates but, rather, might index lower convergence simply due to the small number of included experiments. A similar conclusion can be drawn for the lack of cerebellar convergent activation in the single-interval analysis. Considering the modular organization of internal models in the cerebellum (Imamizu & Kawato, 2012; Imamizu, Kuroda, Miyauchi, Yoshioka, & Kawato, 2003), the absence of this region could have resulted from task-dependent (rather than contiguity-related) spatial heterogeneity of the reported cerebellar data.

In addition to the auditory beat-based and visual interval-based timing, humans are, of course, also capable of processing temporal information within visual sequences and auditory single-intervals. However, we speculate that the brain mechanisms for processing these rarer stimulus combinations are not as efficient as their stereotypical counterparts that have higher ecological relevance. We also conjecture that these atypical combinations activate a mixture of the neural correlates of each stereotypical pair. We acknowledge it would be advantageous to narrow the potential functional significance of our neuroanatomical results by expanding the auditory–sequential and visual–single-interval segmentation into further dimensions, such as interval duration. While such further sub-divisions could provide a more precise picture of the brain correlates of temporal processing, the unbalanced distribution of experiments and the small sample size of the resulting subsections would, in practice, restrict further analyses.

Considering the above-mentioned methodological limitations, we cannot definitely argue that the embodiment framework provides the key to an ultimate “taxonomy of time” (Meck & Ivry, 2016; Paton & Buonomano, 2018), but it is worth testing if different timing tasks that share a combination of temporal characteristics just like their stereotypical groupings would engage common neural mechanisms and circuits. We hope that insights from the processing of time in real-life contexts will encourage researchers to progress towards timing experiments with higher ecological validity that investigate how the interplay of multiple temporal characteristics affects neural substrates of timing.

### Conclusion

In this multi-dimensional meta-analysis, anatomical commonalities across categories reveal an almost ubiquitous activation of the pre-SMA and bilateral insula extending into the IFG. Anatomical specificities indicate that the auditory–sequential and visual–single interval stimuli, as the key segregating factors, recruit the DST–SMA-proper and right MFG-IPL networks, respectively. While activation of the pre-SMA and bilateral insula are proposed to be associated with the sensorimotor and interoceptive notions of embodied temporal cognition, our findings from the context-dependent activations are also consistent with an embodied framework according to which an abstract representation of time is grounded in the sensorimotor processes that subserve actions with a comparable temporal profile. In particular, the DST and SMA-proper are known to be recruited for the execution of stabilized locomotive behaviors, while the MFG–IPL network is proposed to be associated with gestural behaviors. Accordingly, we come to the conclusion that the embodied nature of temporal processes more strongly implies an intrinsic rather than a dedicated neural implementation of timing processes.

### Electronic Supplementary Material

Below is the link to the electronic supplementary material.

Supplementary Material 1

## Data Availability

The included studies and their classification information is available in the supplementary materials. A table of coordinated will be provided by request.
