# Supplementary material for: Embodying Time in the Brain: A Multi-Dimensional Neuroimaging Meta-Analysis of 95 Duration Processing Studies
Source: Neuropsychol Rev. 2023 Mar 1;34(1):277–98. doi: 10.1007/s11065-023-09588-1 (PMC10920454; doi:10.1007/s11065-023-09588-1)
Supplement: Supplementary file 1 — Supplementary Material 1 [file 11065_2023_9588_MOESM1_ESM.docx]

**Supplementary materials for Naghibi et al., Embodying time in the brain: A multi-dimensional neuroimaging meta-analysis of 95 duration processing studies. Neuropsychology Review**

Contents

[Appendix 1. Methods 4](#_Toc112057804)

[Search strategy and study selection 4](#_Toc112057805)

[Data classification 4](#_Toc112057806)

[Stimulus duration dimension 4](#_Toc112057807)

[Stimulus modality dimension 5](#_Toc112057808)

[Stimulus contiguity dimension 5](#_Toc112057809)

[Sensorimotor processing dimension 5](#_Toc112057810)

[Task goal dimension 5](#_Toc112057811)

[Control task stringency dimension 6](#_Toc112057812)

[Activation likelihood estimation (ALE) 7](#_Toc112057813)

[Appendix 2. Results 8](#_Toc112057814)

[Analyses based on stimulus duration 8](#_Toc112057815)

[Convergence across short duration experiments 8](#_Toc112057816)

[Convergence across medium duration experiments 8](#_Toc112057817)

[Convergence across long-duration experiments 8](#_Toc112057818)

[Common activations between classes of stimulus duration 9](#_Toc112057819)

[Analyses based on stimulus modality 9](#_Toc112057820)

[Convergence across visual modality experiments 9](#_Toc112057821)

[Convergence across auditory modality experiments 9](#_Toc112057822)

[Common activations between classes of stimulus modality 10](#_Toc112057823)

[Analyses based on stimulus contiguity 10](#_Toc112057824)

[Convergence across single-interval experiments 10](#_Toc112057825)

[Convergence across sequential experiments 10](#_Toc112057826)

[Common activations between classes of stimulus contiguity 10](#_Toc112057827)

[Analyses based on sensorimotor processing 11](#_Toc112057828)

[Convergence across perceptual experiments 11](#_Toc112057829)

[Convergence across motor experiments 11](#_Toc112057830)

[Common activations between perceptual and motor timing experiments 11](#_Toc112057831)

[Analyses based on task goal 12](#_Toc112057832)

[Convergence across quantification goal experiments 12](#_Toc112057833)

[Convergence across prediction goal experiments 12](#_Toc112057834)

[Common activations between classes of task goal 12](#_Toc112057835)

[Analyses based on the control task stringency 13](#_Toc112057836)

[Convergence across task control experiments 13](#_Toc112057837)

[Convergence across cognitive load control experiments 13](#_Toc112057838)

[Convergence across stringent control experiments 13](#_Toc112057839)

[Common activations between levels of task control 13](#_Toc112057840)

[Supplementary Table 1. Included Experiments 14](#_Toc112057841)

[Supplementary Table 2. Results of the All-effects Analysis. 20](#_Toc112057842)

[Supplementary Table 3. Results of the Short Duration Analysis 24](#_Toc112057843)

[Supplementary Table 4. Results of the Medium Duration Analysis 26](#_Toc112057844)

[Supplementary Table 5. Results of the Long Duration Analysis 30](#_Toc112057845)

[Supplementary Table 10. Results of the Perceptual Tasks Analysis 45](#_Toc112057846)

[Supplementary Table 13. Results of the Prediction Tasks Analysis 55](#_Toc112057847)

[Supplementary Table 17. PRISMA (2020) checklist 66](#_Toc112057848)

[Supplementary Table 18. PRISMA (2020) abstract checklist 72](#_Toc112057849)

[Supplementary Table 19. Quality assessment score checklist 74](#_Toc112057850)

[Supplementary references 75](#_Toc112057851)

# Appendix 1. Methods

## Search strategy and study selection

We used the following detailed keyword string ("time perception" OR "time prediction" OR "time estimation" OR "time discrimination" Or "time production" OR "time reproduction" OR "time dilation" OR "temporal perception" OR "temporal prediction" OR "temporal estimation" OR "temporal discrimination" OR "temporal production" OR "temporal reproduction" OR "temporal processing" OR "duration prediction" OR "duration discrimination" OR "interval timing" OR "motor timing" OR "perceptual timing" OR "interval estimation" OR "interval production" OR "temporal order" OR "temporal expectation" OR "temporal preparation" OR "paced finger-tapping "OR "rhythm perception" OR "rhythm production" OR "rhythm reproduction") AND ( fMRI OR "functional magnetic resonance imaging" OR "positron emission tomography" OR PET).

## Data classification

### Stimulus duration dimension

In contrast to prior meta-analyses that dichotomized studies according to a rather arbitrary and non-biological one-second boundary (Lewis & Miall, 2003; Nani et al., 2019; Schwartze, Rothermich, & Kotz, 2012; M. Wiener, P. Turkeltaub, & H. B. Coslett, 2010a), we aimed to choose our duration boundaries as close as possible to the highly automatic and highly controlled extremes of the available duration span, so as to be able to isolate brain areas more specifically contributing to the processing of short and long durations. In order to do so, we chose our duration boundaries so marginally that only a minimum number of experiments being allocated to the short and long duration classes. This strategy resulted in the choice of 500 and 1500 ms for the short-medium and medium-long boundaries, respectively. This choice of duration boundaries also corresponds to the experimental data, suggesting different timing mechanisms come into play at around the same durations. Accordingly, the boundary between automatic and cognitively controlled timing mechanisms is proposed to lie somewhere around 500 ms (Dean V. Buonomano, Jennifer Bramen, & Mahsa Khodadadifar, 2009; Spencer, Karmarkar, & Ivry, 2009), where shorter durations probably rely on more automatic, intrinsic timing circuits. In addition, durations longer than 1500 ms are proposed to be more likely to induce verbal counting strategies (Grondin, Ouellet, & Roussel, 2004).

### Stimulus modality dimension

While possibly due to the centralized view of temporal processing, it is unusual to incorporate the stimulus modality as a dissociating factor, we decided to investigate the neural substrates engaged in modality-specific processing of temporal information. Major distinctions between the neural correlates of timing in each modality can provide evidence in favor of the context-dependent notion of temporal processes.

### Stimulus contiguity dimension

It has been proposed that beat-based and interval-based timing are mediated by partially different neurocognitive mechanisms (Grube, Cooper, Chinnery, & Griffiths, 2010; Merchant & Honing, 2013; Teki, Grube, Kumar, & Griffiths, 2011). In order to investigate the neuroanatomical correlates of the proposed dissociation, we conducted separate ALE analyses on experiments with single-interval and sequential stimuli. This categorical division largely overlaps with Lewis and Miall's (2003) discrete/continuous category, Schwartze et al.'s (2012) non-sequential/sequential category, and Teghil et al.'s (2019) internal/external category.

### Sensorimotor processing dimension

This categorical division coincides with prior meta-analyses of perceptual/motor timing (Lewis & Miall, 2003; Nani et al., 2019; Schwartze et al., 2012; Wiener et al., 2010a).

### Task goal dimension

Duration processing studies have already been categorized according to the task goal (Schwartze et al., 2012; Wiener et al., 2010a; M. Wiener, P. E. Turkeltaub, & H. B. Coslett, 2010b). For instance, Wiener et al. (2010a; Wiener et al., 2010b) conducted two distinct meta-analyses of explicit and implicit timing tasks, categorizing studies as a function of whether overt estimates of duration had to be provided (explicit) or whether temporal information could be used to improve sensorimotor processing of non-temporal stimulus features (implicit). More recently, Teghill et al. (2019) categorized studies into internally-based and externally-cued timing, according to whether the duration being measured was defined by internal, self-determined criteria or by an external sensory stimulus. By contrast, we categorized studies in terms of whether temporal information was quantified (usually with a perceptual or motor judgment) or whether temporal information was used to predict when a stimulus would appear (which improved speed or accuracy of stimulus detection). Duration discrimination and rhythm reproduction are examples of quantification tasks, while temporal orienting and synchronization are examples of prediction tasks. There is a considerable degree of overlap between our quantification/prediction categories and Wiener et al.'s explicit/implicit categories. There is also partial overlap between our categories and Teghill et al.'s (2019) internally-based/externally-cued categories, although there are also significant differences. For example, Teghill et al. (2019) categorized the synchronization-continuation task (SCT) as an externally-cued timing task, but we class the synchronization phase of the SCT as a prediction task and the continuation phase of the SCT as a quantification task. In the synchronization phase, participants have to predict when the next (external) stimulus will occur so as to synchronize their responses to stimulus onset, but in the continuation phase, they must rely on an internal representation of duration in order to reproduce the correct inter-stimulus interval. In addition, Teghill et al. (2019) categorized temporal orienting as an internally-based timing task, but we categorize it as a prediction task because participants must predict when an external stimulus will appear so as to process its sensory features as quickly or as accurately as possible.

### Control task stringency dimension

Duration processing is a multi-layered process, and even studies that include active control tasks are successful in controlling for non-temporal processes to a greater or lesser extent. The majority of studies successfully controlled for the basic sensorimotor, mnemonic, attentional, and decisional demands of the timing task. However, these measures are not sufficient for controlling for the cognitive demands of processing the duration of the stimulus itself. Estimating stimulus duration, at least for durations more than 300ms or so (D. V. Buonomano, J. Bramen, & M. Khodadadifar, 2009; Spencer et al., 2009), requires that the initial moment of stimulus onset be held in working memory (WM), for attention to be maintained on the stimulus throughout its entire presentation, and for the contents of WM to be continually updated as a function of elapsing time. By contrast, such dynamic processing would not be required for estimating a control stimulus feature such as color or pitch. Therefore, we categorized studies according to whether the control task simply had the same basic sensorimotor and cognitive features as the timing task (task control) or whether the control task also matched in terms of either stimulus dynamics or task difficulty (cognitive load control) or equated with the timing task in all the above criteria (stringent control).

## Activation likelihood estimation (ALE)

This analysis relies on models of spatial uncertainty of reported peak activation coordinates, attributable to between-subject and between-laboratory variability of neuroimaging data (Eickhoff et al., 2009). In this regard, each focus is modeled by a 3D Gaussian probability distribution, whose width, as a measure of spatial uncertainty, decreases with an increase in the number of participants. Then, ALE scores are computed by the voxel-wise union of modeled activation maps, which are obtained by combining the probability distribution of all foci reported in a given experiment. Finally, these ALE scores are assessed against a null distribution, reflecting a random spatial association between the modeled activation maps of independent experiments. The earlier versions of ALE used the random distribution of foci instead of experiments to obtain a null distribution. In the recent version, the assumption of fixed within-experiment and random between-experiment association of foci invokes a random-effects analysis, allowing conclusions to be drawn about a wider set of related experiments than those included in the meta-analysis (Eickhoff et al., 2009).

# Appendix 2. Results

## Analyses based on stimulus duration

The three analyses conducted on short, medium, and long classes of the duration dimension included 19, 58, and 19 experiments, and 311, 901, and 280 participants, respectively.

### Convergence across short duration experiments

The analysis conducted on experiments with a short-range duration identified regions of significant convergence in two clusters located in the left insula extending toward putamen, and the right putamen (fig. 3a; supplementary table 3)

### Convergence across medium duration experiments

The analysis conducted on experiments with a medium-range duration yielded nine clusters, including the pre-SMA extending toward the SMA-proper and PCG, right MFG (BA 45, 46), bilateral insula extending toward the opercular IFG and PMCv, bilateral DST, bilateral IPS extending toward right IPL, and left cerebellum crus I extending toward cerebellum Crus II and VI (fig. 3a; supplementary table 4).

### Convergence across long-duration experiments

Testing for significant convergence across experiments with long durations yielded five clusters located in the pre-SMA, PCG, bilateral frontal operculum cortex, extending toward right IFG and PMCv, and right IPL (fig. 3a; supplementary table 5).

### Common activations between classes of stimulus duration

The three classes of stimulus duration had merely a small locus of common activation in the left insular cortex. However, we also found common activations in the right putamen between the medium and short durations; and four more loci in the pre-SMA, PCG, right insula, and right IFG and PMCv between the medium and long durations.

## Analyses based on stimulus modality

As mentioned earlier, the number of experiments with tactile sensory stimulation was far below the minimum threshold for conducting an ALE analysis. The two remaining classes of stimulus modality, namely the visual and auditory classes, comprise 57 and 32 experiments and 858 and 548 participants, respectively.

### Convergence across visual modality experiments

For the visual experiments, we found convergent activations in the pre-SMA, right MFG (BA46 and BA45 ), bilateral insula extending toward the bilateral opercular IFG and right PMCv, and right IPL (fig. 3b; supplementary table 6).

### Convergence across auditory modality experiments

Testing for significant convergence across the experiments with auditory stimuli resulted in five clusters, comprising the pre-SMA extending toward the PCG, SMA-proper, right opercular IFG extending toward the PMCv, right putamen, and left pallidum extending toward ipsilateral putamen, insula, and frontal operculum (fig. 3b; supplementary table 7).

### Common activations between classes of stimulus modality

The analyses on visual and auditory stimuli found areas of common activation in the pre-SMA extending toward PCG, left insula, and right opercular IFG and PMC.

## Analyses based on stimulus contiguity

The two classes of single-interval and sequence stimuli comprised 46 and 39 experiments and 707 and 625 participants, respectively. Due to the insufficient number of included experiments, we decided not to report the results of the trajectory analysis.

### Convergence across single-interval experiments

Across experiments with single stimuli, convergent activation was found in the pre-SMA extending toward the PCG, right MFG (area 45, 46), bilateral insula extending toward the IFG, and right IPL (fig. 3c; supplementary table 8).

### Convergence across sequential experiments

Regions of high concordance among experiments with sequential stimuli were found in eight clusters located in the pre-SMA extending toward the SMA-proper and PCG, bilateral insula extending toward the anterior superior temporal gyrus (STG), left IFG and PMCv, right opercular IFG extending toward PMCv, bilateral DST, left IPS, and left cerebellum crus I (fig. 3c; supplementary table 8).

### Common activations between classes of stimulus contiguity

Single and sequences stimuli were found to commonly activate the pre-SMA, bilateral insula, and IFG.

## Analyses based on sensorimotor processing

The following analyses disentangle the neural correlates of perceptual and motor timing tasks, each including 55 and 48 experiments, comprising 961 and 637 participants, respectively.

### Convergence across perceptual experiments

Among the perceptual timing experiments, convergent activations were identified in four clusters, including the pre-SMA extending toward the SMA-proper and PCG, the bilateral insular cortex extending toward the opercular IFG, PMCv, and left DST, and right DST (fig. 4a; supplementary table 11).

### Convergence across motor experiments

The motor timing experiments converged in six clusters, including the pre-SMA extending toward the PCG, right MFG, bilateral insula extending toward the IFG and right PMCv, and anterior portion of STG, left IPS, and left cerebellum curs I, extending toward cerebellum crus II and VI (fig. 4a; supplementary table 11).

### Common activations between perceptual and motor timing experiments

Perceptual and motor timing experiments mutually activated the pre-SMA extending toward the PCG, bilateral insula, opercular IFG, and right PMCv.

## Analyses based on task goal

The results of the two classes of experiments with quantification and prediction task goals, respectively including 65 and 32 experiments, as well as 1027 and 448 participants, are as follows.

### Convergence across quantification goal experiments

The experiments whose task goal was to explicitly quantify duration activated a distributed network of cortical and subcortical brain structures, including the pre-SMA extending to the SMA-proper and PCG, right insula extending toward IFG, PMCv, and MFG, left IFG extending toward insula, PMCv, and DST, right DST, right IPS extending into IPL, and the left cerebellum crus I and II (fig. 4b; supplementary table 12).

### Convergence across prediction goal experiments

Among the experiments whose task goal was to use temporal information to help predict the onset of upcoming stimuli, convergent activation was detected in four clusters, including the pre-SMA extending toward the PCG, bilateral insular cortex, and left IPS (fig. 4b; supplementary table 13).

### Common activations between classes of task goal

We found areas of common activation for quantification and prediction task goals in the pre-SMA, PCG, and bilateral insula.

## Analyses based on the control task stringency

With respect to the stringency of control for non-temporal processes, analyses were subdivided into a task control class including 22 experiments and 332 participants, a cognitive load control class including 48 papers and 732 participants, and a stringent control class with 25 experiments and 399 participants.

### Convergence across task control experiments

Testing for significant convergence across experiments with the task level of control, five clusters were detected in the pre-SMA extending to the PCG, right MFG, bilateral IFG pars opercularis extending toward right PMCv, and right insula (fig. 4c; supplementary table 14).

### Convergence across cognitive load control experiments

The analysis of cognitive load-controlled experiments resulted in a distributed network of nine clusters, including the pre-SMA/PCG, right MFG, bilateral insula extending toward the opercular IFG and right PMCv, bilateral DST, bilateral IPS extending into the right IPL, and left cerebellum crus I (fig. 4c; supplementary table 15).

### Convergence across stringent control experiments

The stringently controlled experiments were found to converge in two clusters, with maximum ALE scores in the pre-SMA and left insula extending toward the IFG (fig. 4c; supplementary table 16).

### Common activations between levels of task control

The three levels of control for non-timing processes were found to commonly activate a tiny cluster in the left IFG pars opercularis.

# Supplementary Table 1. Included Experiments

|  | **Authors** | **Number of participants (females)** | **Age of participants** | **Imaging modality** | **Timing task** | **Contrast** | **Stimulus duration** | **Sensorimotor processing** | **Stimulus contiguity** | **Task goal** | **Control task stringency** | **Sensory modality** | **Stereotactic space** | **Quality assessment score** |
| --- | --- | --- | --- | --- | --- | --- | --- | --- | --- | --- | --- | --- | --- | --- |
| 1 | [Apaydın et al., 2018](#OLE_LINK1) | 18 (11) | 25.8±5.8 | fMRI | Spatiotemporal judgment | Spatiotemporal judgment − Color tone judgment | Long | Perceptual | Trajectory | Predict & Evaluate | Cognitively controlled | Visual | MNI | 8 |
| 2 | [Araneda et al., 2016](#OLE_LINK2) | 27 (NA) | 26±7.02 | fMRI | Rhythm perception | Beat − No beat | Medium | Perceptual | Sequence | Evaluate | Stringently controlled | Auditory | TAL | 8.5 |
|  |  |  |  |  |  |  |  |  |  |  |  | Tactile |  |  |
|  |  |  |  |  |  |  |  |  |  |  |  | Visual |  |  |
| 3 | [Aso et al., 2010](#OLE_LINK3) | 14 (4) | 24-31 | fMRI | Duration discrimination | (Interval and Reproduction + Interval) − (Size and Reproduction + Size) | Medium | Perceptual | Single interval | Evaluate | Cognitively controlled | Visual | MNI | 8 |
|  |  |  |  |  | Reproduction | (Interval and Reproduction + Size and Reproduction) − (Interval + Size) |  | Motor |  |  |  |  |  |  |
| 4 | [Assmus et al., 2003](#OLE_LINK4) | 12 (0) | 25.6±3.7 | fMRI | Spatiotemporal prediction | Collision − Size | Long | Perceptual | Trajectory | Predict | Cognitively controlled | Visual | MNI | 8 |
| 5 | [Bengtsson et al., 2004](#OLE_LINK5) | 7 (2) | 18-25 | fMRI | Continuation | (Combined + Temporal) − (Ordinal + Even one key) | Medium | Motor | Sequence | Evaluate | Cognitively controlled | NA | TAL | 7 |
| 6 | [Bengtsson et al., 2005](#OLE_LINK6) | 7 (3) | 24.3±2.1 | fMRI | Continuation | Conjunction SEQ − ISO | Medium | Motor | Sequence | Evaluate | Stringently controlled | Auditory | MNI | 7 |
| 7 | [Bengtsson & Ullén, 2006](#OLE_LINK7) | 11 (0) | 33±NA | fMRI | Rhythm production | (Combined + Rhythm) − (Melody + ISO) | Medium | Motor | Sequence | Evaluate | Stringently controlled | Auditory | TAL | 8.5 |
| 8 | [Bengtsson et al., 2009](#OLE_LINK8) | 17 (3) | 23.6±NA | fMRI | Passive listening | Metric & Non-Metric − ISO | Short | Perceptual | Sequence | Evaluate | Stringently controlled | Auditory | TAL | 7.5 |
| 9 | [Beudel et al., 2009](#OLE_LINK9) | 18 (9) | 27±8.4 | fMRI | Spatiotemporal judgment | Time ahead − Place ahead | Long | Perceptual | Trajectory | Predict & Evaluate | Stringently controlled | Visual | MNI | 8.5 |
| 10 | [Billington et al., 2010](#OLE_LINK84) | 10 (3) | 20-40 | fMRI | Spatiotemporal prediction | Receding − random  Looming − random | Short | Motor | Trajectory | Predict | Cognitively controlled | Visual | TAL | 7.5 |
| 11 | [Bolger et al., 2014](#OLE_LINK94) | 17 (9) | 26.75±NA | fMRI | Orienting | Strong beat − Weak beat | Short | Motor | Sequence | Predict | Cognitively controlled | Auditory & visual | MNI | 8 |
| 12 | [Bueti & Macaluso, 2011](#Bueti2011) | 12 (9) | 26±5.8 | fMRI | Encoding | Encoding time – Control (auditory & visual) | Medium | Perceptual | Single interval | Evaluate | Stringently controlled | Auditory & visual | MNI | 8.5 |
|  |  |  |  |  | Reproduction | Temporal reproduction – Control (auditory & visual) |  | Motor |  |  |  |  |  |  |
| 13 | [Bueti et al., 2008](#Bueti2008) | 14 (10) | 25±5.2 | fMRI | Reproduction | Action − Control | Medium | Motor | Single interval | Evaluate | Sensorimotor controlled | Visual | MNI | 7.5 |
|  |  |  |  |  |  | Perception − Control |  |  |  |  |  |  |  |  |
| 14 | [Bueti et al., 2010](#OLE_LINK90) | 12 (4) | 25±5.2 | fMRI | Orienting | Covariation with the anticipation function | NA | Motor | Single interval | Predict | Stringently controlled | Visual | MNI | 8.5 |
| 15 | [Carlsson et al., 2006](#OLE_LINK12) | 9 (5) | 25±NA | fMRI | Orienting | Correlated − Uncorrelated | NA | Perceptual | Single interval | Predict | Cognitively controlled | Tactile | TAL | 6 |
| 16 | [Carvalho et al., 2016](#OLE_LINK13) | 16 (6) | 25.4±NA | fMRI | Spatiotemporal prediction | Periodic – Non-periodic | Long | Motor | Trajectory | Predict | Cognitively controlled | Visual | TAL | 8 |
| 17 | [Chen et al., 2008](#OLE_LINK14) | 12 (NA) | 23.83±NA | fMRI | Synchronization | Covariation with rhythm complexity (Non musicians) | Medium | Motor | Sequence | Predict | Cognitively controlled | Auditory | MNI | 7 |
|  |  | 12 (NA) | 23.17±NA | fMRI | Synchronization | Covariation with rhythm complexity (Musicians) | Medium | Motor | Sequence | Predict | Cognitively controlled | Auditory | MNI |  |
| 18 | [Cotti et al., 2011](#OLE_LINK15) | 14 (9) | 24±NA | fMRI | Orienting | Temporal – Neutral | Medium | Motor | Single interval | Predict | Cognitively controlled | Visual | MNI | 8 |
| 19 | [Coull et al., 2015](#OLE_LINK16) | 16 (1) | 26±NA | fMRI | Duration discrimination | Temporal – Spatial (dynamic trajectory) | Medium | Perceptual | Trajectory | Evaluate | Stringently controlled | Visual | MNI | 8.5 |
| 20 | [Coull et al., 2016](#OLE_LINK17) | 16 (NA) | 26.9±5.9 | fMRI | Orienting | Temporal – Neutral | Medium | Motor | Single interval | Predict | Cognitively controlled | Visual | MNI | 8 |
| 21 | [Coull et al., 2013](#OLE_LINK18) | 15 (NA) | 29.4±NA | fMRI | Reproduction | Temporal production – Self-paced movement | Medium | Motor | Single interval | Evaluate | Sensorimotor controlled | Visual | MNI | 7/5 |
|  |  |  |  |  | Orienting | Temporal prediction – simple RT task |  |  |  | Predict |  |  |  |  |
| 22 | [Coull et al., 2012](#OLE_LINK19) | 16 (1) | 25.7±NA | fMRI | Duration discrimination | Time – Color | Medium | Perceptual | Single interval | Evaluate | Stringently controlled | Visual | MNI | 9 |
| 23 | [Coull et al., 2008](#OLE_LINK20) | 14 (0) | 24±NA | fMRI | Duration discrimination | Time – Color | Medium | Perceptual | Single interval | Evaluate | Stringently controlled | Visual | MNI | 8.5 |
| 24 | [Coull & Nobre, 1998](#OLE_LINK21) | 7 (0) | 29±NA | PET | Orienting | Temporal orienting –  Spatial orienting | Medium | Motor | Single interval | Predict | Cognitively controlled | Visual | MNI | 7 |
|  |  | 8 (4) | 30±NA | fMRI | Orienting | Temporal orienting – Spatial orienting | Medium | Motor | Single interval | Predict | Cognitively controlled | Visual | MNI |  |
| 25 | [Coull et al., 2001](#OLE_LINK22) | 10 (5) | 25.1±NA | fMRI | Orienting | Temporal cue – Neutral cue | Medium | Motor | Single interval | Predict | Cognitively controlled | Visual | MNI | 7 |
| 26 | [Coull et al., 2008](#OLE_LINK23) | 12 (0) | 23.5±NA | fMRI | Spatiotemporal judgment | (TTCallo – COLallo) & (TTCego – COLego) | Medium | Perceptual | Trajectoty | Predict & Evaluate | Stringently controlled | Visual | MNI | 7.5 |
| 27 | [Coull et al, 2004](#Coull2004) | 12 (0) | 23.4±NA | fMRI | Duration discrimination | Covariation with attention to time relative to color | Medium | Perceptual | Single interval | Evaluate | Stringently controlled | Visual | MNI | 8 |
| 28 | [Cui et al., 2009](#Cui2009) | 20 (9) | 27.7±NA | fMRI | Orienting | Covariation with anticipation function | Long | Motor | Single interval | Predict | NA | Visual | MNI | 7 |
| 29 | [Davranche et al., 2011](#Darvanche2011) | 13 (3) | 30±8 | fMRI | Orienting | Motor detection – Neutral | Medium | Motor | Single interval | Predict | Cognitively controlled | Visual | MNI | 7 |
|  |  |  |  |  |  | Perceptual discrimination –Neutral |  | Perceptual |  |  |  |  |  |  |
| 30 | [Dormal et al., 2011](#Dormal2011) | 15 (0) | 21±2.3 | fMRI | Duration discrimination | Duration – Reference | Medium | Perceptual | Single interval | Evaluate | Sensorimotor controlled | Visual | MNI | 7.5 |
| 31 | [Ferrandez et al., 2003](#Fernandez2003) | 11 (6) | 24±3.5 | fMRI | Duration discrimination | Duration – Intensity | Medium | Perceptual | Single interval | Evaluate | Sensorimotor controlled | Visual | TAL | 7.5 |
| 32 | [Field & Wann, 2005](#OLE_LINK91) | 12 (NA) | NA | fMRI | Spatiotemporal prediction | TTC judgment – Inflation judgment | Short | Perceptual | Trajectory | Predict | Stringently controlled | Visual | TAL | 8 |
| 33 | [Gandour et al., 2002](#OLE_LINK29) | 10 (7) | 25.4±2.8 | fMRI | Duration discrimination | Vowel length – Tone | Short | Perceptual | Single interval | Evaluate | Sensorimotor controlled | Auditory | TAL | 4.5 |
| 34 | [Garraux et al., 2005](#Garraux2005) | 11 (5) | 21-40 | fMRI | Continuation | (Timing – Order) – (SI – VT) | Long | Motor | Sequence | Evaluate | Stringently controlled | Visual | MNI | 9 |
| 35 | [Geiser et al., 2012](#Geiser2012) | 17 (8) | 25.1±4.4 | fMRI | Rhythm perception | Periodic – Nonperiodic | Short | Perceptual | Sequence | Predict | Cognitively controlled | Auditory | MNI | 7.5 |
| 36 | [Godde et al., 2010](#OLE_LINK31) | 10 (2) | 18-30 | fMRI | Rhythm perception | Periodicity – localization | Short | Perceptual | Sequence | Evaluate | Cognitively controlled | Tactile | TAL | 7.5 |
| 37 | [Golan & Zakay, 2015](#OLE_LINK32) | 15 (12) | 28.47±4.37 | fMRI | Rhythm perception | Covariation with temporal variance | Short | Perceptual | Sequence | NA | Stringently controlled | Visual | MNI | 6.5 |
| 38 | [Grahn & Brett, 2007](#OLE_LINK33) | 27 (8) | 24.5±NA | fMRI | Rhythm perception | Simple – Complex | Short | Perceptual | Sequence | Evaluate | Stringently controlled | Auditory | MNI | 8 |
| 39 | [Grahn & Rowe, 2009](#OLE_LINK34) | 36 (15) | 29±NA | fMRI | Rhythm perception | Beat – Nobeat | Medium | Perceptual | Sequence | Evaluate | Stringently controlled | Auditory | MNI | 8.5 |
| 40 | [Grahn & Rowe, 2012](#OLE_LINK35) | 24 (13) | 27±NA | fMRI | Rhythm perception | Beat – Nobeat | Short | Perceptual | Sequence | Evaluate | Cognitively controlled | Auditory | MNI | 8 |
| 41 | [Gutyrchik et al., 2009](#OLE_LINK36) | 13 (6) | 29.2±6.2 | fMRI | Duration discrimination | Duration – Color | Medium | Perceptual | Single interval | Evaluate | Sensorimotor controlled | Visual | TAL | 6.5 |
|  |  |  |  |  |  | Duration – Succession |  |  |  |  |  |  |  |  |
| 42 | [Hackley et al., 2009](#OLE_LINK37) | 16 (10) | 29.4±NA | fMRI | Spatiotemporal prediction | Good clock – Bad clock | Medium | Motor | Trajectory | Predict | Cognitively controlled | Visual | MNI | 8 |
| 43 | [Harnett et al., 2016](#OLE_LINK38) | 23 (10) | 19.92±0.59 | fMRI | Conditioning | Fixed UCS – Random UCS | Long | Perceptual | Single interval | Predict | Stringently controlled | Auditory | TAL | 8.5 |
| 44 | [Harrington et al., 2009](#Harrington2009) | 20 (13) | 29±9.1 | fMRI | Duration discrimination | Time – Pitch | Medium | Perceptual | Single interval | Evaluate | Cognitively controlled | Auditory | TAL | 7 |
| 45 | [Hayashi et al., 2013](#OLE_LINK40) | 26 (14) | 19-30 | fMRI | Duration discrimination | Duration discrimination – Numerosity discrimination | Medium | Perceptual | Single interval | Evaluate | Cognitively controlled | Visual | MNI | 8 |
| 46 | [Hayashi et al., 2015](#OLE_LINK93) | 20 (11) | 18-29 | fMRI | Duration discrimination | Duration adaptation (experiment 4) | Short | Perceptual | Single interval | Evaluate | NA | Visual | MNI | 8 |
| 47 | [Henry et al., 2013](#OLE_LINK41) | 20 (10) | 21-31 | fMRI | Duration discrimination | Duration – Modulation rate | Short | Perceptual | Single interval | Evaluate | Stringently controlled | Auditory | MNI | 8.5 |
|  |  |  |  |  | Rhythm perception | Modulation rate – Duration |  |  | Sequence |  |  |  |  |  |
| 48 | [Jahanshahi et al., 2006](#OLE_LINK95) | 8 (0) | 27.5±6.8 | PET | Reproduction | Time reproduction – Control RT task | Medium | Motor | Single interval | Evaluate | Sensorimotor controlled | Auditory | MNI | 5.5 |
| 49 | [Jahanshahi et al., 2010](#Jahanshahi2010) | 8 (4) | 61±10.4 | PET | Continuation | Continuation – Synchronization | Medium | Motor | Sequence | Evaluate | Cognitively controlled | Auditory | MNI | 6.5 |
|  |  |  |  |  | Synchronization | Synchronization – Continuation |  |  |  | Predict |  |  |  |  |
| 50 | [Jäncke et al., 2000](#OLE_LINK43) | 8 (0) | 20-32 | fMRI | Synchronization | Synchronization – Continuation | Short | Motor | Sequence | Predict | Cognitively controlled | Auditory | MNI | 7 |
|  |  |  |  |  |  |  |  |  |  |  |  | Visual |  |  |
|  |  |  |  |  | Continuation | Continuation –Synchronization |  |  |  | Evaluate |  | Visual |  |  |
| 51 | [Jantzen, et al., 2007](#OLE_LINK44) | 9 (2) | 24-56 | fMRI | Continuation | Continuation – Pacing | Medium | Motor | Sequence | Evaluate | Cognitively controlled | Auditory | TAL | 7 |
|  |  |  |  |  | Syncopation | Syncopation – Synchronization |  |  |  | Evaluate |  |  |  |  |
|  |  |  |  |  | Synchronization + Syncopation | Pacing – Continuation |  |  |  | Predict & Evaluate |  |  |  |  |
| 52 | [Jantzen et al, 2005](#OLE_LINK45) | 12 (2) | 28.5±NA | fMRI | Syncopation | Syncopation – Synchronization | Medium | Motor | Sequence | Evaluate | Cognitively controlled | Auditory & visual | TAL | 7 |
| 53 | [Kawashima et al., 2000](#OLE_LINK46) | 8 (0) | 19-27 | fMRI | Continuation | Continuation – Synchronization | Medium | Motor | Sequence | Evaluate | Cognitively controlled | Visual | TAL | 7 |
|  |  |  |  |  | Synchronization | Synchronization – Continuation |  |  |  | Predict |  |  |  |  |
| 54 | [Khoshnejad et al., 2017](#OLE_LINK47) | 24 (12) | 28.8±4.8 | fMRI | Reproduction | Duration – Intensity | Long | Motor | Single interval | Evaluate | Sensorimotor controlled | Tactile | MNI | 6.5 |
| 55 | [Klahr et al., 2011](#OLE_LINK48) | 8 (0) | 21-32 | fMRI | Duration discrimination | Timing – Counting  (pre-drink condition) | Long | Perceptual | Single interval | Evaluate | Cognitively controlled | Visual | TAL | 7 |
| 56 | [Konoike et al., 2015](#OLE_LINK49) | 29 (15) | 21.4±NA | fMRI | Encoding | Encoding phase: (RXr – NXr) ∩ (RXl – NXl) ∩ (RXf – NXf) | Medium | Perceptual | Sequence | Evaluate | Cognitively controlled | Auditory | MNI | 8 |
|  |  |  |  |  | Continuation | Retrieval phase: (RXr – NXr) ∩ (RXl – NXl) ∩ (RXf – NXf) |  | Motor |  |  |  |  |  |  |
| 57 | [Kung et al., 2013](#OLE_LINK85) | 11 (5) | 24.73±5.18 | fMRI | Synchronization | Tap beat – Tap iso | Medium | Motor | Sequence | Predict | Cognitively controlled | Auditory | MNI | 8 |
|  |  |  |  |  | Encoding | Find beat – Listen iso |  | Perceptual |  | Evaluate |  |  |  |  |
| 58 | [Lejeune et al., 1997](#Lejeune1997) | 12 (0) | 20±NA | PET | Synchronization | Synchronization – Control | Long | Motor | Sequence | Predict | Sensorimotor controlled | Visual | TAL | 7 |
| 59 | [Lewis & Miall, 2002](#OLE_LINK51) | 8 (3) | 29±NA | fMRI | Reproduction | Time – Pressure | Long | Motor | Single interval | Evaluate | Cognitively controlled | Visual | MNI | 6.5 |
| 60 | [Lewis & Miall, 2003](#OLE_LINK52) | 8 (3) | 26±NA | fMRI | Duration discrimination | Time – Length | Medium | Perceptual | Single interval | Evaluate | Stringently controlled | Visual | MNI | 6 |
|  |  |  |  |  |  |  | Long |  |  |  |  |  |  |  |
| 61 | [Lewis et al., 2004](#OLE_LINK53) | 10 (5) | 27±NA | fMRI | Synchronization | Synchronization – Continuation | Medium | Motor | Sequence | Predict | Stringently controlled | Auditory | MNI | 7.5 |
|  |  |  |  |  | Continuation | Continuation – Synchronization |  |  |  | Evaluate |  |  |  |  |
| 62 | [Li et al., 2015](#OLE_LINK55) | 20 (10) | 23.6±2.1 | fMRI | Spatiotemporal prediction | TTC judgment – Luminance judgment | Long | Motor | Trajectory | Predict | Sensorimotor controlled | Visual | MNI | 7.5 |
| 63 | [Livesey et al., 2007](#Livesey2007) | 10 (7) | 21.4±NA | fMRI | Duration discrimination | Time – Color (time task easier than control) | Medium | Perceptual | Single interval | Evaluate | Cognitively controlled | Visual | TAL | 7 |
| 64 | [Lutz et al., 2000](#Lutz2000) | 10 (5) | 24.1±NA | fMRI | Synchronization | Regular – Irregular | Medium | Motor | Sequence | Predict | Cognitively controlled | Visual | MNI | 7.5 |
| 65 | [Macar et al., 2004](#OLE_LINK57) | 13 (6) | 23-56 | fMRI | Reproduction | Time – Force | Long | Motor | Single interval | Evaluate | Cognitively controlled | NA | TAL | 7 |
| 66 | [Macar et al., 2002](#OLE_LINK58) | 15 (NA) | 21±NA | PET | Reproduction | Reproduction – Sensorimotor control task | Long | Motor | Single interval | Evaluate | Sensorimotor controlled | Tactile | TAL | 7.5 |
| 67 | [Maquet et al., 1996](#OLE_LINK59) | 9 (0) | 20±NA | PET | Duration discrimination | Temporal generalization – Sensorimotor control task | Medium | Perceptual | Single interval | Evaluate | Sensorimotor controlled | Visual | TAL | 6.5 |
| 68 | [Marchant & Driver, 2012](#OLE_LINK60) | 16 (9) | 19-35 | fMRI | Orienting | Isochronous – Random | Short | Motor | Sequence | Predict | Cognitively controlled | Audiovisual | MNI | 8 |
| 69 | [Matthews et al., 2020](#OLE_LINK98) | 54 (24) | 23.48±NA | fMRI | Rhythm perception | Medium – High rhythmic complexity | Medium | Perceptual | Sequence | Evaluate | Cognitively controlled | Auditory | MNI | 8 |
| 70 | [Mayville et al., 2002](#OLE_LINK87) | 9 (4) | 20-41 | fMRI | Syncopation | Syncopate – Synchronize | Medium | Motor | Sequence | Evaluate | Cognitively controlled | Auditory | TAL | 4.5 |
| 71 | [Morillon et al., 2009](#Morillon2009) | 17 (15) | 25.4±NA | fMRI | Duration discrimination | Time – Color | Long | Perceptual | Single interval | Evaluate | Stringently controlled | Visual | MNI | 8.5 |
| 72 | [Onuki et al., 2013](#OLE_LINK62) | 16 (3) | 18-26 | fMRI | Spatiotemporal prediction | Corresponding finger – Flash marker condition | Medium | Motor | Trajectory | Predict | Sensorimotor controlled | Visual | MNI | 7.5 |
| 73 | [O'Reilly et al, 2008](#OLE_LINK63) | 12 (8) | 26.6±NA | fMRI | Spatiotemporal judgment | Temporal – Spatial | Medium | Perceptual | Trajectory | Evaluate & Predict | Stringently controlled | Visual | MNI | 8 |
| 74 | [Ortuno et al., 2002](#OLE_LINK64) | 10 (3) | 26±NA | PET | Continuation | Continuation – Synchronization | Medium | Motor | Sequence | Evaluate | Sensorimotor controlled | Auditory | TAL | 6.5 |
| 75 | [Oullier, et al., 2004](#OLE_LINK65) | 15 (5) | 23-53 | fMRI | Syncopation | Syncopation – Synchronization | Medium | Motor | Sequence | Evaluate | Cognitively controlled | Auditory | TAL | 6.5 |
| 76 | [Penhune et al., 1998](#OLE_LINK66) | 12 (6) | 22.4±NA | PET | Continuation | Complex – Isochronous | Short | Motor | Sequence | Evaluate | Cognitively controlled | Auditory | TAL | 7 |
|  |  |  |  |  |  |  |  |  |  |  |  | Visual | TAL |  |
| 77 | [Pfeuty et al., 2014](#OLE_LINK67) | 26 (17) | 23±4 | fMRI | Duration discrimination | Duration discrimination – Color discrimination | Medium | Perceptual | Single interval | Evaluate | Cognitively controlled | Visual | MNI | 8 |
| 78 | [Rao et al., 2001](#OLE_LINK68) | 17 (15) | 23.9±NA | fMRI | Duration discrimination | Time – Pitch | Medium | Perceptual | Single interval | Evaluate | Cognitively controlled | Auditory | TAL | 6.5 |
| 79 | [Schubotz et al., 2000](#OLE_LINK69) | 20 (13) | 23.5±NA | fMRI | Rhythm perception | (Visual + Auditory rhythm monitoring) – (Color + Pitch Monitoring) | Medium | Perceptual | Sequence | Evaluate | Sensorimotor controlled | Auditory & visual | TAL | 6.5 |
| 80 | [Schubotz & von Cramon, 2001](#Schubotz20011) | 12 (8) | 23.6±NA | fMRI | Rhythm perception | Timing – (Object/Space) | Medium | Perceptual | Sequence | Evaluate | Cognitively controlled | Visual | TAL | 7 |
| 81 | [Schubotz & von Cramon, 2001b](#Schubotz2001) | 12 (6) | 23±NA | fMRI | Rhythm perception | Interval – Ordinal | Medium | Perceptual | Sequence | Evaluate | Cognitively controlled | Visual | TAL | 7 |
| 82 | [Schubotz, et al., 2003](#OLE_LINK89) | 16 (8) | 24.5±NA | fMRI | Rhythm perception | Timing – (Object/Space) | Medium | Perceptual | Sequence | Evaluate | Stringently controlled | Auditory | TAL | 7.5 |
| 83 | [Shergill et al., 2006](#Shergill2006) | 8 (0) | 29±5 | fMRI | Continuation | Self-paced – Cued articulation | Long | Motor | Sequence | Evaluate | Sensorimotor controlled | NA | TAL | 5.5 |
| 84 | [Shih et al., 2009](#OLE_LINK72) | 17 (8) | 23.8±3.5 | fMRI | Duration discrimination | Visual duration discrimination – Sensorimotor control | Short | Perceptual | Single interval | Evaluate | Sensorimotor controlled | Auditory | MNI | 7.5 |
|  |  |  |  |  |  | Auditory duration discrimination – Sensorimotor control |  |  |  |  |  | Visual |  |  |
| 85 | [Shih et al., 2010](#OLE_LINK73) | 21 (11) | 23.38±NA | fMRI | Duration discrimination | Difficult – Easy | Short | Perceptual | Single interval | Evaluate | Cognitively controlled | Visual | TAL | 7 |
| 86 | [Skagerlund et al., 2016](#OLE_LINK74) | 24 (14) | 24.33±2.41 | fMRI | Duration discrimination | Duration discrimination – Color discrimination | Medium | Perceptual | Single interval | Evaluate | Sensorimotor controlled | Visual | MNI | 6.5 |
| 87 | [Smith et al., 2003](#OLE_LINK75) | 20 (0) | 29.5±NA | fMRI | Duration discrimination | Timing – Order | Medium | Perceptual | Single interval | Evaluate | Sensorimotor controlled | Visual | TAL | 7.5 |
| 88 | [Teki et al., 2011](#OLE_LINK76) | 18 (9) | 22.17±NA | fMRI | Duration discrimination | Irregular – Regular | Short | Perceptual | Sequence | Evaluate | Stringently controlled | Auditory | MNI | 7.5 |
|  |  |  |  |  |  | Regular – Irregular |  |  |  |  |  |  |  |  |
| 89 | [Tipples et al., 2013](#OLE_LINK77) | 17 (NA) | NA | fMRI | Duration discrimination | Time – Sex | Medium | Perceptual | Single interval | Evaluate | Sensorimotor controlled | Visual | MNI | 7.5 |
| 90 | [Tomasi et al., 2014](#OLE_LINK78) | 36 (18) | 27±6 | fMRI | Orienting | Target onset prediction – (Sensorimotor + Spatial attention + Working memory) | Medium | Motor | Sequence | Predict | Cognitively controlled | Visual | MNI | 8 |
| 91 | [Tregellas et al., 2006](#OLE_LINK79) | 20 (9) | 41.2±9.7 | fMRI | Duration discrimination | Duration discrimination – Baseline | Short | Perceptual | Single interval | Evaluate | Sensorimotor controlled | Auditory | MNI | 8 |
|  |  |  |  |  |  | Difficult – Easy |  |  |  |  | Cognitively controlled |  |  |  |
| 92 | [Üstün et al., 2017](#OLE_LINK80) | 15 (7) | 22.46±2.09 | fMRI | Spatiotemporal judgment | Time – Memory | Long | Perceptual | Trajectory | Predict & Evaluate | Cognitively controlled | Visual | TAL | 8 |
| 93 | [Visalli et al., 2019](#Visalli2019) | 22 (10) | 26±4 | fMRI | Orienting | Covariation with temporal hazard | Medium | Motor | Single interval | Predict | NA | Visual | MNI | 7.5 |
| 94 | [Wiener et al., 2014](#Wiener2014) | 25 (14) | 25±3.8 | fMRI | Duration discrimination | Time – Color | Medium | Perceptual | Single interval | Evaluate | Cognitively controlled | Visual | MNI | 7.5 |
| 95 | [Wittmann et al., 2010](#OLE_LINK83) | 14 (7) | 25±NA | fMRI | Encoding | Encoding phase – Control phase | Long | Perceptual | Single interval | Evaluate | Sensorimotor controlled | Auditory | TAL | 8 |
|  |  |  |  |  | Reproduction | Reproduction phase – Control phase |  | Motor |  |  |  |  |  |  |

A summary of the included experiments, including the number of participants (number of female participants), their mean age, imaging modality, task paradigm, contrast, duration range, sensorimotor processing, stimulus contiguity, task goal, control task stringency, sensory modality, and standard stereotactic space.

# Supplementary Table 2. Results of the All-effects Analysis.

#

|  | Cluster 1 | Cluster2 | Cluster 3 | Cluster 4 | Cluster 5 | Cluster 6 | Cluster 7 | Cluster8 |
| --- | --- | --- | --- | --- | --- | --- | --- | --- |
| Center coordinates | -32/-64/-32 | 22/8/2 | -42/14/2 | 46/18/4 | 42/40/22 | 2/12/54 | -42/-48/44 | 48/-40/46 |
| Cluster size | 272 | 473 | 1302 | 1340 | 323 | 1504 | 238 | 430 |
| Cluster label | Left cerebellum curs I | Right putamen, pallidum and caudate | Left insula, frontal operculum cortex, inferior frontal gyrus, precentral gyrus, putamen, pallidum, and caudate | Right insula, frontal operculum cortex, inferior frontal gyrus, and precentral gyrus | Right middle frontal gyrus | Pre-SMA, SMA-proper, and paracingulate gyrus | Left inferior parietal sulcus | Right inferior parietal lobule |
| Contributing articles | Araneda2016  Aso2010  Bengtsson2004  Bengtsson2006  Beudel2008  Bueti2008  Chen2008_1  Chen2008_2  Coull2012_2  Coull2012  Coull1998_1  Dormal2011  Grahn2013  Jantzen2007  Jantzen2005  Kawashima2000  Klahr2011  Lewis2003  Lewis2004  Morillon2009  Oullier2005  Schubotz2000  Kung2013  Mayville2002 | Araneda2016  Bengtsson2004  Bueti2011  Bueti2008  Coull2012_2  Garraux2005  Golan2015  Grahn2007  Grahn2009  Grahn2013  Gutyrchik2009  Khoshnejad2017  Lewis2004  Li2015  Marchant2013  Morillon2009  Rao2001  Schubotz2000  Schubotz2001  Shih2010  Teki2011  Tipples2013  Tomasi2015  Tregellas2006  Kung2013  Mayville2002  Schubotz2001(2)  Geiser2012  Matthews2019 | Araneda2016  Aso2010  Bengtsson2004  Bengtsson2006  Beudel2008  Bueti2011  Bueti2008  Carlsson2006  Carvalho2016  Coull2016  Coull2012_2  Coull2012  Coull1998_1  Coull2001  Coull2008_2  Coull2004  Ferrandez2003  Grahn2007  Grahn2009  Grahn2013  Gutyrchik2009  Hackley2009  Harrington2010  Hayashi2013  Henry2013  Jahanshahi2010  Kawashima2000  Klahr2011  Konoike2015  Lewis2003  Lewis2004  Li2015  Livesey2007  Marchant2013  Morillon2009  Schubotz2000  Schubotz2001  Shih2009  Skagerlund2016  Teki2011  Tipples2013  Tomasi2015  Tregellas2006  Wittmann2010  Billington2010  Kung2013  Mayville2002  Schubotz2001(2)  Schubotz2003  Field2005  Geiser2012  Matthews2019 | Araneda2016  Aso2010  Bengtsson2004  Bengtsson2006  Beudel2008  Bueti2011  Carlsson2006  Carvalho2016  Chen2008_2  Coull2012_2  Coull2012  Coull2004  Ferrandez2003  Gandour2002  Grahn2007  Grahn2013  Hayashi2013  Jahanshahi2010  Khoshnejad2017  Klahr2011  Konoike2015  Lejeune1997  Lewis2002  Lewis2003  Lewis2004  Li2015  Livesey2007  Maquet1996  Marchant2013  Morillon2009  O'reilly2008  Pfeuty2015  Rao2001  Schubotz2000  Schubotz2001  Shergill2006  Skagerlund2016  Smith2003  Teki2011  Tipples2013  Tomasi2015  Tregellas2006  Wittmann2010  Kung2013  Mayville2002  Schubotz2001(2)  Schubotz2003  Cui2009 | Beudel2008  Bueti2008  Carvalho2016  Chen2008_1  Chen2008_2  Coull2012_2  Coull2012  Coull2001  Dormal2011  Gandour2002  Grahn2013  Hayashi2013  Henry2013  Jahanshahi2010  Klahr2011  Lewis2002  Lewis2003  Lewis2004  Macar2002  Morillon2009  O'reilly2008  Ortuno2002  Shergill2006  Smith2003  Tipples2013  Tregellas2006  Billington2010  Kung2013  Mayville2002  Bueti2010 | Apaydin2018  Araneda2016  Bengtsson2004  Bengtsson2005  Beudel2008  Bueti2011  Bueti2008  Carvalho2016  Chen2008_1  Chen2008_2  Coull2015  Coull2012_2  Coull2012  Coull2008  Coull2004  Dormal2011  Ferrandez2003  Grahn2013  Gutyrchik2009  Harrington2010  Hayashi2013  Henry2013  Jantzen2007  Jantzen2005  Klahr2011  Konoike2015  Lejeune1997  Lewis2002  Lewis2003  Lewis2004  Macar2004  Macar2002  Maquet1996  O'reilly2008  Ortuno2002  Oullier2005  Schubotz2000  Schubotz2001  Shergill2006  Shih2009  Skagerlund2016  Smith2003  Teki2011  Tipples2013  Tomasi2015  Tregellas2006  Wittmann2010  Billington2010  Kung2013  Mayville2002  Schubotz2001(2)  Matthews2019  Cui2009 | Apaydin2018  Araneda2016  Carlsson2006  Carvalho2016  Chen2008_1  Chen2008_2  Coull2016  Coull2012_2  Coull2012  Coull1998_1  Coull2001  Coull2004  Davranche2011  Dormal2011  Henry2013  Konoike2015  Lewis2003  Lewis2004  Marchant2013  Oullier2005  Schubotz2000  Shih2009  Bolger2014  Matthews2019 | Apaydin2018  Aso2010  Bueti2011  Bueti2008  Carlsson2006  Carvalho2016  Chen2008_2  Coull2012_2  Coull2004  Dormal2011  Grahn2013  Henry2013  Jantzen2007  Khoshnejad2017  Klahr2011  Konoike2015  Lejeune1997  Lewis2002  Lewis2003  Lewis2004  Macar2002  Maquet1996  O'reilly2008  Oullier2005  Schubotz2000  Shergill2006  Skagerlund2016  Tomasi2015  Ustun2017  Wittmann2010  Billington2010  Bueti2010  Matthews2019 |
| Short | 6.70% | 28.75% | 18.73% | 13.22% | 9.19% | 14.48% | 22.23% | 15.04% |
| Medium | 78.81% | 62.69% | 62.97% | 57.37% | 63.51% | 67.04% | 66.08% | 51.33% |
| Long | 10.11% | 8.55% | 11.73% | 24.49% | 20.14% | 15.52% | 5.06% | 22.72% |
| Visual | 39.39% | 27.65% | 50.57% | 52.00% | 60.52% | 42.32% | 34.24% | 58.08% |
| Auditory | 40.90% | 45.87% | 33.72% | 30.05% | 33.84% | 40.50% | 39.67% | 31.55% |
| Tactile | 0.00% | 3.93% | 1.64% | 4.69% | 3.62% | 1.80% | 2.99% | 3.92% |
| Single interval | 38.05% | 30.89% | 43.57% | 50.32% | 53.47% | 42.86% | 39.24% | 40.36% |
| Sequence | 61.51% | 66.65% | 46.08% | 41.35% | 32.61% | 45.98% | 48.50% | 33.85% |
| Trajectory | 0.45% | 2.46% | 9.81% | 8.32% | 11.10% | 8.83% | 5.09% | 18.55% |
| Perceptual | 35.80% | 60.85% | 61.70% | 51.14% | 41.65% | 52.12% | 46.36% | 47.83% |
| Motor | 58.12% | 31.69% | 26.19% | 31.41% | 54.98% | 45.98% | 39.97% | 40.53% |
| Quantification | 65.82% | 73.97% | 68.64% | 67.18% | 54.56% | 70.63% | 50.37% | 63.09% |
| Prediction | 15.03% | 15.16% | 18.58% | 17.62% | 25.51% | 15.53% | 41.41% | 16.89% |
| Task control | 26.11% | 23.94% | 20.87% | 25.32% | 32.89% | 27.78% | 15.38% | 27.88% |
| Cognitive load control | 56.28% | 41.66% | 46.23% | 53.54% | 48.83% | 44.72% | 64.37% | 43.61% |
| Stringent control | 17.61% | 30.35% | 28.86% | 18.30% | 18.01% | 24.78% | 20.25% | 28.50% |
| Duration discrimination | 22.02% | 19.75% | 33.42% | 31.34% | 33.74% | 31.91% | 41.41% | 20.72% |
| Temporal orienting | 0.11% | 7.82% | 10.63% | 9.95% | 4.07% | 1.51% | 27.21% | 6.34% |
| Reproduction | 2.55% | 6.99% | 1.80% | 4.42% | 6.45% | 7.29% | 0.00% | 10.70% |
| Rhythm perception | 13.36% | 41.10% | 21.72% | 14.85% | 2.74% | 15.05% | 21.89% | 8.72% |
| Spatiotemporal prediction | 0.02% | 2.46% | 7.16% | 5.54% | 8.79% | 4.11% | 4.10% | 8.32% |
| Spatiotemporal judgement | 0.43% | 0.00% | 2.65% | 2.78% | 2.32% | 2.82% | 1.00% | 10.23% |
| Continuation | 13.04% | 2.67% | 1.04% | 4.05% | 4.94% | 6.64% | 0.00% | 1.89% |
| Synchronization | 14.90% | 0.00% | 0.00% | 2.12% | 12.65% | 9.91% | 10.10% | 2.21% |
| Other tasks | 13.42% | 5.24% | 3.29% | 1.60% | 3.86% | 2.42% | 0.50% | 2.20% |

A summary of the obtained clusters, including the center coordinates, cluster size, regional cluster extent, contributing studies, and proportional contribution of each temporal characteristic for the all-effects analysis.

# Supplementary Table 3. Results of the Short Duration Analysis

|  | Cluster 1 | Cluster2 |
| --- | --- | --- |
| Center coordinates | 22/8/-2 | -30/22/0 |
| Cluster size | 143 | 189 |
| Cluster label | Right putamen | Left insula and putamen |
| Contributing articles | Grahn2007  Grahn2013  Marchant2013  Shih2010  Teki2011  Tregellas2006  Geiser2012 | Grahn2013  Marchant2013  Shih2009  Teki2011  Tregellas2006  Billington2010  Field2005 |
| Short | 100.00% | 100.00% |
| Medium | 0.00% | 0.00% |
| Long | 0.00% | 0.00% |
| Visual | 10.24% | 18.92% |
| Auditory | 72.80% | 47.82% |
| Tactile | 0.00% | 0.00% |
| Single interval | 26.83% | 34.99% |
| Sequence | 73.17% | 46.09% |
| Trajectory | 0.00% | 18.92% |
| Perceptual | 83.05% | 72.00% |
| Motor | 16.95% | 28.00% |
| Quantification | 66.34% | 61.64% |
| Prediction | 33.66% | 38.36% |
| Task control | 0.00% | 13.88% |
| Cognitive load control | 57.71% | 43.77% |
| Stringent control | 25.70% | 21.25% |
| Duration discrimination | 29.57% | 45.92% |
| Temporal orienting | 16.95% | 19.38% |
| Reproduction | 0.00% | 0.00% |
| Rhythm perception | 53.47% | 15.78% |
| Spatiotemporal prediction | 0.00% | 18.92% |
| Spatiotemporal judgement | 0.00% | 0.00% |
| Continuation | 0.00% | 0.00% |
| Synchronization | 0.00% | 0.00% |
| Other tasks | 0.00% | 0.00% |

A summary of the obtained clusters, including the center coordinates, cluster size, regional cluster extent, contributing studies, and proportional contribution of each temporal characteristic for the short duration analysis.

# Supplementary Table 4. Results of the Medium Duration Analysis

|  | Cluster 1 | Cluster2 | Cluster 3 | Cluster 4 | Cluster 5 | Cluster 6 | Cluster 7 | Cluster 8 | Cluster 9 |
| --- | --- | --- | --- | --- | --- | --- | --- | --- | --- |
| Center coordinates | -30/-66/-32 | -18/8/-2 | -46/16/0 | 46/16/0 | 20/8/4 | 44/40/22 | -40/-48/44 | 2/12/54 | 46/-44/44 |
| Cluster size | 317 | 187 | 617 | 766 | 272 | 186 | 165 | 1132 | 137 |
| Cluster label | Left cerebellum crus I | Left putamen, pallidum, and caudate | Left insula, frontal operculum cortex, inferior frontal gyrus, and precentral gyrus | Right insula, frontal operculum cortex, inferior frontal gyrus, and precentral gyrus | Right putamen, pallidum, and caudate | Right middle frontal gyrus | Left inferior parietal sulcus | Pre-SMA, SMA-proper, paracingulate gyrus | Right inferior parietal sulcus, inferior parietal lobule |
| Contributing articles | Araneda2016  Aso2010  Bengtsson2004  Bengtsson2006  Bueti2008  Chen2008_1  Chen2008_2  Coull2012_2  Coull2012  Coull1998_1  Dormal2011  Jantzen2007  Jantzen2005  Kawashima2000  Lewis2003  Lewis2004  Onuki2015  O'reilly2008  Oullier2005  Schubotz2000  Smith2003  Kung2013  Mayville2002 | Araneda2016  Bueti2008  Coull2016  Coull2001  Coull2004  Ferrandez2003  Grahn2009  Gutyrchik2009  Harrington2010  Hayashi2013  Livesey2007  Schubotz2000  Tipples2013  Tomasi2015  Kung2013  Mayville2002  Schubotz2001_2  Matthews2019 | Araneda2016  Aso2010  Bengtsson2004  Bengtsson2006  Bueti2011  Bueti2008  Coull2012  Coull2008_2  Coull2004  Ferrandez2003  Hayashi2013  Jahanshahi2010  Kawashima2000  Lewis2003  Lewis2004  Livesey2007  Schubotz2000  Schubotz2001  Skagerlund2016  Tipples2013  Tomasi2015  Kung2013  Mayville2002  Schubotz2001_2  Schubotz2003  Matthews2019 | Araneda2016  Aso2010  Bengtsson2004  Bengtsson2006  Bueti2011  Chen2008_2  Coull2012_2  Coull2012  Coull2004  Ferrandez2003  Hayashi2013  Jahanshahi2010  Konoike2015  Lewis2003  Lewis2004  Livesey2007  Maquet1996  O'reilly2008  Oullier2005  Pfeuty2015  Rao2001  Schubotz2000  Schubotz2001  Skagerlund2016  Smith2003  Tipples2013  Tomasi2015  Kung2013  Mayville2002  Schubotz2001_2  Schubotz2003  Visalli2019 | Araneda2016  Bengtsson2004  Bueti2011  Bueti2008  Coull2012_2  Grahn2009  Gutyrchik2009  Lewis2004  Rao2001  Schubotz2000  Schubotz2001  Tipples2013  Tomasi2015  Kung2013  Mayville2002  Schubotz2001_2  Matthews2019 | Chen2008_1  Chen2008_2  Coull2012_2  Coull2012  Coull2001  Dormal2011  Hayashi2013  Jahanshahi2010  Lewis2003  O'reilly2008  Ortuno2002  Smith2003  Tipples2013  Kung2013  Mayville2002 | Araneda2016  Chen2008_1  Chen2008_2  Coull2012_2  Coull1998_1  Coull2001  Coull2004  Davranche2011  Dormal2011  Konoike2015  Lewis2003  Lewis2004  Schubotz2000  Matthews2019 | Araneda2016  Bengtsson2004  Bengtsson2005  Bueti2011  Bueti2008  Chen2008_1  Chen2008_2  Coull2015  Coull2012_2  Coull2012  Coull2008  Coull2004  Dormal2011  Ferrandez2003  Gutyrchik2009  Harrington2010  Hayashi2013  Jantzen2007  Jantzen2005  Konoike2015  Lewis2003  Lewis2004  Maquet1996  O'reilly2008  Ortuno2002  Oullier2005  Schubotz2000  Schubotz2001  Skagerlund2016  Smith2003  Tipples2013  Kung2013  Mayville2002  Schubotz2001_2  Matthews2019 | Bueti2008  Chen2008_2  Coull2012_2  Coull2004  Dormal2011  Jantzen2007  Konoike2015  Lewis2003  Lewis2004  Maquet1996  O'reilly2008  Schubotz2000  Skagerlund2016  Tomasi2015  Matthews2019 |
| Short | 0.00% | 0.00% | 0.00% | 0.00% | 0.00% | 0.00% | 0.00% | 0.00% | 0.00% |
| Medium | 100.00% | 100.00% | 100.00% | 100.00% | 100.00% | 100.00% | 100.00% | 100.00% | 100.00% |
| Long | 0.00% | 0.00% | 0.00% | 0.00% | 0.00% | 0.00% | 0.00% | 0.00% | 0.00% |
| Visual | 34.49% | 48.68% | 55.10% | 61.18% | 35.81% | 58.15% | 37.38% | 43.45% | 54.71% |
| Auditory | 42.70% | 34.85% | 29.13% | 27.09% | 37.92% | 41.85% | 47.64% | 42.73% | 31.88% |
| Tactile | 0.00% | 0.00% | 0.00% | 0.00% | 0.00% | 0.00% | 0.00% | 0.00% | 0.00% |
| Single interval | 31.65% | 38.75% | 36.51% | 50.07% | 28.24% | 53.32% | 37.38% | 40.07% | 43.18% |
| Sequence | 68.10% | 61.25% | 59.77% | 48.49% | 71.71% | 41.85% | 62.62% | 55.41% | 50.90% |
| Trajectory | 0.25% | 0.00% | 3.71% | 1.43% | 0.05% | 4.83% | 0.00% | 4.53% | 5.92% |
| Perceptual | 21.03% | 78.31% | 54.88% | 52.31% | 64.09% | 36.40% | 47.26% | 50.85% | 50.58% |
| Motor | 71.93% | 13.94% | 32.08% | 26.45% | 27.62% | 60.36% | 38.46% | 41.15% | 37.05% |
| Quantification | 59.03% | 79.43% | 71.43% | 66.49% | 79.71% | 42.51% | 54.01% | 69.96% | 66.09% |
| Prediction | 17.79% | 12.77% | 10.29% | 10.68% | 3.12% | 24.67% | 35.06% | 12.52% | 9.61% |
| Task control | 30.49% | 13.04% | 19.84% | 24.10% | 28.41% | 36.81% | 14.02% | 29.36% | 41.49% |
| Cognitive load control | 56.48% | 60.06% | 49.71% | 57.34% | 43.88% | 50.40% | 64.27% | 44.57% | 23.66% |
| Stringent control | 13.03% | 26.90% | 30.45% | 18.46% | 27.71% | 12.79% | 21.71% | 26.07% | 34.85% |
| Duration discrimination | 11.98% | 36.31% | 27.58% | 37.39% | 17.14% | 31.57% | 9.88% | 33.78% | 30.80% |
| Temporal orienting | 0.11% | 12.77% | 10.24% | 7.75% | 3.07% | 5.14% | 17.90% | 0.02% | 5.61% |
| Reproduction | 3.88% | 1.00% | 1.34% | 0.00% | 4.48% | 0.03% | 0.00% | 3.14% | 8.99% |
| Rhythm perception | 8.91% | 42.00% | 23.63% | 13.48% | 46.95% | 0.00% | 37.37% | 15.67% | 13.86% |
| Spatiotemporal prediction | 0.11% | 0.00% | 0.05% | 0.00% | 0.05% | 0.00% | 0.00% | 0.00% | 0.00% |
| Spatiotemporal judgement | 0.14% | 0.00% | 3.66% | 1.43% | 0.00% | 4.83% | 0.00% | 1.40% | 5.92% |
| Continuation | 13.91% | 0.00% | 2.57% | 3.18% | 2.94% | 4.87% | 0.00% | 9.19% | 0.07% |
| Synchronization | 17.57% | 0.00% | 0.00% | 2.93% | 0.00% | 19.53% | 17.16% | 12.51% | 4.00% |
| Other tasks | 18.02% | 0.11% | 8.80% | 2.59% | 6.67% | 6.05% | 0.06% | 3.27% | 0.00% |

A summary of the obtained clusters, including the center coordinates, cluster size, regional cluster extent, contributing studies, and proportional contribution of each temporal characteristic for the medium duration analysis.

# Supplementary Table 5. Results of the Long Duration Analysis

|  | Cluster 1 | Cluster2 | Cluster 3 | Cluster 4 | Cluster 5 |
| --- | --- | --- | --- | --- | --- |
| Center coordinates | -42/18/-2 | 42/20/4 | 6/26/38 | 58/-38/44 | 4/12/58 |
| Cluster size | 117 | 406 | 153 | 134 | 125 |
| Cluster label | Left insula and frontal operculum cortex | Right insula, frontal operculum cortex, inferior frontal gyrus, precentral gyrus | Paracingulate gyrus | Right inferior parietal lobule | Pre-SMA |
| Contributing articles | Beudel2008  Carvalho2016  Klahr2011  Lewis2003  Morillon2009  Wittmann2010 | Beudel2008  Carvalho2016  Khoshnejad2017  Klahr2011  Lejeune1997  Lewis2002  Lewis2003  Li2015  Morillon2009  Shergill2006  Wittmann2010 | Apaydin2018  Beudel2008  Klahr2011  Lejeune1997  Lewis2002  Lewis2003  Macar2002  Shergill2006 | Apaydin2018  Carvalho2016  Khoshnejad2017  Klahr2011  Lejeune1997  Lewis2002  Lewis2003  Macar2002  Morillon2009  Ustun2017 | Carvalho2016  Klahr2011  Lejeune1997  Lewis2002  Macar2004  Macar2002  Wittmann2010  Cui2009 |
| Short | 0.00% | 0.00% | 0.00% | 0.00% | 0.00% |
| Medium | 0.00% | 0.00% | 0.00% | 0.00% | 0.00% |
| Long | 100.00% | 100.00% | 100.00% | 100.00% | 100.00% |
| Visual | 75.17% | 64.53% | 75.09% | 65.68% | 47.17% |
| Auditory | 24.83% | 20.10% | 0.00% | 0.08% | 21.06% |
| Tactile | 0.00% | 12.25% | 18.82% | 34.24% | 18.84% |
| Single interval | 76.18% | 69.16% | 46.10% | 41.55% | 83.01% |
| Sequence | 0.00% | 3.66% | 17.73% | 15.42% | 0.48% |
| Trajectory | 23.82% | 27.18% | 36.16% | 43.03% | 16.52% |
| Perceptual | 65.20% | 32.88% | 51.36% | 48.75% | 14.95% |
| Motor | 9.97% | 47.02% | 48.64% | 51.17% | 63.99% |
| Quatification | 76.18% | 72.30% | 52.19% | 41.56% | 80.81% |
| Prediction | 9.97% | 22.46% | 11.68% | 16.13% | 19.19% |
| Task control | 24.83% | 38.91% | 36.55% | 49.73% | 40.37% |
| Cognitive load control | 30.76% | 37.95% | 24.59% | 48.41% | 57.41% |
| Stringent control | 44.41% | 23.14% | 38.86% | 1.86% | 0.01% |
| Duration discrimination | 51.35% | 23.14% | 15.23% | 6.44% | 14.95% |
| Temporal orienting | 0.00% | 0.00% | 0.00% | 0.00% | 2.21% |
| Reproduction | 0.00% | 21.42% | 30.88% | 35.03% | 44.79% |
| Rhythm perception | 0.00% | 0.00% | 0.00% | 0.00% | 0.00% |
| Spatiotemporal prediction | 9.97% | 21.94% | 0.03% | 0.72% | 16.52% |
| Spatiotemporal judgement | 13.85% | 5.24% | 36.13% | 42.32% | 0.00% |
| Continuation | 0.00% | 3.14% | 6.09% | 0.01% | 0.02% |
| Synchronization | 0.00% | 0.52% | 11.65% | 15.41% | 0.46% |
| Other tasks | 0.00% | 0.00% | 0.00% | 0.00% | 0.00% |

A summary of the obtained clusters, including the center coordinates, cluster size, regional cluster extent, contributing studies, and proportional contribution of each temporal characteristic for the long duration analysis.

**Supplementary Table 6. Results of the Visual Modality Analysis**

|  | Cluster 1 | Cluster2 | Cluster 3 | Cluster 4 | Cluster 5 | Cluster 6 |
| --- | --- | --- | --- | --- | --- | --- |
| Center coordinates | 40/20/2 | -42/18/2 | 42/40/22 | 50/12/22 | 52/-38/44 | 2/18/50 |
| Cluster size | 576 | 474 | 184 | 109 | 246 | 682 |
| Cluster label | Right insula, frontal operculum cortex, inferior frontal gyrus | Left insula, frontal operculum cortex, inferior frontal gyrus | Middle frontal gyrus | Right inferior frontal gyrus pars opercularis and precentral gyrus | Right inferior parietal lobule | pre-SMA, paracingulate gyrus |
| Contributing articles | Aso2010  Beudel2008  Carvalho2016  Coull2012_2  Coull2012  Coull2004  Ferrandez2003  Hayashi2013  Klahr2011  Lejeune1997  Lewis2002  Lewis2003  Livesey2007  Maquet1996  Morillon2009  O'reilly2008  Pfeuty2015  Schubotz2001  Skagerlund2016  Tipples2013  Tomasi2015  Schubotz2001_2 | Aso2010  Beudel2008  Bueti2008  Carvalho2016  Coull2016  Coull2012  Coull2008_2  Coull2004  Ferrandez2003  Hackley2009  Hayashi2013  Kawashima2000  Klahr2011  Lewis2003  Livesey2007  Morillon2009  Schubotz2001  Skagerlund2016  Tipples2013  Tomasi2015  Billington2010  Schubotz2001_2  Field2005 | Carvalho2016  Coull2012_2  Coull2012  Coull2008  Coull2001  Dormal2011  Hayashi2013  Klahr2011  Lewis2002  Lewis2003  Morillon2009  O'reilly2008  Smith2003  Tipples2013  Billington2010  Bueti2010 | Aso2010  Carvalho2016  Coull2012_2  Hayashi2013  Klahr2011  Li2015  O'reilly2008  Smith2003  Tipples2013  Tomasi2015  Schubotz2001_2 | Apaydin2018  Aso2010  Bueti2008  Carvalho2016  Coull2012_2  Coull2012  Coull2004  Dormal2011  Klahr2011  Lejeune1997  Lewis2002  Lewis2003  Maquet1996  O'reilly2008  Skagerlund2016  Tomasi2015  Ustun2017  Billington2010  Bueti2010  Field2005  Hayashi2015 | Apaydin2018  Araneda2016  Beudel2008  Bueti2008  Carvalho2016  Coull2015  Coull2012_2  Coull2012  Coull2004  Dormal2011  Ferrandez2003  Gutyrchik2009  Hayashi2013  Klahr2011  Lejeune1997  Lewis2002  Lewis2003  Maquet1996  O'reilly2008  Schubotz2001  Shih2009  Skagerlund2016  Smith2003  Tipples2013  Tomasi2015  Billington2010  Schubotz2001_2  Cui2009 |
| Short | 0.00% | 10.61% | 4.53% | 0.02% | 9.80% | 9.71% |
| Medium | 63.25% | 61.15% | 58.61% | 63.28% | 36.36% | 59.68% |
| Long | 30.02% | 16.72% | 25.11% | 36.69% | 38.75% | 22.97% |
| Visual | 100.00% | 100.00% | 100.00% | 100.00% | 100.00% | 100.00% |
| Auditory | 0.00% | 0.00% | 0.00% | 0.00% | 0.00% | 0.00% |
| Tactile | 0.00% | 0.00% | 0.00% | 0.00% | 0.00% | 0.00% |
| Single interval | 71.98% | 61.34% | 80.71% | 61.32% | 54.18% | 68.66% |
| Sequence | 13.41% | 18.75% | 0.00% | 15.08% | 9.89% | 10.89% |
| Trajectory | 14.61% | 19.91% | 19.29% | 23.60% | 35.93% | 20.45% |
| Perceptual | 66.60% | 72.68% | 55.52% | 52.14% | 43.61% | 70.83% |
| Motor | 26.89% | 22.46% | 44.48% | 47.47% | 52.87% | 29.17% |
| Quantification | 71.69% | 69.12% | 56.55% | 52.08% | 45.03% | 68.05% |
| Prediction | 17.65% | 26.63% | 21.36% | 23.80% | 37.62% | 18.25% |
| Task control | 24.05% | 21.31% | 43.02% | 38.77% | 24.80% | 49.82% |
| Cognitive load control | 52.78% | 47.05% | 32.78% | 60.75% | 56.22% | 27.07% |
| Stringent control | 23.16% | 31.64% | 24.19% | 0.48% | 18.80% | 19.44% |
| Duration discrimination | 56.22% | 54.57% | 52.23% | 37.28% | 26.42% | 57.04% |
| Temporal orienting | 7.50% | 10.65% | 5.36% | 0.66% | 16.02% | 3.76% |
| Reproduction | 4.47% | 1.58% | 4.32% | 0.00% | 15.09% | 4.32% |
| Rhythm perception | 4.48% | 8.11% | 0.00% | 14.40% | 0.00% | 6.69% |
| Spatiotemporal prediction | 8.72% | 15.97% | 16.00% | 23.14% | 18.89% | 10.38% |
| Spatiotemporal judgement | 5.90% | 3.93% | 3.29% | 0.46% | 17.04% | 7.10% |
| Continuation | 0.00% | 0.00% | 0.00% | 0.00% | 0.00% | 0.00% |
| Synchronization | 1.44% | 0.00% | 0.00% | 0.00% | 2.71% | 4.10% |
| Other tasks | 0.00% | 0.00% | 0.00% | 0.00% | 0.00% | 0.00% |
| Encoding | 0.00% | 0.00% | 0.00% | 0.00% | 0.00% | 0.00% |

A summary of the obtained clusters, including the center coordinates, cluster size, regional cluster extent, contributing studies, and proportional contribution of each temporal characteristic for the visual modality analysis.

**Supplementary Table 7. Results of the Auditory Modality Analysis**

|  | Cluster 1 | Cluster2 | Cluster 3 | Cluster 4 | Cluster 5 |
| --- | --- | --- | --- | --- | --- |
| Center coordinates | 24/6/4 | -32/18/-2 | 54/12/22 | 0/12/52 | 4/-4/64 |
| Cluster size | 236 | 269 | 100 | 582 | 170 |
| Cluster label | Right putamen | Left insula, frontal operculum cortex, pumen, pallidum, and caudate | Right inferior frontal gyrus pars opercularis and precentral gyrus | Pre-SMA, paracingulate gyrus | SMA-proper |
| Contributing articles | Araneda2016  Grahn2007  Grahn2009  Grahn2013  Lewis2004  Rao2001  Teki2011  Tregellas2006  Kung2013  Mayville2002  Geiser2012  Matthews2019 | Araneda2016  Bengtsson2006  Grahn2007  Grahn2009  Grahn2013  Harrington2010  Jahanshahi2010  Lewis2004  Shih2009  Teki2011  Tregellas2006  Wittmann2010  Kung2013  Mayville2002  Geiser2012  Matthews2019 | Araneda2016  Gandour2002  Grahn2013  Jahanshahi2010  Konoike2015  Lewis2004  Ortuno2002  Oullier2005  Tregellas2006  Wittmann2010  Kung2013 | Bengtsson2005  Chen2008_1  Chen2008_2  Grahn2013  Harrington2010  Henry2013  Jantzen2007  Konoike2015  Lewis2004  Ortuno2002  Oullier2005  Shih2009  Teki2011  Tregellas2006  Wittmann2010  Kung2013  Mayville2002  Matthews2019 | Araneda2016  Bengtsson2005  Chen2008_1  Chen2008_2  Grahn2013  Jantzen2007  Lewis2004  Shih2009  Teki2011  Wittmann2010  Matthews2019 |
| Short | 40.62% | 30.03% | 36.35% | 19.59% | 29.20% |
| Medium | 59.37% | 60.94% | 56.71% | 74.92% | 69.80% |
| Long | 0.02% | 9.04% | 6.94% | 5.49% | 1.00% |
| Visual | 0.00% | 0.00% | 0.00% | 0.00% | 0.00% |
| Auditory | 100.00% | 100.00% | 100.00% | 100.00% | 100.00% |
| Tactile | 0.00% | 0.00% | 0.00% | 0.00% | 0.00% |
| Single interval | 10.90% | 30.21% | 21.09% | 18.00% | 2.47% |
| Sequence | 89.10% | 69.77% | 78.91% | 76.46% | 97.50% |
| Trajectory | 0.00% | 0.00% | 0.00% | 0.00% | 0.00% |
| Perceptual | 74.41% | 57.73% | 51.42% | 33.83% | 59.05% |
| Motor | 18.10% | 19.71% | 4.85% | 48.60% | 39.95% |
| Quantification | 73.05% | 71.88% | 85.44% | 60.23% | 60.96% |
| Prediction | 12.21% | 2.70% | 0.00% | 17.80% | 24.12% |
| Task control | 0.02% | 16.16% | 7.33% | 14.43% | 2.46% |
| Cognitive load control | 50.19% | 45.59% | 62.61% | 61.54% | 71.65% |
| Stringent control | 43.39% | 28.30% | 16.17% | 20.42% | 25.89% |
| Duration discrimination | 12.60% | 27.34% | 14.15% | 14.38% | 11.79% |
| Temporal orienting | 0.00% | 0.00% | 0.00% | 13.91% | 0.00% |
| Reproduction | 0.00% | 0.00% | 0.00% | 0.00% | 0.00% |
| Rhythm perception | 61.81% | 30.38% | 37.27% | 13.91% | 47.24% |
| Spatiotemporal prediction | 0.00% | 0.00% | 0.00% | 0.00% | 0.00% |
| Spatiotemporal judgement | 0.00% | 0.00% | 0.00% | 0.00% | 0.00% |
| Continuation | 0.00% | 0.00% | 0.13% | 6.04% | 0.91% |
| Synchronization | 0.00% | 0.00% | 0.00% | 17.80% | 24.12% |
| Other tasks | 10.83% | 7.81% | 0.32% | 9.29% | 0.00% |
| Encoding | 0.00% | 0.00% | 0.00% | 0.00% | 0.00% |

A summary of the obtained clusters, including the center coordinates, cluster size, regional cluster extent, contributing studies, and proportional contribution of each temporal characteristic for the auditory modality analysis.

**Supplementary Table 8. Results of the Single Interval Stimuli Analysis**

|  | Cluster 1 | Cluster2 | Cluster 3 | Cluster 4 | Cluster 5 |
| --- | --- | --- | --- | --- | --- |
| Center coordinates | 40/20/2 | -38/18/0 | 42/40/20 | 56/-40/41 | 4/14/52 |
| Cluster size | 673 | 430 | 170 | 180 | 757 |
| Cluster label | Right nsula, frontal operculum cortex, inferior frontal gyrus | Left insula, frontal operculum cortex | Right middle frontal gyrus | Right inferior parietal lobule | Pre-SMA and paracingulate gyrus |
| Contributing articles | Aso2010  Bueti2011  Carlsson2006  Coull2012_2  Coull2012  Coull2004  Ferrandez2003  Gandour2002  Hayashi2013  Khoshnejad2017  Klahr2011  Lewis2002  Lewis2003  Livesey2007  Maquet1996  Morillon2009  Pfeuty2015  Rao2001  Skagerlund2016  Tipples2013  Tregellas2006  Wittmann2010 | Aso2010  Bueti2011  Bueti2008  Carlsson2006  Coull2016  Coull2012  Coull2004  Ferrandez2003  Hayashi2013  Klahr2011  Lewis2003  Livesey2007  Morillon2009  Shih2009  Skagerlund2016  Tipples2013  Tregellas2006  Wittmann2010 | Coull2012_2  Coull2008  Coull2001  Dormal2011  Hayashi2013  Klahr2011  Lewis2002  Lewis2003  Macar2002  Morillon2009  Smith2003  Tipples2013 | Aso2010  Bueti2008  Carlsson2006  Coull2012_2  Coull2012  Coull2004  Dormal2011  Ferrandez2003  Henry2013  Khoshnejad2017  Klahr2011  Lewis2002  Lewis2003  Macar2002  Maquet1996  Morillon2009  Skagerlund2016  Wittmann2010  Bueti2010  Hayashi2015 | Bueti2011  Bueti2008  Coull2012_2  Coull2012  Coull2008  Coull2004  Dormal2011  Ferrandez2003  Gutyrchik2009  Harrington2010  Hayashi2013  Henry2013  Klahr2011  Lewis2002  Lewis2003  Macar2004  Macar2002  Maquet1996  Shih2009  Skagerlund2016  Smith2003  Tipples2013  Tregellas2006  Wittmann2010  Cui2009 |
| Short | 4.18% | 11.33% | 0.02% | 18.86% | 13.29% |
| Medium | 53.16% | 45.48% | 63.04% | 32.22% | 59.33% |
| Long | 31.67% | 23.54% | 26.00% | 36.74% | 20.88% |
| Visual | 72.94% | 70.52% | 88.37% | 71.31% | 68.27% |
| Auditory | 17.15% | 16.00% | 0.01% | 9.60% | 16.45% |
| Tactile | 9.60% | 6.46% | 11.62% | 19.01% | 3.13% |
| Single interval | 100.00% | 100.00% | 100.00% | 100.00% | 100.00% |
| Sequence | 0.00% | 0.00% | 0.00% | 0.00% | 0.00% |
| Trajectory | 0.00% | 0.00% | 0.00% | 0.00% | 0.00% |
| Perceptual | 70.87% | 81.65% | 54.45% | 60.73% | 68.09% |
| Motor | 14.43% | 2.81% | 45.55% | 34.59% | 25.01% |
| Quantification | 92.33% | 93.24% | 73.76% | 92.32% | 90.07% |
| Prediction | 3.48% | 6.76% | 7.41% | 7.35% | 2.80% |
| Task control | 38.27% | 41.39% | 61.93% | 37.98% | 54.74% |
| Cognitive load control | 39.51% | 25.65% | 21.24% | 15.35% | 18.99% |
| Stringent control | 18.69% | 27.17% | 61.93% | 37.01% | 20.08% |
| Duration discrimination | 67.40% | 75.19% | 54.45% | 59.76% | 68.09% |
| Temporal orienting | 3.48% | 6.76% | 7.41% | 7.34% | 2.80% |
| Reproduction | 10.22% | 2.50% | 19.30% | 27.88% | 15.08% |
| Rhythm perception | 0.00% | 0.00% | 0.00% | 0.00% | 0.00% |
| Spatiotemporal prediction | 0.00% | 0.00% | 0.00% | 0.00% | 0.00% |
| Spatiotemporal judgement | 0.00% | 0.00% | 0.00% | 0.00% | 0.00% |
| Continuation | 0.00% | 0.00% | 0.00% | 0.00% | 0.00% |
| Synchronization | 0.00% | 0.00% | 0.00% | 0.00% | 0.00% |
| Other tasks | 0.00% | 0.00% | 0.00% | 0.01% | 0.00% |

A summary of the obtained clusters, including the center coordinates, cluster size, regional cluster extent, contributing studies, and proportional contribution of each temporal characteristic for the single interval stimuli analysis.

**Supplementary Table 9. Results of the Sequence Stimuli Analysis**

|  | Cluster 1 | Cluster2 | Cluster 3 | Cluster 4 | Cluster 5 | Cluster 6 | Cluster 7 | Cluster 8 |
| --- | --- | --- | --- | --- | --- | --- | --- | --- |
| Center coordinates | -32/-66/-28 | 44/16/-4 | -20/6/2 | 22/8/4 | -48/12/0 | 50/12/14 | -44/-48/44 | 0/10/54 |
| Cluster size | 207 | 257 | 147 | 334 | 370 | 217 | 121 | 784 |
| Cluster label | Left cerebellum crus I | Right insula, frontal operculum cortex, inferior frontal gyrus, precentral gyrus | Left putamen, pallidum, and caudate | Right putamen, pallidum, and caudate | Left insula, frontal operculum cortex, inferior frontal gyrus, precentral gyrus | Right inferior frontal gyrus and precentral gyrus | Left inferior parietal sulcus | Pre-SMA, SMA-proper, paracingulate gyrus |
| Contributing articles | Araneda2016  Bengtsson2004  Bengtsson2006  Chen2008_1  Chen2008_2  Grahn2013  Jantzen2007  Jantzen2005  Kawashima2000  Lewis2004  Oullier2005  Schubotz2000  Kung2013  Mayville2002 | Araneda2016  Bengtsson2004  Bengtsson2006  Chen2008_2  Grahn2007  Grahn2013  Jantzen2007  Lejeune1997  Lewis2004  Marchant2013  Schubotz2001  Shergill2006  Tomasi2015  Kung2013  Mayville2002 | Araneda2016  Grahn2007  Grahn2009  Grahn2013  Marchant2013  Schubotz2000  Teki2011  Tomasi2015  Kung2013  Schubotz2001_2  Geiser2012  Matthews2019 | Araneda2016  Bengtsson2004  Garraux2005  Golan2015  Grahn2007  Grahn2009  Grahn2013  Lewis2004  Marchant2013  Schubotz2000  Schubotz2001  Teki2011  Tomasi2015  Kung2013  Mayville2002  Schubotz2001_2  Geiser2012  Matthews2019 | Araneda2016  Bengtsson2004  Bengtsson2006  Grahn2007  Grahn2013  Jahanshahi2010  Kawashima2000  Lewis2004  Marchant2013  Schubotz2000  Schubotz2001  Teki2011  Tomasi2015  Kung2013  Mayville2002  Schubotz2001_2  Schubotz2003  Matthews2019 | Araneda2016  Chen2008_2  Grahn2013  Jahanshahi2010  Konoike2015  Lewis2004  Marchant2013  Schubotz2000  Schubotz2001  Shergill2006  Tomasi2015  Kung2013  Schubotz2001_2  Schubotz2003 | Araneda2016  Chen2008_1  Chen2008_2  Henry2013  Jantzen2007  Konoike2015  Lewis2004  Marchant2013  Oullier2005  Schubotz2000  Bolger2014  Matthews2019 | Araneda2016  Bengtsson2004  Bengtsson2005  Chen2008_1  Chen2008_2  Grahn2013  Henry2013  Jantzen2007  Jantzen2005  Konoike2015  Lejeune1997  Lewis2004  Ortuno2002  Oullier2005  Schubotz2000  Schubotz2001  Shergill2006  Teki2011  Kung2013  Mayville2002  Schubotz2001_2  Matthews2019 |
| Short | 11.50% | 18.75% | 24.10% | 32.41% | 15.46% | 22.34% | 31.13% | 12.05% |
| Medium | 88.50% | 74.18% | 75.89% | 67.30% | 84.54% | 67.66% | 68.87% | 85.04% |
| Long | 0.00% | 7.07% | 0.01% | 0.30% | 0.00% | 10.00% | 0.00% | 2.91% |
| Visual | 4.65% | 26.28% | 22.09% | 13.30% | 27.34% | 21.23% | 0.00% | 7.72% |
| Auditory | 66.76% | 48.39% | 48.50% | 56.46% | 54.37% | 44.99% | 69.23% | 75.37% |
| Tactile | 0.00% | 0.00% | 0.00% | 0.00% | 0.00% | 0.00% | 0.00% | 0.00% |
| Single interval | 0.00% | 0.00% | 0.00% | 0.00% | 0.00% | 0.00% | 0.00% | 0.00% |
| Sequence | 100.00% | 100.00% | 100.00% | 100.00% | 100.00% | 100.00% | 100.00% | 100.00% |
| Trajectory | 0.00% | 0.00% | 0.00% | 0.00% | 0.00% | 0.00% | 0.00% | 0.00% |
| Perceptual | 20.47% | 20.46% | 78.69% | 64.54% | 47.14% | 59.91% | 54.23% | 36.96% |
| Motor | 77.92% | 62.17% | 13.60% | 28.82% | 44.63% | 16.41% | 31.39% | 53.27% |
| Quantification | 68.28% | 34.91% | 70.50% | 68.52% | 62.54% | 82.23% | 68.88% | 59.63% |
| Prediction | 22.60% | 39.30% | 21.69% | 19.50% | 19.47% | 4.47% | 30.46% | 22.38% |
| Task control | 7.10% | 7.00% | 10.30% | 5.98% | 4.83% | 21.80% | 0.37% | 10.58% |
| Cognitive load control | 81.91% | 72.79% | 57.82% | 56.47% | 65.76% | 69.11% | 70.09% | 68.42% |
| Stringent control | 10.99% | 20.21% | 31.87% | 37.55% | 29.41% | 9.09% | 29.54% | 21.00% |
| Duration discrimination | 0.00% | 0.07% | 3.37% | 3.62% | 0.27% | 0.00% | 0.00% | 2.51% |
| Temporal orienting | 0.00% | 29.82% | 13.44% | 11.43% | 19.47% | 4.21% | 16.17% | 0.04% |
| Reproduction | 0.00% | 0.00% | 0.00% | 0.00% | 0.00% | 0.00% | 0.00% | 0.00% |
| Rhythm perception | 20.47% | 20.39% | 75.31% | 60.92% | 46.87% | 59.91% | 54.23% | 34.45% |
| Spatiotemporal prediction | 0.00% | 0.00% | 0.00% | 0.00% | 0.00% | 0.00% | 0.00% | 0.00% |
| Spatiotemporal judgement | 0.00% | 0.00% | 0.00% | 0.00% | 0.00% | 0.00% | 0.00% | 0.00% |
| Continuation | 19.62% | 13.61% | 0.01% | 3.42% | 2.67% | 10.07% | 0.00% | 12.66% |
| Synchronization | 22.60% | 9.47% | 0.00% | 0.00% | 0.00% | 0.26% | 14.30% | 22.34% |
| Other tasks | 28.19% | 0.85% | 0.09% | 8.92% | 12.72% | 0.04% | 0.27% | 5.42% |

A summary of the obtained clusters, including the center coordinates, cluster size, regional cluster extent, contributing studies, and proportional contribution of each temporal characteristic for the sequence stimuli analysis.

# Supplementary Table 10. Results of the Perceptual Tasks Analysis

|  | Cluster 1 | Cluster2 | Cluster 3 | Cluster 4 |
| --- | --- | --- | --- | --- |
| Center coordinates | -34/14/2 | 22/8/4 | 44/20/4 | 0/16/50 |
| Cluster size | 969 | 342 | 768 | 871 |
| Cluster label | Left insula, frontal operculum cortex, inferior frontal gyrus, precentral gyrus, putamen, pallidum, and caudate | Right putamen, pallidum, and caudate | Right insula, frontal operuculum cortex, inferior frontal gyrus, and precentral gyrus | Pre-SMA, SMA-proper, paracingulate gyrus |
| Contributing articles | Araneda2016  Aso2010  Beudel2008  Carlsson2006  Coull2012  Coull2008_2  Coull2004  Ferrandez2003  Grahn2007  Grahn2009  Grahn2013  Gutyrchik2009  Harrington2010  Hayashi2013  Henry2013  Klahr2011  Konoike2015  Lewis2003  Livesey2007  Morillon2009  Schubotz2000  Schubotz2001  Shih2009  Skagerlund2016  Teki2011  Tipples2013  Tregellas2006  Kung2013  Schubotz2001_2  Schubotz2003  Field2005  Geiser2012  Matthews2019 | Araneda2016  Bueti2011  Grahn2007  Grahn2009  Grahn2013  Gutyrchik2009  Morillon2009  Rao2001  Schubotz2000  Schubotz2001  Shih2010  Teki2011  Tipples2013  Tregellas2006  Kung2013  Schubotz2001_2  Geiser2012  Matthews2019 | Araneda2016  Aso2010  Beudel2008  Bueti2011  Carlsson2006  Coull2012  Coull2004  Ferrandez2003  Gandour2002  Grahn2007  Grahn2013  Hayashi2013  Klahr2011  Konoike2015  Lewis2003  Livesey2007  Maquet1996  Morillon2009  O'reilly2008  Pfeuty2015  Rao2001  Schubotz2000  Schubotz2001  Skagerlund2016  Smith2003  Teki2011  Tipples2013  Tregellas2006  Kung2013  Schubotz2001_2  Schubotz2003 | Apaydin2018  Araneda2016  Beudel2008  Bueti2011  Coull2015  Coull2012  Coull2008  Coull2004  Dormal2011  Ferrandez2003  Grahn2013  Gutyrchik2009  Harrington2010  Hayashi2013  Henry2013  Klahr2011  Lewis2003  Maquet1996  O'reilly2008  Schubotz2000  Schubotz2001  Shih2009  Skagerlund2016  Smith2003  Teki2011  Tipples2013  Tregellas2006  Wittmann2010  Kung2013  Schubotz2001_2  Matthews2019 |
| Short | 21.49% | 33.49% | 17.49% | 17.57% |
| Medium | 63.56% | 64.91% | 60.31% | 68.62% |
| Long | 5.87% | 1.60% | 13.40% | 7.18% |
| Visual | 50.36% | 23.82% | 59.40% | 57.54% |
| Auditory | 32.31% | 56.35% | 29.71% | 29.42% |
| Tactile | 2.47% | 0.00% | 3.80% | 0.00% |
| Single interval | 50.53% | 29.21% | 60.75% | 55.27% |
| Sequence | 43.50% | 70.79% | 35.06% | 31.30% |
| Trajectory | 5.30% | 0.00% | 4.18% | 9.50% |
| Perceptual | 100.00% | 100.00% | 100.00% | 100.00% |
| Motor | 0.00% | 0.00% | 0.00% | 0.00% |
| Quantification | 90.89% | 92.26% | 92.01% | 93.70% |
| Prediction | 6.25% | 7.64% | 3.82% | 0.00% |
| Task control | 20.30% | 16.41% | 20.51% | 32.44% |
| Cognitive load control | 38.38% | 42.70% | 54.43% | 36.27% |
| Stringent control | 36.07% | 35.23% | 20.05% | 28.63% |
| Duration discrimination | 50.61% | 29.64% | 57.16% | 58.15% |
| Temporal orienting | 21.49% | 0.00% | 3.80% | 0.00% |
| Reproduction | 0.00% | 0.00% | 0.00% | 0.00% |
| Rhythm perception | 22.85% | 61.57% | 25.68% | 25.67% |
| Spatiotemporal prediction | 2.46% | 0.00% | 0.00% | 0.00% |
| Spatiotemporal judgement | 2.84% | 0.00% | 4.18% | 6.30% |
| Continuation | 0.00% | 0.00% | 0.00% | 0.00% |
| Synchronization | 0.00% | 0.00% | 0.00% | 0.00% |
| Other tasks | 0.00% | 0.00% | 0.00% | 0.00% |
| Encoding | 4.84% | 8.78% | 9.17% | 5.96% |

A

summary of the obtained clusters, including the center coordinates, cluster size, regional cluster extent, contributing studies, and proportional contribution of each temporal characteristic for the perceptual tasks analysis.

**Supplementary Table 11. Results of the Motor Tasks Analysis**

|  | Cluster 1 | Cluster2 | Cluster 3 | Cluster 4 | Cluster 5 | Cluster 6 |
| --- | --- | --- | --- | --- | --- | --- |
| Center coordinates | -30/R-64/-30 | 50/14/2 | -40/16/-4 | 42/40/22 | -48/-40/40 | 4/12/54 |
| Cluster size | 268 | 700 | 279 | 194 | 142 | 667 |
| Cluster label | Left cerebellum curs I, II, VI | Right insula, frontal operculum cortex, inferior frontal gyrus, precentral gyrus | Left insula, frontal operculum cortex, and inferior frontal gyrus | Right middle frontal gyrus | Left inferior parietal sulcus | Pre-SMA and SMA-proper |
| Contributing articles | Aso2010  Bengtsson2004  Bengtsson2006  Bueti2008  Chen2008_1  Chen2008_2  Coull2012_2  Coull1998_1  Jantzen2007  Jantzen2005  Kawashima2000  Lewis2004  Onuki2015  Oullier2005  Kung2013  Mayville2002 | Aso2010  Bengtsson2004  Bengtsson2006  Bueti2011  Carvalho2016  Chen2008_2  Coull2012_2  Garraux2005  Jahanshahi2010  Jantzen2007  Khoshnejad2017  Konoike2015  Lejeune1997  Lewis2002  Lewis2004  Li2015  Marchant2013  Shergill2006  Tomasi2015  Wittmann2010  Kung2013  Mayville2002  Visalli2019  Cui2009 | Bengtsson2004  Bengtsson2006  Bueti2011  Bueti2008  Carvalho2016  Hackley2009  Jahanshahi2010  Lewis2004  Marchant2013  Tomasi2015  Wittmann2010  Billington2010  Kung2013  Mayville2002 | Carvalho2016  Chen2008_1  Chen2008_2  Coull2012_2  Coull2001  Jahanshahi2010  Lewis2002  Macar2002  Ortuno2002  Shergill2006  Billington2010  Kung2013  Mayville2002  Bueti2010 | Bengtsson2006  Carvalho2016  Chen2008_1  Chen2008_2  Coull2016  Coull2012_2  Coull1998_1  Coull2001  Davranche2011  Konoike2015  Lewis2004  Marchant2013  Oullier2005  Bolger2014 | Bengtsson2004  Bengtsson2005  Bueti2008  Carvalho2016  Chen2008_1  Chen2008_2  Coull2012_2  Jantzen2007  Jantzen2005  Konoike2015  Lejeune1997  Lewis2002  Lewis2004  Macar2004  Macar2002  Ortuno2002  Oullier2005  Shergill2006  Wittmann2010  Billington2010  Mayville2002  Cui2009 |
| Short | 0.01% | 6.02% | 12.85% | 5.43% | 14.44% | 3.75% |
| Medium | 99.99% | 55.81% | 72.30% | 73.80% | 68.98% | 67.92% |
| Long | 0.00% | 38.17% | 14.85% | 20.58% | 16.58% | 28.33% |
| Visual | 26.63% | 40.69% | 31.39% | 46.02% | 68.32% | 32.59% |
| Auditory | 55.99% | 36.44% | 51.78% | 47.71% | 17.24% | 52.42% |
| Tactile | 0.00% | 4.89% | 0.00% | 5.52% | 0.00% | 2.87% |
| Single interval | 21.76% | 33.37% | 20.40% | 35.72% | 51.74% | 30.50% |
| Sequence | 77.96% | 55.15% | 65.86% | 48.45% | 31.68% | 58.50% |
| Trajectory | 0.27% | 11.48% | 13.74% | 15.83% | 16.58% | 11.00% |
| Perceptual | 0.00% | 0.00% | 0.00% | 0.00% | 0.00% | 0.00% |
| Motor | 100.00% | 100.00% | 100.00% | 100.00% | 100.00% | 100.00% |
| Quantification | 47.97% | 39.28% | 39.12% | 19.80% | 15.65% | 44.37% |
| Prediction | 28.22% | 49.01% | 42.46% | 52.88% | 74.40% | 33.82% |
| Task control | 19.91% | 29.88% | 13.23% | 28.47% | 9.57% | 28.29% |
| Cognitive load control | 72.29% | 62.05% | 66.36% | 71.33% | 89.67% | 56.49% |
| Stringent control | 7.80% | 6.40% | 20.41% | 0.19% | 0.76% | 11.38% |
| Duration discrimination | 0.00% | 0.00% | 0.00% | 0.00% | 0.00% | 0.00% |
| Temporal orienting | 0.11% | 19.08% | 18.52% | 8.07% | 56.61% | 3.87% |
| Reproduction | 5.95% | 24.86% | 20.34% | 9.51% | 0.00% | 19.01% |
| Rhythm perception | 0.00% | 0.00% | 0.00% | 0.00% | 0.00% | 0.00% |
| Spatiotemporal prediction | 0.27% | 11.48% | 13.74% | 15.83% | 16.58% | 11.00% |
| Spatiotemporal judgement | 0.00% | 0.00% | 0.00% | 0.00% | 0.00% | 0.00% |
| Continuation | 17.38% | 13.55% | 6.08% | 4.74% | 11.31% | 18.19% |
| Synchronization | 27.83% | 18.46% | 10.19% | 28.98% | 1.21% | 18.95% |
| Other tasks | 24.63% | 0.87% | 12.71% | 5.56% | 4.33% | 7.16% |
| Encoding | 0.00% | 0.00% | 0.00% | 0.00% | 0.00% | 0.00% |

A summary of the obtained clusters, including the center coordinates, cluster size, regional cluster extent, contributing studies, and proportional contribution of each temporal characteristic for the motor tasks analysis.

**Supplementary Table 12. Results of the Quantification Tasks Analysis**

|  | Cluster 1 | Cluster2 | Cluster 3 | Cluster 4 | Cluster 5 | Cluster 6 |
| --- | --- | --- | --- | --- | --- | --- |
| Center coordinates | -32/-64/-32 | -38/14/0 | 22/6/2 | 46/18/4 | 2/12/54 | 46/-42/46 |
| Cluster size | 184 | 991 | 464 | 1254 | 1440 | 316 |
| Cluster label | Left cerebellum crus I and II | Left insula, frontal operculum cortex, inferior frontal gyrus, precentral gyrus, putamen, pallidum, and caudate | Right putamen, pallidum, and caudate | Right insula, frontal operculum cortex, inferior frontal gyrus, and precentral gyrus | Pre-SMA, SMA-proper, and paracingulate gyrus | Right inferior parietal sulcus, inferior parietal lobule |
| Contributing articles | Araneda2016  Aso2010  Bengtsson2004  Bengtsson2006  Bueti2008  Coull2012_2  Coull2012  Dormal2011  Grahn2013  Jantzen2007  Jantzen2005  Kawashima2000  Klahr2011  Lewis2003  Lewis2004  Morillon2009  Oullier2005  Schubotz2000  Mayville2002 | Araneda2016  Aso2010  Bengtsson2004  Bengtsson2006  Bueti2011  Bueti2008  Coull2012  Coull2004  Ferrandez2003  Grahn2007  Grahn2009  Grahn2013  Gutyrchik2009  Harrington2010  Hayashi2013  Henry2013  Jahanshahi2010  Kawashima2000  Klahr2011  Konoike2015  Lewis2003  Lewis2004  Livesey2007  Morillon2009  Schubotz2000  Schubotz2001  Shih2009  Skagerlund2016  Teki2011  Tipples2013  Tregellas2006  Wittmann2010  Kung2013  Mayville2002  Schubotz2001_2  Schubotz2003  Matthews2019 | Araneda2016  Bengtsson2004  Bueti2011  Bueti2008  Coull2012_2  Garraux2005  Grahn2007  Grahn2009  Grahn2013  Gutyrchik2009  Khoshnejad2017  Lewis2004  Morillon2009  Rao2001  Schubotz2000  Schubotz2001  Shih2010  Teki2011  Tipples2013  Tregellas2006  Kung2013  Mayville2002  Schubotz2001_2  Matthews2019 | Araneda2016  Aso2010  Bengtsson2004  Bengtsson2006  Bueti2011  Coull2012_2  Coull2012  Coull2004  Ferrandez2003  Gandour2002  Grahn2007  Grahn2013  Hayashi2013  Henry2013  Jahanshahi2010  Khoshnejad2017  Klahr2011  Konoike2015  Lewis2002  Lewis2003  Lewis2004  Livesey2007  Macar2002  Maquet1996  Morillon2009  Ortuno2002  Oullier2005  Pfeuty2015  Rao2001  Schubotz2000  Schubotz2001  Shergill2006  Skagerlund2016  Smith2003  Teki2011  Tipples2013  Tregellas2006  Wittmann2010  Kung2013  Mayville2002  Schubotz2001_2  Schubotz2003 | Araneda2016  Bengtsson2004  Bengtsson2005  Bueti2011  Bueti2008  Coull2015  Coull2012_2  Coull2012  Coull2008  Coull2004  Dormal2011  Ferrandez2003  Grahn2013  Gutyrchik2009  Harrington2010  Hayashi2013  Henry2013  Jantzen2007  Jantzen2005  Klahr2011  Konoike2015  Lewis2002  Lewis2003  Lewis2004  Macar2004  Macar2002  Maquet1996  Ortuno2002  Oullier2005  Schubotz2000  Schubotz2001  Shergill2006  Shih2009  Skagerlund2016  Smith2003  Teki2011  Tipples2013  Tregellas2006  Wittmann2010  Kung2013  Mayville2002  Schubotz2001_2  Matthews2019 | Aso2010  Bueti2011  Bueti2008  Coull2012_2  Coull2004  Dormal2011  Grahn2013  Henry2013  Jantzen2007  Klahr2011  Konoike2015  Lewis2002  Lewis2003  Lewis2004  Macar2002  Maquet1996  Oullier2005  Schubotz2000  Shergill2006  Skagerlund2016  Wittmann2010  Matthews2019 |
| Short | 7.58% | 13.86% | 22.45% | 12.45% | 15.59% | 17.15% |
| Medium | 74.99% | 69.70% | 69.90% | 62.39% | 70.41% | 61.15% |
| Long | 12.30% | 10.36% | 7.65% | 20.96% | 10.63% | 11.34% |
| Visual | 45.06% | 44.01% | 27.10% | 53.20% | 38.52% | 44.14% |
| Auditory | 25.93% | 40.31% | 48.59% | 32.54% | 39.93% | 44.77% |
| Tactile | 0.00% | 0.00% | 5.09% | 3.31% | 2.30% | 1.68% |
| Single interval | 44.55% | 52.70% | 37.41% | 63.18% | 49.96% | 46.81% |
| Sequence | 55.45% | 47.00% | 62.59% | 35.75% | 44.75% | 42.49% |
| Trajectory | 0.00% | 0.00% | 0.00% | 0.00% | 2.24% | 0.00% |
| Perceptual | 47.06% | 76.19% | 70.08% | 66.31% | 63.68% | 50.38% |
| Motor | 0.00 % | 0.00 % | 0.00 % | 0.00 % | 0.00 % | 0.00 % |
| Quantification | 100.00 % | 100.00 % | 100.00 % | 100.00 % | 100.00 % | 100.00 % |
| Prediction | 0.00% | 0.00% | 0.00% | 0.00% | 0.00% | 0.00% |
| Task control | 32.32% | 24.60% | 25.53% | 33.00% | 31.38% | 34.46% |
| Cognitive load control | 47.84% | 37.01% | 34.79% | 44.59% | 38.36% | 31.77% |
| Stringent control | 19.84% | 34.04% | 35.73% | 19.17% | 28.72% | 33.77% |
| Duration discrimination | 29.34% | 44.17% | 23.15% | 43.44% | 39.52% | 25.56% |
| Temporal orienting | 0.00% | 0.00% | 0.00% | 0.00% | 0.00% | 0.00% |
| Reproduction | 13.19% | 2.09% | 11.79% | 10.89% | 10.64% | 15.99% |
| Rhythm perception | 17.71% | 28.14% | 42.46% | 17.54% | 18.61% | 14.12% |
| Spatiotemporal prediction | 0.00% | 0.00% | 0.00% | 0.00% | 0.00% | 0.00% |
| Spatiotemporal judgement | 0.00% | 0.00% | 0.00% | 0.00% | 0.00% | 0.00% |
| Continuation | 20.19% | 7.98% | 7.72% | 8.29% | 13.72% | 14.61% |
| Synchronization | 0.00% | 0.00% | 0.00% | 0.00% | 0.00% | 0.00% |
| Other tasks | 17.33% | 4.74% | 5.95% | 2.90% | 3.11% | 2.98% |
| Encoding | 0.00% | 3.59% | 4.47% | 4.26% | 2.51% | 0.00% |

A summary of the obtained clusters, including the center coordinates, cluster size, regional cluster extent, contributing studies, and proportional contribution of each temporal characteristic for the quantification tasks analysis.

# Supplementary Table 13. Results of the Prediction Tasks Analysis

|  | Cluster 1 | Cluster2 | Cluster 3 | Cluster 4 |
| --- | --- | --- | --- | --- |
| Center coordinates | 40/18/0 | -34/20/2 | -42/-46/40 | 3/12/50 |
| Cluster size | 105 | 167 | 289 | 158 |
| Cluster label | Right insula and frontal operculum | Left insula and frontal operculum cortex | Left inferior parietal sulcus | Pre-SMA, paracingulate gyrus |
| Contributing articles | Carlsson2006  Carvalho2016  Che++n2008_2  Lejeune1997  Marchant2013  Tomasi2015  Kung2013 | Carlsson2006  Carvalho2016  Coull2016  Hackley2009  Marchant2013  Tomasi2015  Billington2010  Kung2013  Field2005 | Assmus2003  Carlsson2006  Carvalho2016  Chen2008_1  Chen2008_2  Coull2016  Coull2012_2  Coull1998_1  Coull2001  Davranche2011  Marchant2013  Bolger2014 | Carvalho2016  Chen2008_1  Chen2008_2  Coull2012_2  Lejeune1997  Billington2010  Cui2009 |
| Short | 6.18% | 42.93% | 11.79% | 10.69% |
| Medium | 69.86% | 26.62% | 73.56% | 51.50% |
| Long | 22.05% | 14.24% | 9.23% | 37.81% |
| Visual | 61.10% | 56.31% | 64.52% | 60.29% |
| Auditory | 30.80% | 12.91% | 18.27% | 39.71% |
| Tactile | 1.91% | 16.21% | 5.42% | 0.00% |
| Single interval | 1.94% | 16.93% | 60.71% | 25.37% |
| Sequence | 77.18% | 37.79% | 30.07% | 47.83% |
| Trajectory | 20.88% | 45.28% | 9.23% | 26.80% |
| Perceptual | 1.91% | 32.92% | 6.03% | 0.00% |
| Motor | 98.09% | 67.08% | 75.71% | 100.00% |
| Quantification | 0.00% | 0.00% | 0.00% | 0.00% |
| Prediction | 100.00% | 100.00% | 100.00% | 100.00% |
| Task control | 1.16% | 0.00% | 14.01% | 19.75% |
| Cognitive load control | 98.81% | 83.30% | 85.99% | 66.59% |
| Stringent control | 0.00% | 16.70% | 0.00% | 0.00% |
| Duration discrimination | 0.00% | 0.00% | 0.00% | 0.00% |
| Temporal orienting | 47.16% | 41.81% | 72.50% | 25.45% |
| Reproduction | 0.00% | 0.00% | 0.00% | 0.00% |
| Rhythm perception | 0.00% | 0.00% | 0.00% | 0.00% |
| Spatiotemporal prediction | 20.88% | 45.28% | 9.23% | 26.80% |
| Spatiotemporal judgement | 0.00% | 0.00% | 0.00% | 0.00% |
| Continuation | 0.00% | 0.00% | 0.00% | 0.00% |
| Synchronization | 31.97% | 12.91% | 18.27% | 47.76% |
| Other tasks | 0.00% | 0.00% | 0.00% | 0.00% |
| Encoding | 0.00% | 0.00% | 0.00% | 0.00% |

A summary of the obtained clusters, including the center coordinates, cluster size, regional cluster extent, contributing studies, and proportional contribution of each temporal characteristic for the prediction tasks analysis.

**Supplementary Table 14. Results of the Task Level of Control Analysis**

|  | Cluster 1 | Cluster2 | Cluster 3 | Cluster 4 | Cluster 5 |
| --- | --- | --- | --- | --- | --- |
| Center coordinates | 36/26/0 | -50/12/8 | 52/14/16 | 40/38/20 | 4/16/50 |
| Cluster size | 103 | 143 | 245 | 96 | 522 |
| Cluster label | Right insula | Left inferior frontal gyrus | Right inferior frontal gyrus and precentral gyrus | Right middle frontal gyrus | Pre-SMA and paracingulate gyrus |
| Contributing articles | Coull2012_2  Ferrandez2003  Khoshnejad2017  Lejeune1997  Maquet1996  Skagerlund2016  Tipples2013  Tregellas2006  Wittmann2010 | Bueti2008  Coull2012_2  Ferrandez2003  Li2015  Schubotz2000  Shih2009  Skagerlund2016  Tipples2013  Tregellas2006  Wittmann2010 | Coull2012_2  Gandour2002  Khoshnejad2017  Li2015  Schubotz2000  Shergill2006  Smith2003  Tipples2013  Tregellas2006  Wittmann2010 | Coull2012_2  Macar2002  Ortuno2002  Shergill2006  Smith2003  Tipples2013 | Bueti2008  Coull2012_2  Dormal2011  Ferrandez2003  Gutyrchik2009  Lejeune1997  Macar2002  Maquet1996  Ortuno2002  Schubotz2000  Shergill2006  Shih2009  Skagerlund2016  Smith2003  Tipples2013  Tregellas2006  Wittmann2010 |
| Short | 14.37% | 11.45% | 10.17% | 0.01% | 9.35% |
| Medium | 82.43% | 68.91% | 43.80% | 73.34% | 76.36% |
| Long | 3.19% | 19.64% | 46.03% | 26.65% | 14.28% |
| Visual | 84.90% | 48.33% | 44.06% | 71.18% | 71.46% |
| Auditory | 14.97% | 30.52% | 28.90% | 2.17% | 16.74% |
| Tactile | 0.13% | 0.00% | 6.13% | 24.06% | 0.46% |
| Single interval | 97.53% | 78.70% | 68.01% | 95.24% | 81.44% |
| Sequence | 2.47% | 20.94% | 20.95% | 4.76% | 18.56% |
| Trajectory | 0.00% | 0.36% | 11.04% | 0.00% | 0.00% |
| Perceptual | 79.63% | 69.29% | 40.84% | 51.60% | 60.38% |
| Motor | 19.77% | 11.43% | 40.47% | 48.40% | 33.33% |
| Quantification | 80.36% | 93.08% | 75.87% | 80.42% | 82.67% |
| Prediction | 2.46% | 0.36% | 11.04% | 0.00% | 6.12% |
| Task control | 100.00% | 100.00% | 100.00% | 100.00% | 100.00% |
| Cognitive load control | 0.00% | 0.00% | 0.00% | 0.00% | 0.00% |
| Stringent control | 0.00% | 0.00% | 0.00% | 0.00% | 0.00% |
| Duration discrimination | 79.63% | 48.36% | 30.10% | 51.60% | 56.21% |
| Temporal orienting | 0.00% | 0.00% | 0.00% | 0.00% | 0.00% |
| Reproduction | 0.13% | 4.50% | 6.13% | 24.06% | 7.73% |
| Rhythm perception | 0.00% | 20.94% | 10.74% | 0.00% | 4.17% |
| Spatiotemporal prediction | 0.00% | 0.36% | 11.04% | 0.00% | 0.00% |
| Spatiotemporal judgement | 0.00% | 0.00% | 0.00% | 0.00% | 0.00% |
| Continuation | 0.01% | 0.00% | 10.21% | 4.76% | 8.27% |
| Synchronization | 2.46% | 0.00% | 0.00% | 0.00% | 6.12% |
| Other tasks | 0.00% | 0.00% | 0.04% | 0.00% | 0.00% |

A summary of the obtained clusters, including the center coordinates, cluster size, regional cluster extent, contributing studies, and proportional contribution of each temporal characteristic for the task level of control analysis.

**Supplementary Table 15. Results of the Cognitive-Load Level of Control Analysis**

|  | Cluster 1 | Cluster2 | Cluster 3 | Cluster 4 | Cluster 5 | Cluster 6 | Cluster 7 | Cluster 8 | Cluster 9 |
| --- | --- | --- | --- | --- | --- | --- | --- | --- | --- |
| Center coordinates | -34/-66/-29 | 20/10/0 | -16/10/-2 | 46/16/4 | -38/18/0 | 44/44/16 | -44/-48/42 | 1/14/52 | 52/-36/46 |
| Cluster size | 124 | 184 | 117 | 767 | 384 | 99 | 241 | 608 | 110 |
| Cluster label | Left cerebellum curs I | Right putamen and caudate | Left putamen, pallidum, and caudate | Right insula, frontal operculum cortex, inferior frontal gyrus, and precentral gyrus | Left insula, frontal operculum cortex, and inferior frontal gyrus | Right middle frontal gyrus | Left inferior parietal sulcus | Pre-SMA and paracingulate gyrus | Right inferior parietal suluc, inferior parietal lobule |
| Contributing articles | Aso2010  Bengtsson2004  Chen2008_1  Chen2008_2  Coull1998_1  Grahn2013  Jantzen2007  Jantzen2005  Kawashima2000  Klahr2011  Oullier2005  Kung2013  Mayville2002 | Bengtsson2004  Grahn2013  Marchant2013  Rao2001  Schubotz2001  Shih2010  Tomasi2015  Tregellas2006  Kung2013  Mayville2002  Schubotz2001_2  Geiser2012  Matthews2019 | Coull2001  Grahn2013  Harrington2010  Hayashi2013  Livesey2007  Marchant2013  Tomasi2015  Tregellas2006  Kung2013  Mayville2002  Schubotz2001_2  Geiser2012  Matthews2019 | Aso2010  Bengtsson2004  Carlsson2006  Carvalho2016  Chen2008_2  Grahn2013  Hayashi2013  Jahanshahi2010  Klahr2011  Konoike2015  Lewis2002  Livesey2007  Marchant2013  Oullier2005  Pfeuty2015  Rao2001  Schubotz2001  Tomasi2015  Tregellas2006  Kung2013  Mayville2002  Schubotz2001_2 | Aso2010  Bengtsson2004  Carlsson2006  Carvalho2016  Coull2016  Grahn2013  Hackley2009  Hayashi2013  Jahanshahi2010  Klahr2011  Livesey2007  Marchant2013  Schubotz2001  Tomasi2015  Tregellas2006  Billington2010  Kung2013  Mayville2002  Schubotz2001_2  Matthews2019 | Carvalho2016  Chen2008_2  Coull2001  Grahn2013  Hayashi2013  Jahanshahi2010  Jantzen2007  Klahr2011  Lewis2002  Billington2010  Kung2013  Mayville2002 | Apaydin2018  Assmus2003  Carlsson2006  Carvalho2016  Chen2008_1  Chen2008_2  Coull2016  Coull1998_1  Coull2001  Davranche2011  Jantzen2007  Konoike2015  Marchant2013  Oullier2005  Bolger2014  Matthews2019 | Apaydin2018  Bengtsson2004  Carvalho2016  Chen2008_1  Chen2008_2  Grahn2013  Harrington2010  Hayashi2013  Jantzen2007  Jantzen2005  Klahr2011  Konoike2015  Lewis2002  Macar2004  Oullier2005  Schubotz2001  Tregellas2006  Billington2010  Kung2013  Mayville2002  Schubotz2001_2  Matthews2019 | Apaydin2018  Aso2010  Carlsson2006  Carvalho2016  Klahr2011  Konoike2015  Lewis2002  Oullier2005  Tomasi2015  Ustun2017  Billington2010 |
| Short | 16.90% | 43.01% | 9.14% | 18.64% | 21.12% | 25.15% | 14.55% | 12.32% | 8.32% |
| Medium | 81.06% | 56.98% | 90.85% | 59.08% | 60.13% | 62.53% | 74.13% | 69.30% | 45.04% |
| Long | 2.04% | 0.01% | 0.00% | 19.14% | 12.34% | 12.31% | 7.96% | 18.38% | 46.04% |
| Visual | 3.96% | 16.56% | 43.97% | 54.35% | 49.52% | 46.06% | 38.75% | 29.36% | 75.26% |
| Auditory | 69.56% | 53.73% | 53.98% | 34.55% | 36.22% | 53.94% | 43.34% | 60.78% | 24.14% |
| Tactile | 0.00% | 0.00% | 0.00% | 3.14% | 6.41% | 0.01% | 3.36% | 0.00% | 0.60% |
| Single interval | 2.75% | 20.51% | 36.92% | 38.57% | 27.96% | 36.16% | 34.15% | 20.54% | 21.73% |
| Sequence | 97.25% | 79.40% | 63.08% | 52.04% | 60.02% | 53.92% | 57.89% | 66.27% | 35.99% |
| Trajectory | 0.00% | 0.09% | 0.00% | 9.38% | 12.02% | 9.92% | 7.69% | 13.19% | 42.28% |
| Perceptual | 18.95% | 56.66% | 65.72% | 48.95% | 44.72% | 32.62% | 23.16% | 37.00% | 21.21% |
| Motor | 79.62% | 31.30% | 21.11% | 30.46% | 42.34% | 53.55% | 56.24% | 50.48% | 52.75% |
| Quantification | 68.51% | 58.96% | 65.21% | 62.19% | 50.10% | 48.12% | 27.29% | 60.35% | 45.21% |
| Prediction | 28.11% | 29.00% | 21.57% | 25.87% | 36.87% | 37.76% | 71.68% | 28.09% | 34.88% |
| Task control | 0.00% | 0.00% | 0.00% | 0.00% | 0.00% | 0.00% | 0.00% | 0.00% | 0.00% |
| Cognitive laod control | 100.00% | 100.00% | 100.00% | 100.00% | 100.00% | 100.00% | 100.00% | 100.00% | 100.00% |
| Stringent control | 0.00% | 0.00% | 0.00% | 0.00% | 0.00% | 0.00% | 34.15% | 0.00% | 0.00% |
| Duration discrimination | 2.04% | 20.50% | 35.45% | 27.01% | 17.67% | 17.03% | 0.05% | 16.59% | 0.66% |
| Temporal orienting | 0.50% | 16.27% | 20.85% | 15.11% | 24.85% | 12.51% | 48.65% | 0.06% | 12.44% |
| Reproduction | 0.00% | 0.00% | 0.00% | 3.74% | 0.00% | 6.62% | 0.00% | 3.94% | 11.36% |
| Rhythm perception | 16.90% | 36.16% | 30.26% | 18.79% | 20.65% | 15.59% | 18.73% | 18.83% | 0.06% |
| Spatiotemporal prediction | 0.00% | 0.09% | 0.00% | 9.37% | 12.02% | 9.92% | 7.10% | 11.62% | 22.39% |
| Spatiotemporal Judgement | 0.00% | 0.00% | 0.00% | 0.01% | 0.00% | 0.00% | 0.86% | 1.58% | 19.89% |
| Continuation | 26.49% | 3.23% | 0.00% | 2.56% | 1.93% | 0.00% | 0.00% | 7.73% | 0.00% |
| Synchronization | 27.61% | 0.00% | 0.00% | 1.38% | 0.00% | 15.33% | 15.93% | 16.41% | 0.05% |
| Other tasks | 22.87% | 11.71% | 0.22% | 1.16% | 6.26% | 8.88% | 0.42% | 7.66% | 7.09% |

A summary of the obtained clusters, including the center coordinates, cluster size, regional cluster extent, contributing studies, and proportional contribution of each temporal characteristic for the cognitive-load level of analysis.

**Supplementary Table 16. Results of the Stringent Level of Control Analysis**

|  | Cluster 1 | Cluster2 |
| --- | --- | --- |
| Center coordinates | -44/18/-2 | 4/2/64 |
| Cluster size | 210 | 106 |
| Cluster label | Left insula and frontal operculum cortex | Pre-SMA |
| Contributing articles | Araneda2016  Bengtsson2006  Beudel2008  Bueti2011  Coull2012  Coull2008_2  Coull2004  Lewis2003  Lewis2004  Morillon2009  Teki2011  Field2005 | Araneda2016  Bengtsson2005  Bueti2011  Coull2015  Coull2012  Coull2008  Coull2004  Henry2013  Lewis2004  Teki2011 |
| Short | 8.40% | 34.81% |
| Medium | 57.32% | 65.12% |
| Long | 15.20% | 0.04% |
| Visual | 56.44% | 18.71% |
| Auditory | 16.66% | 52.34% |
| Tactile | 0.00% | 0.00% |
| Single interval | 45.96% | 35.79% |
| Sequence | 34.07% | 53.51% |
| Trajectory | 19.97% | 1.84% |
| Perceptual | 74.03% | 63.53% |
| Motor | 16.48% | 17.55% |
| Evaluation | 71.47% | 82.68% |
| Prediction | 8.23% | 0.01% |
| Task control | 0.00% | 0.00% |
| Cognitive load control | 0.00% | 0.00% |
| Stringent control | 100.00% | 100.00% |
| Duration discrimination | 36.61% | 44.62% |
| Temporal orienting | 0.00% | 0.00% |
| Reproduction | 0.00% | 0.00% |
| Rhythm perception | 17.44% | 10.04% |
| Spatiotemporal prediction | 8.23% | 0.01% |
| Spatiotemporal judgement | 11.74% | 0.00% |
| Continuation | 0.00% | 0.24% |
| Synchronization | 0.00% | 0.00% |
| Other tasks | 7.92% | 0.00% |

A summary of the obtained clusters, including the center coordinates, cluster size, regional cluster extent, contributing studies, and proportional contribution of each temporal characteristic for the stringent level of control analysis.

# Supplementary Table 17. PRISMA (2020) checklist

| **Section and Topic** | **Item #** | **Checklist item** | **Location where item is reported** |
| --- | --- | --- | --- |
| **TITLE** | | |  |
| Title | 1 | Identify the report as a systematic review. | P1 |
| **ABSTRACT** | | |  |
| Abstract | 2 | See the PRISMA 2020 for Abstracts checklist. | Attachments |
| **INTRODUCTION** | | |  |
| Rationale | 3 | Describe the rationale for the review in the context of existing knowledge. | P2 |
| Objectives | 4 | Provide an explicit statement of the objective(s) or question(s) the review addresses. | P3, P4 |
| **METHODS** | | |  |
| Eligibility criteria | 5 | Specify the inclusion and exclusion criteria for the review and how studies were grouped for the syntheses. | P5 |
| Information sources | 6 | Specify all databases, registers, websites, organisations, reference lists and other sources searched or consulted to identify studies. Specify the date when each source was last searched or consulted. | P5 |
| Search strategy | 7 | Present the full search strategies for all databases, registers and websites, including any filters and limits used. | P5 |
| Selection process | 8 | Specify the methods used to decide whether a study met the inclusion criteria of the review, including how many reviewers screened each record and each report retrieved, whether they worked independently, and if applicable, details of automation tools used in the process. | P5 |
| Data collection process | 9 | Specify the methods used to collect data from reports, including how many reviewers collected data from each report, whether they worked independently, any processes for obtaining or confirming data from study investigators, and if applicable, details of automation tools used in the process. | P5 |
| Data items | 10a | List and define all outcomes for which data were sought. Specify whether all results that were compatible with each outcome domain in each study were sought (e.g. for all measures, time points, analyses), and if not, the methods used to decide which results to collect. | P5 |
|  | 10b | List and define all other variables for which data were sought (e.g. participant and intervention characteristics, funding sources). Describe any assumptions made about any missing or unclear information. | P5 |
| Study risk of bias assessment | 11 | Specify the methods used to assess risk of bias in the included studies, including details of the tool(s) used, how many reviewers assessed each study and whether they worked independently, and if applicable, details of automation tools used in the process. | P6 |
| Effect measures | 12 | Specify for each outcome the effect measure(s) (e.g. risk ratio, mean difference) used in the synthesis or presentation of results. | Max p-value  Supplementary results |
| Synthesis methods | 13a | Describe the processes used to decide which studies were eligible for each synthesis (e.g. tabulating the study intervention characteristics and comparing against the planned groups for each synthesis (item #5)). | P6 |
|  | 13b | Describe any methods required to prepare the data for presentation or synthesis, such as handling of missing summary statistics, or data conversions. | P6 |
|  | 13c | Describe any methods used to tabulate or visually display results of individual studies and syntheses. | P5 |
|  | 13d | Describe any methods used to synthesize results and provide a rationale for the choice(s). If meta-analysis was performed, describe the model(s), method(s) to identify the presence and extent of statistical heterogeneity, and software package(s) used. | P8 |
|  | 13e | Describe any methods used to explore possible causes of heterogeneity among study results (e.g. subgroup analysis, meta-regression). | P6 |
|  | 13f | Describe any sensitivity analyses conducted to assess robustness of the synthesized results. | -- |
| Reporting bias assessment | 14 | Describe any methods used to assess risk of bias due to missing results in a synthesis (arising from reporting biases). | -- |
| Certainty assessment | 15 | Describe any methods used to assess certainty (or confidence) in the body of evidence for an outcome. | -- |
| **RESULTS** | | |  |
| Study selection | 16a | Describe the results of the search and selection process, from the number of records identified in the search to the number of studies included in the review, ideally using a flow diagram. | P5, P8 |
|  | 16b | Cite studies that might appear to meet the inclusion criteria, but which were excluded, and explain why they were excluded. | -- |
| Study characteristics | 17 | Cite each included study and present its characteristics. | Supplementary table 1 |
| Risk of bias in studies | 18 | Present assessments of risk of bias for each included study. | Supplementary table 1 |
| Results of individual studies | 19 | For all outcomes, present, for each study: (a) summary statistics for each group (where appropriate) and (b) an effect estimate and its precision (e.g. confidence/credible interval), ideally using structured tables or plots. | -- |
| Results of syntheses | 20a | For each synthesis, briefly summarise the characteristics and risk of bias among contributing studies. | P22, 23 |
|  | 20b | Present results of all statistical syntheses conducted. If meta-analysis was done, present for each the summary estimate and its precision (e.g. confidence/credible interval) and measures of statistical heterogeneity. If comparing groups, describe the direction of the effect. | P9-11 |
|  | 20c | Present results of all investigations of possible causes of heterogeneity among study results. | -- |
|  | 20d | Present results of all sensitivity analyses conducted to assess the robustness of the synthesized results. | -- |
| Reporting biases | 21 | Present assessments of risk of bias due to missing results (arising from reporting biases) for each synthesis assessed. | -- |
| Certainty of evidence | 22 | Present assessments of certainty (or confidence) in the body of evidence for each outcome assessed. | -- |
| **DISCUSSION** | | |  |
| Discussion | 23a | Provide a general interpretation of the results in the context of other evidence. | P11-20 |
|  | 23b | Discuss any limitations of the evidence included in the review. | P21-22 |
|  | 23c | Discuss any limitations of the review processes used. | -- |
|  | 23d | Discuss implications of the results for practice, policy, and future research. | P22 |
| **OTHER INFORMATION** | | |  |
| Registration and protocol | 24a | Provide registration information for the review, including register name and registration number, or state that the review was not registered. | NA* |
|  | 24b | Indicate where the review protocol can be accessed, or state that a protocol was not prepared. | -- |
|  | 24c | Describe and explain any amendments to information provided at registration or in the protocol. | -- |
| Support | 25 | Describe sources of financial or non-financial support for the review, and the role of the funders or sponsors in the review. |  |
| Competing interests | 26 | Declare any competing interests of review authors. | Title page |
| Availability of data, code and other materials | 27 | Report which of the following are publicly available and where they can be found: template data collection forms; data extracted from included studies; data used for all analyses; analytic code; any other materials used in the review. | Title page |

A checklist of PRISMA recommendations for reporting systematic reviews, elaborated for different sections of the paper. *From:*  Page MJ, McKenzie JE, Bossuyt PM, Boutron I, Hoffmann TC, Mulrow CD, et al. The PRISMA 2020 statement: an updated guideline for reporting systematic reviews. BMJ 2021;372:n71. doi: 10.1136/bmj.n71

* The PROSPERO website refused to register our protocol due to its non-clinical nature of findings

# Supplementary Table 18. PRISMA (2020) abstract checklist

| **Section and Topic** | **Item #** | **Checklist item** | **Reported (Yes/No)** |
| --- | --- | --- | --- |
| **TITLE** | | |  |
| Title | 1 | Identify the report as a systematic review. | Yes |
| **BACKGROUND** | | |  |
| Objectives | 2 | Provide an explicit statement of the main objective(s) or question(s) the review addresses. | Yes |
| **METHODS** | | |  |
| Eligibility criteria | 3 | Specify the inclusion and exclusion criteria for the review. | No |
| Information sources | 4 | Specify the information sources (e.g. databases, registers) used to identify studies and the date when each was last searched. | No |
| Risk of bias | 5 | Specify the methods used to assess risk of bias in the included studies. | No |
| Synthesis of results | 6 | Specify the methods used to present and synthesise results. | Yes |
| **RESULTS** | | |  |
| Included studies | 7 | Give the total number of included studies and participants and summarise relevant characteristics of studies. | Yes |
| Synthesis of results | 8 | Present results for main outcomes, preferably indicating the number of included studies and participants for each. If meta-analysis was done, report the summary estimate and confidence/credible interval. If comparing groups, indicate the direction of the effect (i.e. which group is favoured). | Yes |
| **DISCUSSION** | | |  |
| Limitations of evidence | 9 | Provide a brief summary of the limitations of the evidence included in the review (e.g. study risk of bias, inconsistency and imprecision). | No |
| Interpretation | 10 | Provide a general interpretation of the results and important implications. | Yes |
| **OTHER** | | |  |
| Funding | 11 | Specify the primary source of funding for the review. | Yes |
| Registration | 12 | Provide the register name and registration number. | NA* |

A checklist of PRISMA recommendations for reporting systematic reviews, elaborated for the abstract section. *From:*  Page MJ, McKenzie JE, Bossuyt PM, Boutron I, Hoffmann TC, Mulrow CD, et al. The PRISMA 2020 statement: an updated guideline for reporting systematic reviews. BMJ 2021;372:n71. doi: 10.1136/bmj.n71

* The PROSPERO website refused to register our protocol due to its non-clinical nature of findings

# Supplementary Table 19. Quality assessment score checklist (score 0/0.5/1 per item; total score out of 10)*

| Category 1: Participants |
| --- |
| 1. More than 10 participants were incorporated. |
| 2. Participants were evaluated for psychiatric and medical illnesses and only healthy individuals were included. |
| 3. Important confounds (e.g. reaction time) were controlled either by the control task or statis. |
|  |
| Category 2: Methods for image acquisition and analysis |
| 4. The imaging technique was clearly described so as to be reproduced. |
| 5. Measurements were clearly described so that they could be reproduced. |
| 6. Whole brain analysis was automated with no a priori regional selection. |
| 7. Adjustments were made for multiple statistical comparisons. |
| Category 3: Results and conclusions |
| 8. Statistical parameters for significant and important nonsignificant differences were provided. |
| 9. Conclusions were consistent with the results. |
|  |

The criterea incorporated to assess the quality of the included papers.

*When criteria were partially met, 0.5 points were awarded.

# Supplementary references

1. Apaydın, N., Üstün, S., Kale, E., Çelikağ, İ., Özgüven, H., Baskak, B., & Çiçek, M. (2018). Neural Mechanisms Underlying Time Perception and Reward Anticipation. Frontiers In Human Neuroscience, 12. doi: 10.3389/fnhum.2018.00115
2. Araneda, R., Renier, L., Ebner-Karestinos, D., Dricot, L., & De Volder, A. (2016). Hearing, feeling or seeing a beat recruits a supramodal network in the auditory dorsal stream. European Journal Of Neuroscience, 45(11), 1439-1450. doi: 10.1111/ejn.13349
3. Aso, K., Hanakawa, T., Aso, T., & Fukuyama, H. (2010). Cerebro-cerebellar Interactions Underlying Temporal Information Processing. Journal Of Cognitive Neuroscience, 22(12), 2913-2925. doi: 10.1162/jocn.2010.21429
4. Assmus, A., Marshall, J., Ritzl, A., Noth, J., Zilles, K., & Fink, G. (2003). Left inferior parietal cortex integrates time and space during collision judgments. Neuroimage, 20, S82-S88. doi: 10.1016/j.neuroimage.2003.09.025
5. Bengtsson, S., & Ullén, F. (2006). Dissociation between melodic and rhythmic processing during piano performance from musical scores. Neuroimage, 30(1), 272-284. doi: 10.1016/j.neuroimage.2005.09.019
6. Bengtsson, S., Ehrsson, H., Forssberg, H., & Ullen, F. (2004). Dissociating brain regions controlling the temporal and ordinal structure of learned movement sequences. European Journal Of Neuroscience, 19(9), 2591-2602. doi: 10.1111/j.0953-816x.2004.03269.x
7. Bengtsson, S., Ehrsson, H., Forssberg, H., & Ullén, F. (2005). Effector-independent voluntary timing: behavioural and neuroimaging evidence. European Journal Of Neuroscience, 22(12), 3255-3265. doi: 10.1111/j.1460-9568.2005.04517.x
8. Bengtsson, S., Ullén, F., Henrik Ehrsson, H., Hashimoto, T., Kito, T., & Naito, E. et al. (2009). Listening to rhythms activates motor and premotor cortices. Cortex, 45(1), 62-71. doi: 10.1016/j.cortex.2008.07.002
9. Beudel, M., Renken, R., Leenders, K., & de Jong, B. (2009). Cerebral representations of space and time. Neuroimage, 44(3), 1032-1040. doi: 10.1016/j.neuroimage.2008.09.028
10. Billington, J., Wilkie, R., Field, D., & Wann, J. (2010). Neural processing of imminent collision in humans. Proceedings Of The Royal Society B: Biological Sciences, 278(1711), 1476-1481. doi: 10.1098/rspb.2010.1895
11. Bolger, D., Coull, J., & Schön, D. (2014). Metrical Rhythm Implicitly Orients Attention in Time as Indexed by Improved Target Detection and Left Inferior Parietal Activation. Journal Of Cognitive Neuroscience, 26(3), 593-605. doi: 10.1162/jocn_a_00511
12. Bueti, D., & Macaluso, E. (2011). Physiological correlates of subjective time: Evidence for the temporal accumulator hypothesis. Neuroimage, 57(3), 1251-1263. doi: 10.1016/j.neuroimage.2011.05.014
13. Bueti, D., Bahrami, B., Walsh, V., & Rees, G. (2010). Encoding of Temporal Probabilities in the Human Brain. Journal Of Neuroscience, 30(12), 4343-4352. doi: 10.1523/jneurosci.2254-09.2010
14. Bueti, D., Walsh, V., Frith, C., & Rees, G. (2008). Different Brain Circuits Underlie Motor and Perceptual Representations of Temporal Intervals. Journal Of Cognitive Neuroscience, 20(2), 204-214. doi: 10.1162/jocn.2008.20017
15. Buonomano, D. V., Bramen, J., & Khodadadifar, M. (2009). Influence of the interstimulus interval on temporal processing and learning: testing the state-dependent network model. Philos Trans R Soc Lond B Biol Sci, 364(1525), 1865-1873. doi:10.1098/rstb.2009.0019
16. Carlsson, K., Andersson, J., Petrovic, P., Petersson, K., Öhman, A., & Ingvar, M. (2006). Predictability modulates the affective and sensory-discriminative neural processing of pain. Neuroimage, 32(4), 1804-1814. doi: 10.1016/j.neuroimage.2006.05.027
17. Carvalho, F., Chaim, K., Sanchez, T., & de Araujo, D. (2016). Time-Perception Network and Default Mode Network Are Associated with Temporal Prediction in a Periodic Motion Task. Frontiers In Human Neuroscience, 10. doi: 10.3389/fnhum.2016.00268
18. (a and b). Chen, J., Penhune, V., & Zatorre, R. (2008). Moving on Time: Brain Network for Auditory-Motor Synchronization is Modulated by Rhythm Complexity and Musical Training. Journal Of Cognitive Neuroscience, 20(2), 226-239. doi: 10.1162/jocn.2008.20018
20. Cotti, J., Rohenkohl, G., Stokes, M., Nobre, A., & Coull, J. (2011). Functionally dissociating temporal and motor components of response preparation in left intraparietal sulcus. Neuroimage, 54(2), 1221-1230. doi: 10.1016/j.neuroimage.2010.09.038
21. Coull, J. T., Vidal, F., Nazarian, B., & Macar, F. (2004). Functional Anatomy of the Attentional Modulation of Time Estimation. *Science*, *303*(5663), 1506–1508. https://doi.org/10.1126/science.1091573
22. Coull, J., & Nobre, A. (1998). Where and When to Pay Attention: The Neural Systems for Directing Attention to Spatial Locations and to Time Intervals as Revealed by Both PET and fMRI. The Journal Of Neuroscience, 18(18), 7426-7435. doi: 10.1523/jneurosci.18-18-07426.1998
23. Coull, J., Charras, P., Donadieu, M., Droit-Volet, S., & Vidal, F. (2015). SMA Selectively Codes the Active Accumulation of Temporal, Not Spatial, Magnitude. Journal Of Cognitive Neuroscience, 27(11), 2281-2298. doi: 10.1162/jocn_a_00854
24. Coull, J., Cotti, J., & Vidal, F. (2016). Differential roles for parietal and frontal cortices in fixed versus evolving temporal expectations: Dissociating prior from posterior temporal probabilities with fMRI. Neuroimage, 141, 40-51. doi: 10.1016/j.neuroimage.2016.07.036
25. Coull, J., Davranche, K., Nazarian, B., & Vidal, F. (2013). Functional anatomy of timing differs for production versus prediction of time intervals. Neuropsychologia, 51(2), 309-319. doi: 10.1016/j.neuropsychologia.2012.08.017
26. Coull, J., Hwang, H., Leyton, M., & Dagher, A. (2012). Dopamine Precursor Depletion Impairs Timing in Healthy Volunteers by Attenuating Activity in Putamen and Supplementary Motor Area. Journal Of Neuroscience, 32(47), 16704-16715. doi: 10.1523/jneurosci.1258-12.201
27. Coull, J., Nazarian, B., & Vidal, F. (2008). Timing, Storage, and Comparison of Stimulus Duration Engage Discrete Anatomical Components of a Perceptual Timing Network. Journal Of Cognitive Neuroscience, 20(12), 2185-2197. doi: 10.1162/jocn.2008.20153
28. Coull, J., Nobre, A., & Frith, C. (2001). The Noradrenergic 2 Agonist Clonidine Modulates Behavioural and Neuroanatomical Correlates of Human Attentional Orienting and Alerting. Cerebral Cortex, 11(1), 73-84. doi: 10.1093/cercor/11.1.73
29. Coull, J., Vidal, F., Guolon, C., Nazarian, B., & Craig, C. (2008). Using Time-To-Contact information to assess potential collision modulates both visual and temporal prediction networks. Frontiers In Human Neuroscience, 2. doi: 10.3389/neuro.09.010.2008
30. Cui, X., Stetson, C., Montague, P. R., & Eagleman, D. M. (2009). Ready ... Go: Amplitude Of The Fmri Signal Encodes Expectation Of Cue Arrival Time. PLoS Biol, 7(8). doi:10.1371/journal.pbio.1000167
31. Davranche, K., Nazarian, B., Vidal, F., & Coull, J. (2011). Orienting Attention in Time Activates Left Intraparietal Sulcus for Both Perceptual and Motor Task Goals. Journal Of Cognitive Neuroscience, 23(11), 3318-3330. doi: 10.1162/jocn_a_00030
32. Dormal, V., Dormal, G., Joassin, F., & Pesenti, M. (2011). A common right fronto-parietal network for numerosity and duration processing: An fMRI study. Human Brain Mapping, 33(6), 1490-1501. doi: 10.1002/hbm.21300
33. Eickhoff, S. B., Bzdok, D., Laird, A. R., Kurth, F., & Fox, P. T. (2012). Activation Likelihood Estimation meta-analysis revisited. NeuroImage, 59(3), 2349-2361. doi:10.1016/j.neuroimage.2011.09.017
34. Eickhoff, S. B., Laird, A. R., Grefkes, C., Wang, L. E., Zilles, K., & Fox, P. T. (2009). Coordinate-based activation likelihood estimation meta-analysis of neuroimaging data: a random-effects approach based on empirical estimates of spatial uncertainty. Hum Brain Mapp, 30(9), 2907-2926. doi:10.1002/hbm.20718
35. Eickhoff, S. B., Nichols, T. E., Laird, A. R., Hoffstaedter, F., Amunts, K., Fox, P. T., . . . Eickhoff, C. R. (2016). Behavior, Sensitivity, and power of activation likelihood estimation characterized by massive empirical simulation. NeuroImage, 137, 70-85. doi:10.1016/j.neuroimage.2016.04.072
36. Evans, A., Collins, L., Mills, S. R., Brown, E. D., Kelly, R. L., & Peters, T. (1993). 3D Statistical Neuroanatomical Models from 305 MRI Volumes (Vol. 1813–1817).
37. Ferrandez, A., Hugueville, L., Lehéricy, S., Poline, J., Marsault, C., & Pouthas, V. (2003). Basal ganglia and supplementary motor area subtend duration perception: an fMRI study. Neuroimage, 19(4), 1532-1544. doi: 10.1016/s1053-8119(03)00159-9
38. Field, D., & Wann, J. (2005). Perceiving Time to Collision Activates the Sensorimotor Cortex. Current Biology, 15(5), 453-458. doi: 10.1016/j.cub.2004.12.081
39. Gandour, J., Wong, D., Lowe, M., Dzemidzic, M., Satthamnuwong, N., Tong, Y., & Li, X. (2002). A Cross-Linguistic fMRI Study of Spectral and Temporal Cues Underlying Phonological Processing. Journal Of Cognitive Neuroscience, 14(7), 1076-1087. doi: 10.1162/089892902320474526
40. Garraux, G., Mckinney, C., Wu, T., Kansaku, K., Nolte, G., & Hallett, M. (2005). Shared Brain Areas But Not Functional Connections Controlling Movement Timing and Order. Journal Of Neuroscience, 25(22), 5290-5297. doi: 10.1523/jneurosci.0340-05.2005
41. Geiser, E., Notter, M., & Gabrieli, J. (2012). A Corticostriatal Neural System Enhances Auditory Perception through Temporal Context Processing. Journal Of Neuroscience, 32(18), 6177-6182. doi: 10.1523/jneurosci.5153-11.2012
42. Godde, B., Diamond, M., & Braun, C. (2010). Feeling for space or for time: Task-dependent modulation of the cortical representation of identical vibrotactile stimuli. Neuroscience Letters, 480(2), 143-147. doi: 10.1016/j.neulet.2010.06.027
43. Golan, R., & Zakay, D. (2015). The duality of temporal encoding – the intrinsic and extrinsic representation of time. Frontiers In Psychology, 6. doi: 10.3389/fpsyg.2015.01288
44. Grahn, J., & Brett, M. (2007). Rhythm and Beat Perception in Motor Areas of the Brain. Journal Of Cognitive Neuroscience, 19(5), 893-906. doi: 10.1162/jocn.2007.19.5.893
45. Grahn, J., & Rowe, J. (2009). Feeling the Beat: Premotor and Striatal Interactions in Musicians and Nonmusicians during Beat Perception. Journal Of Neuroscience, 29(23), 7540-7548. doi: 10.1523/jneurosci.2018-08.2009
46. Grahn, J., & Rowe, J. (2012). Finding and Feeling the Musical Beat: Striatal Dissociations between Detection and Prediction of Regularity. Cerebral Cortex, 23(4), 913-921. doi: 10.1093/cercor/bhs083
47. Grondin, S., Ouellet, B., & Roussel, M. E. (2004). Benefits and limits of explicit counting for discriminating temporal intervals. Can J Exp Psychol, 58(1), 1-12. doi:10.1037/h0087436
48. Grube, M., Cooper, F. E., Chinnery, P. F., & Griffiths, T. D. (2010). Dissociation of duration-based and beat-based auditory timing in cerebellar degeneration. Proc Natl Acad Sci U S A, 107(25), 11597-11601. doi:10.1073/pnas.0910473107
49. Gutyrchik, E., Churan, J., Meindl, T., Bokde, A. L. W., von Bernewitz, H., Born, C., Reiser, M., Pöppel, E., & Wittmann, M. (2010). Functional neuroimaging of duration discrimination on two different time scales. Neuroscience Letters, 469(3), 411–415. https://doi.org/10.1016/j.neulet.2009.12.040
50. Hackley, S. A., Langner, R., Rolke, B., Erb, M., Grodd, W., & Ulrich, R. (2009). Separation of phasic arousal and expectancy effects in a speeded reaction time task via fMRI. Psychophysiology, 46(1), 163–171. https://doi.org/10.1111/j.1469-8986.2008.00722.x
51. Harnett, N., Shumen, J., Wagle, P., Wood, K., Wheelock, M., Baños, J., & Knight, D. (2016). Neural mechanisms of human temporal fear conditioning. Neurobiology Of Learning And Memory, 136, 97-104. doi: 10.1016/j.nlm.2016.09.019
52. Harrington, D., Zimbelman, J., Hinton, S., & Rao, S. (2009). Neural Modulation of Temporal Encoding, Maintenance, and Decision Processes. Cerebral Cortex, 20(6), 1274-1285. doi: 10.1093/cercor/bhp194
53. Hayashi, M., Ditye, T., Harada, T., Hashiguchi, M., Sadato, N., & Carlson, S. et al. (2015). Time Adaptation Shows Duration Selectivity in the Human Parietal Cortex. PLOS Biology, 13(9), e1002262. doi: 10.1371/journal.pbio.1002262
54. Hayashi, M., Kanai, R., Tanabe, H., Yoshida, Y., Carlson, S., Walsh, V., & Sadato, N. (2013). Interaction of Numerosity and Time in Prefrontal and Parietal Cortex. Journal Of Neuroscience, 33(3), 883-893. doi: 10.1523/jneurosci.6257-11.2013
55. Henry, M., Herrmann, B., & Obleser, J. (2013). Selective Attention to Temporal Features on Nested Time Scales. Cerebral Cortex, 25(2), 450-459. doi: 10.1093/cercor/bht240
56. Jahanshahi, M., Jones, C., Dirnberger, G., & Frith, C. (2006). The Substantia Nigra Pars Compacta and Temporal Processing. Journal Of Neuroscience, 26(47), 12266-12273. doi: 10.1523/jneurosci.2540-06.2006
57. Jahanshahi, M., Jones, C., Zijlmans, J., Katzenschlager, R., Lee, L., & Quinn, N. et al. (2010). Dopaminergic modulation of striato-frontal connectivity during motor timing in Parkinson's disease. Brain, 133(3), 727-745. doi: 10.1093/braiNAwq012
58. Jäncke, L., Loose, R., Lutz, K., Specht, K., & Shah, N. (2000). Cortical activations during paced finger-tapping applying visual and auditory pacing stimuli. Cognitive Brain Research, 10(1-2), 51-66. doi: 10.1016/s0926-6410(00)00022-7
59. Jantzen, K., Oullier, O., Marshall, M., Steinberg, F., & Kelso, J. (2007). A parametric fMRI investigation of context effects in sensorimotor timing and coordination. Neuropsychologia, 45(4), 673-684. doi: 10.1016/j.neuropsychologia.2006.07.020
60. Jantzen, K., Steinberg, F., & Kelso, J. (2005). Functional MRI reveals the existence of modality and coordination-dependent timing networks. Neuroimage, 25(4), 1031-1042. doi: 10.1016/j.neuroimage.2004.12.029
61. Kawashima, R., Okuda, J., Umetsu, A., Sugiura, M., Inoue, K., & Suzuki, K. et al. (2000). Human Cerebellum Plays an Important Role in Memory-Timed Finger Movement: An fMRI Study. Journal Of Neurophysiology, 83(2), 1079-1087. doi: 10.1152/jn.2000.83.2.1079
62. Khoshnejad, M., Roy, M., Martinu, K., Chen, J., Cohen-Adad, J., Grondin, S., & Rainville, P. (2017). Brain processing of the temporal dimension of acute pain in short-term memory. PAIN, 158(10), 2001-2011. doi: 10.1097/j.pain.0000000000001003
63. Klahr, N., Wright, P., Lu, G., Merlo, L., Zhang, Y., & He, G. et al. (2011). Investigating the effects of low dose alcohol on neural timing using functional MRI. Journal Of Magnetic Resonance Imaging, 34(5), 1045-1052. doi: 10.1002/jmri.22747
64. Konoike, N., Kotozaki, Y., Jeong, H., Miyazaki, A., Sakaki, K., & Shinada, T. et al. (2015). Temporal and Motor Representation of Rhythm in Fronto-Parietal Cortical Areas: An fMRI Study. PLOS ONE, 10(6), e0130120. doi: 10.1371/journal.pone.0130120
65. Kung, S., Chen, J., Zatorre, R., & Penhune, V. (2013). Interacting Cortical and Basal Ganglia Networks Underlying Finding and Tapping to the Musical Beat. Journal Of Cognitive Neuroscience, 25(3), 401-420. doi: 10.1162/jocn_a_00325
66. Lejeune, H., Maquet, P., Bonnet, M., Casini, L., Ferrara, A., & Macar, F. et al. (1997). The basic pattern of activation in motor and sensory temporal tasks: positron emission tomography data. Neuroscience Letters, 235(1-2), 21-24. doi: 10.1016/s0304-3940(97)00698-8
67. Lewis, P. A., & Miall, R. C. (2003). Distinct systems for automatic and cognitively controlled time measurement: evidence from neuroimaging. Curr Opin Neurobiol, 13(2), 250-255. doi:10.1016/s0959-4388(03)00036-9
68. Lewis, P., & Miall, R. (2002). Brain activity during non-automatic motor production of discrete multi-second intervals. Neuroreport, 13(14), 1731-1735. doi: 10.1097/00001756-200210070-00008
69. Lewis, P., & Miall, R. (2003). Brain activation patterns during measurement of sub- and supra-second intervals. Neuropsychologia, 41(12), 1583-1592. doi: 10.1016/s0028-3932(03)00118-0
70. Lewis, P., Wing, A., Pope, P., Praamstra, P., & Miall, R. (2004). Brain activity correlates differentially with increasing temporal complexity of rhythms during initialisation, synchronisation, and continuation phases of paced finger tapping. Neuropsychologia, 42(10), 1301-1312. doi: 10.1016/j.neuropsychologia.2004.03.001
71. Li, C., Chen, K., Han, H., Chui, D., & Wu, J. (2012). An fMRI Study of the Neural Systems Involved in Visually Cued Auditory Top-Down Spatial and Temporal Attention. Plos ONE, 7(11), e49948. doi: 10.1371/journal.pone.0049948
72. Li, Y., Mo, L., & Chen, Q. (2015). Differential contribution of velocity and distance to time estimation during self-initiated time-to-collision judgment. Neuropsychologia, 73, 35-47. doi: 10.1016/j.neuropsychologia.2015.04.017
73. Livesey, A., Wall, M., & Smith, A. (2007). Time perception: Manipulation of task difficulty dissociates clock functions from other cognitive demands. Neuropsychologia, 45(2), 321-331. doi: 10.1016/j.neuropsychologia.2006.06.033
74. Lutz, K., Specht, K., Shah, N., & Jäncke, L. (2000). Tapping movements according to regular and irregular visual timing signals investigated with fMRI. Neuroreport, 11(6), 1301-1306. doi: 10.1097/00001756-200004270-00031
75. Macar, F., Anton, J., Bonnet, M., & Vidal, F. (2004). Timing functions of the supplementary motor area: an event-related fMRI study. Cognitive Brain Research, 21(2), 206-215. doi: 10.1016/j.cogbrainres.2004.01.005
76. Macar, F., Lejeune, H., Bonnet, M., Ferrara, A., Pouthas, V., Vidal, F., & Maquet, P. (2002). Activation of the supplementary motor area and of attentional networks during temporal processing. Experimental Brain Research, 142(4), 475-485. doi: 10.1007/s00221-001-0953-0
77. Maquet, P., Lejeune, H., Pouthas, V., Bonnet, M., Casini, L., & Macar, F. et al. (1996). Brain Activation Induced by Estimation of Duration: A PET Study. Neuroimage, 3(2), 119-126. doi: 10.1006/nimg.1996.0014
78. Marchant, J., & Driver, J. (2012). Visual and Audiovisual Effects of Isochronous Timing on Visual Perception and Brain Activity. Cerebral Cortex, 23(6), 1290-1298. doi: 10.1093/cercor/bhs095
79. Matthews, T., Witek, M., Lund, T., Vuust, P., & Penhune, V. (2020). The sensation of groove engages motor and reward networks. Neuroimage, 214, 116768. doi: 10.1016/j.neuroimage.2020.116768
80. Mayville, J., Jantzen, K., Fuchs, A., Steinberg, F., & Kelso, J. (2002). Cortical and subcortical networks underlying syncopated and synchronized coordination revealed using fMRI. Human Brain Mapping, 17(4), 214-229. doi: 10.1002/hbm.10065
81. Merchant, H., & Honing, H. (2013). Are non-human primates capable of rhythmic entrainment? Evidence for the gradual audiomotor evolution hypothesis. Front Neurosci, 7, 274. doi:10.3389/fnins.2013.00274
82. Morillon, B., Kell, C., & Giraud, A. (2009). Three Stages and Four Neural Systems in Time Estimation. Journal Of Neuroscience, 29(47), 14803-14811. doi: 10.1523/jneurosci.3222-09.2009
83. Nani, A., Manuello, J., Liloia, D., Duca, S., Costa, T., & Cauda, F. (2019). The Neural Correlates of Time: A Meta-analysis of Neuroimaging Studies. J Cogn Neurosci, 31(12), 1796-1826. doi:10.1162/jocn_a_01459
84. Onuki, Y., Van Someren, E., De Zeeuw, C., & Van der Werf, Y. (2013). Hippocampal–Cerebellar Interaction During Spatio-Temporal Prediction. Cerebral Cortex, 25(2), 313-321. doi: 10.1093/cercor/bht221
85. O'Reilly, J., Mesulam, M., & Nobre, A. (2008). The Cerebellum Predicts the Timing of Perceptual Events. Journal Of Neuroscience, 28(9), 2252-2260. doi: 10.1523/jneurosci.2742-07.2008
86. Ortuño, F., Ojeda, N., Arbizu, J., López, P., Martı́-Climent, J., Peñuelas, I., & Cervera, S. (2002). Sustained Attention in a Counting Task: Normal Performance and Functional Neuroanatomy. Neuroimage, 17(1), 411-420. doi: 10.1006/nimg.2002.1168
87. Oullier, O., Jantzen, K., Steinberg, F., & Kelso, J. (2004). Neural Substrates of Real and Imagined Sensorimotor Coordination. Cerebral Cortex, 15(7), 975-985. doi: 10.1093/cercor/bhh198
88. Penhune, V., Zatorre, R., & Evans, A. (1998). Cerebellar Contributions to Motor Timing: A PET Study of Auditory and Visual Rhythm Reproduction. Journal Of Cognitive Neuroscience, 10(6), 752-765. doi: 10.1162/089892998563149
89. Pfeuty, M., Dilharreguy, B., Gerlier, L., & Allard, M. (2014). fMRI identifies the right inferior frontal cortex as the brain region where time interval processing is altered by negative emotional arousal. Human Brain Mapping, 36(3), 981-995. doi: 10.1002/hbm.22680
90. Rammsayer, T., & Pichelmann, S. (2018). Visual-auditory differences in duration discrimination depend on modality-specific, sensory-automatic temporal processing: Converging evidence for the validity of the Sensory-Automatic Timing Hypothesis. Quarterly Journal of Experimental Psychology, 71(11), 2364-2377. doi:10.1177/1747021817741611
91. Rao, S., Mayer, A., & Harrington, D. (2001). The evolution of brain activation during temporal processing. Nature Neuroscience, 4(3), 317-323. doi: 10.1038/85191
92. Schubotz, R., & Cramon, D. (2001). Interval and Ordinal Properties of Sequences Are Associated with Distinct Premotor Areas. Cerebral Cortex, 11(3), 210-222. doi: 10.1093/cercor/11.3.210
93. Schubotz, R., & von Cramon, D. (2001). Functional organization of the lateral premotor cortex: fMRI reveals different regions activated by anticipation of object properties, location and speed. Cognitive Brain Research, 11(1), 97-112. doi: 10.1016/s0926-6410(00)00069-0
94. Schubotz, R., Friederici, A., & Yves von Cramon, D. (2000). Time Perception and Motor Timing: A Common Cortical and Subcortical Basis Revealed by fMRI. Neuroimage, 11(1), 1-12. doi: 10.1006/nimg.1999.0514
95. Schubotz, R., von Cramon, D., & Lohmann, G. (2003). Auditory what, where, and when: a sensory somatotopy in lateral premotor cortex. Neuroimage, 20(1), 173-185. doi: 10.1016/s1053-8119(03)00218-0
96. Schwartze, M., Rothermich, K., & Kotz, S. A. (2012). Functional Dissociation Of Pre-Sma And Sma-Proper In Temporal Processing. NeuroImage, 60(1), 290-298. doi:10.1016/j.neuroimage.2011.11.089
97. Shergill, S., Tracy, D., Seal, M., Rubia, K., & McGuire, P. (2006). Timing of covert articulation: An fMRI study. Neuropsychologia, 44(12), 2573-2577. doi: 10.1016/j.neuropsychologia.2006.04.005
98. Shih, L., Kuo, W., Yeh, T., Tzeng, O., & Hsieh, J. (2009). Common neural mechanisms for explicit timing in the sub-second range. Neuroreport, 20(10), 897-901. doi: 10.1097/wnr.0b013e3283270b6e
99. Shih, L., Yeh, T., Kuo, W., Tzeng, O., & Hsieh, J. (2010). Effect of temporal difficulty on cerebrocerebellar interaction during visual duration discrimination. Behavioural Brain Research, 207(1), 155-160. doi: 10.1016/j.bbr.2009.10.001
100. Skagerlund, K., Karlsson, T., & Träff, U. (2016). Magnitude Processing in the Brain: An fMRI Study of Time, Space, and Numerosity as a Shared Cortical System. Frontiers In Human Neuroscience, 10. doi: 10.3389/fnhum.2016.00500
101. Smith, A., Taylor, E., Lidzba, K., & Rubia, K. (2003). A right hemispheric frontocerebellar network for time discrimination of several hundreds of milliseconds. Neuroimage, 20(1), 344-350. doi: 10.1016/s1053-8119(03)00337-9
102. Spencer, R. M., Karmarkar, U., & Ivry, R. B. (2009). Evaluating dedicated and intrinsic models of temporal encoding by varying context. Philos Trans R Soc Lond B Biol Sci, 364(1525), 1853-1863. doi:10.1098/rstb.2009.0024
103. Talairach, J., & Tournoux, P. (1988). Co-planar Stereotaxic Atlas of the Human Brain: 3-dimensional Proportional System : an Approach to Cerebral Imaging: G. Thieme.
104. Teghil, A., Boccia, M., D'Antonio, F., Di Vita, A., de Lena, C., & Guariglia, C. (2019). Neural substrates of internally-based and externally-cued timing: An activation likelihood estimation (ALE) meta-analysis of fMRI studies. Neurosci Biobehav Rev, 96, 197-209. doi:10.1016/j.humov.2018.07.00510.1016/j.neubiorev.2018.10.003
105. Teki, S., Grube, M., Kumar, S., & Griffiths, T. (2011). Distinct Neural Substrates of Duration-Based and Beat-Based Auditory Timing. Journal Of Neuroscience, 31(10), 3805-3812. doi: 10.1523/jneurosci.5561-10.2011
106. Tipples, J., Brattan, V., & Johnston, P. (2013). Neural Bases for Individual Differences in the Subjective Experience of Short Durations (Less than 2 Seconds). Plos ONE, 8(1), e54669. doi: 10.1371/journal.pone.0054669
107. Tomasi, D., Wang, G., Studentsova, Y., & Volkow, N. (2014). Dissecting Neural Responses to Temporal Prediction, Attention, and Memory: Effects of Reward Learning and Interoception on Time Perception. Cerebral Cortex, 25(10), 3856-3867. doi: 10.1093/cercor/bhu269
108. Tregellas, J., Davalos, D., & Rojas, D. (2006). Effect of task difficulty on the functional anatomy of temporal processing. Neuroimage, 32(1), 307-315. doi: 10.1016/j.neuroimage.2006.02.036
109. Turkeltaub, P. E., Eickhoff, S. B., Laird, A. R., Fox, M., Wiener, M., & Fox, P. (2012). Minimizing within-experiment and within-group effects in Activation Likelihood Estimation meta-analyses. Hum Brain Mapp, 33(1), 1-13. doi:10.1002/hbm.21186
110. Üstün, S., Kale, E., & Çiçek, M. (2017). Neural Networks for Time Perception and Working Memory. Frontiers In Human Neuroscience, 11. doi: 10.3389/fnhum.2017.00083
111. Visalli, A., Capizzi, M., Ambrosini, E., Mazzonetto, I., & Vallesi, A. (2019). Bayesian modeling of temporal expectations in the human brain. Neuroimage, 202, 116097. doi: 10.1016/j.neuroimage.2019.116097
112. Wiener, M., Lee, Y., Lohoff, F., & Coslett, H. (2014). Individual differences in the morphometry and activation of time perception networks are influenced by dopamine genotype. Neuroimage, 89, 10-22. doi: 10.1016/j.neuroimage.2013.11.019
113. Wiener, M., Turkeltaub, P., & Coslett, H. B. (2010a). The image of time: a voxel-wise meta-analysis. NeuroImage, 49(2), 1728-1740. doi:10.1016/j.neuroimage.2009.09.064
114. Wiener, M., Turkeltaub, P., & Coslett, H. B. (2010b). Implicit Timing Activates The Left Inferior Parietal Cortex. Neuropsychologia, 48(13), 3967-3971. doi:10.1016/j.neuropsychologia.2010.09.014
115. Wittmann, M., Simmons, A. N., Aron, J. L., & Paulus, M. P. (2010). Accumulation of neural activity in the posterior insula encodes the passage of time. *Neuropsychologia*, *48*(10), 3110–3120. <https://doi.org/10.1016/j.neuropsychologia.2010.06.023>
